# The effect of early oral postoperative feeding on the recovery of intestinal motility after gastrointestinal surgery: a systematic review and meta-analysis of randomized clinical trials

**DOI:** 10.3389/fnut.2024.1369141

**Published:** 2024-05-16

**Authors:** Federica Canzan, Jessica Longhini, Arianna Caliaro, Maria Luisa Cavada, Elisabetta Mezzalira, Salvatore Paiella, Elisa Ambrosi

**Affiliations:** ^1^Department of Diagnostics and Public Health, University of Verona, Verona, Italy; ^2^Azienda Ospedaliera Universitaria Integrata Verona, Verona, Italy; ^3^Scuola Provinciale Superiore di Sanità, Bolzano, Italy; ^4^Department of General and Pancreatic Surgery, University of Verona, Verona, Italy

**Keywords:** early feeding, oral feeding, gastrointestinal surgery, ileus, length of hospital stay

## Abstract

**Background and aims:**

Postoperative ileus is a frequent condition, leading to complications and a longer hospital stay. Few studies have demonstrated the benefit of early oral feeding in preventing ileus after gastrointestinal surgery. This study aims to evaluate the efficacy of early versus delayed oral feeding on the recovery of intestinal motility, length of hospital stay, and complications.

**Methods:**

We conducted a systematic review and meta-analysis of randomized control trials, searching PubMed, Embase, Cumulative Index to Nursing and Allied Health Literature, Cochrane Central Register of Controlled Trials, and the ClincalTrials.gov until 31 December 2022. We evaluated the first passage of the stool, the first flatus, complications, length of postoperative stay, and vomiting. We assessed the risk of bias using the Cochrane risk of bias tool (version 2) for randomized trials and the quality of evidence using the Grading of Recommendations Assessment, Development, and Evaluation methodology.

**Results:**

We included 34 studies with a median sample size of 102 participants. With a moderate certainty of the evidence, the early oral feeding may reduce the time taken for the first passage of the stool (MD −0.99 days; CI 95% −1.25, −0.72), the first flatus (MD −0.70 days; CI 95% -0.87, −0.53), and the risk of complications (RR 0.69; CI 95% 0.59–0.80), while with a low certainty of evidence, it may reduce the length of stay (MD −1.31 days; CI 95% −1.59, −1.03). However, early feeding likely does not affect the risk of vomiting (RR 0.90; CI 95% 0.68, 1.18).

**Conclusion:**

This review suggests that early oral feeding after gastrointestinal surgery may lead to a faster intestinal recovery, shorter postoperative stays, and fewer complications. However, careful interpretation is needed due to high heterogeneity and the moderate-to-low quality of evidence. Future studies should focus on the type and starting time of early oral feeding.

## 1 Introduction

Postoperative ileus (POI) is an iatrogenic condition after gastrointestinal surgery, defined as transient deceleration or cessation of intestine motility due to a chain reaction caused by the surgical intervention and the manipulation of the digestive tract (1, 2). Indeed, POI results from a pathophysiological process mainly distinguished in an early neurogenic phase that suppresses enteric neural reflex pathways and a second immunological and inflammatory phase that usually leads to prolonged POI (2, 3).

This condition can manifest with multiple symptoms, such as intolerance to oral intake, nausea, vomiting, and failure to pass flatus or stool (4, 5). No consensus is currently available on the time of physiological restoration of normal intestinal motility (4, 6). However, some studies report that the reappearance of bowel sounds, passing of gas or stool, and tolerance to food and fluids indicate POI resolution (5, 7).

Despite several pathophysiological and treatment studies available in the literature, POI is still a common condition after gastrointestinal surgery, with an estimated prevalence ranging from 17 to 80% (8). Different studies have demonstrated the association between POI and increased health complications, such as nutritional requirements and protein deficiency, pneumonia, anastomotic failure, renal and hepatic failure, delayed autonomy recovery, and mortality (6, 9–11), leading to prolonged hospital stay and readmission (6). This results in high healthcare costs in radiology, laboratory, staffing, and medication costs (12).

Different interventions have been developed and tested over the years to reduce this phenomenon, focusing on every phase of the surgical intervention: preoperative, perioperative, and postoperative periods. The interventions refer to the use of minimally invasive procedures (13), along with proper perioperative fluid management based on “Goal-Directed Therapy” (14), and the use of opioid-free local epidural anesthetics (15), with a demonstrated effectiveness for POI prevention. In addition, the “enhanced recovery after surgery” (ERAS) program, which has been intensively studied and globally recognized for its positive effects in accelerating postoperative recovery (16–18), strongly recommends early feeding strongly during the postoperative phase (19). Early feeding by mouth prevents significant metabolic changes such as insulin resistance (20) and facilitates surgical wound healing. Moreover, compared to parenteral nutrition, it enables the gastrointestinal system to regain its functions faster by stimulating motility and accelerating the first passage of flatus and stool (21). Although early oral feeding might be considered a safe intervention for POI prevention, there is no conclusive evidence of its safety for gastrointestinal function and postoperative complications due to different aspects (9, 22). For instance, a standard definition of “early feeding” is currently missing, and the type of feeding reported in the studies has been poorly described. For example, it is unclear whether the feeding involves liquids or solid food. Consequently, the guidelines do not provide clear indications regarding when and what to administer in the early feeding phase. As a result, the indications may vary widely across surgical settings and cultures. In addition, the direct association between early feeding and POI has been partially investigated. Available reviews have considered feeding as a component of ERAS and multimodal programs (23) or as a single intervention (22, 24–30). Therefore, no single review has yet determined the impact of early oral feeding as a single or a combined intervention.

Moreover, most previous systematic reviews considered only colorectal (23, 26), lower (22), upper gastrointestinal surgery (24, 25), or cancer indication to surgery (24) and assessed the impact of early feeding on outcome such as the length of stay (LOS), complications (22, 25), or nutritional status (24). In two reviews with POI as the primary outcome (26, 27), the POI was measured only by time to first flatus or bowel movement, the intervention was focused only on diet as a single intervention (26) or fluids (27), and the last search on databases was in June 2019 (26) and September 2020 (27).

Therefore, this study aims to evaluate the efficacy of early versus delayed oral feeding on the recovery of intestinal motility as the primary outcome to fill the gaps described above.

## 2 Materials and methods

We performed a systematic review and meta-analysis according to the Cochrane guidelines (31) and reported it following the Preferred Reporting Items for Systematic Reviews and Meta-Analyses (PRISMA) guidelines (32). We searched PubMed, Embase, Cumulative Index to Nursing and Allied Health Literature, Cochrane Central Register of Controlled Trials (CENTRAL), and the ClincalTrials.gov register from inception to 31 December 2022. We also searched System for Information on Grey Literature (SIGLE) to identify further studies or papers that were not published, checked the references of articles included and relevant reviews on the topic, and contacted the corresponding authors to clarify doubts and consider unpublished data. The search in the databases and registries was conducted using both free texts and MeSH and EMTREE terms by adopting the search strings reported in Supplementary File S1. We prospectively registered the protocol in the International Prospective Register of Systematic Reviews (CRD42022298777) and published it (with references blinded for the reviewer).

### 2.1 Inclusion and exclusion criteria

We included randomized clinical trials (RCTs) that met the following inclusion criteria: (i) aimed at comparing the effect of early postoperative (fluids and food by mouth within 24 h) versus delayed oral feeding; (ii) treated patients >18 years of age undergoing both elective and emergency gastrointestinal surgery; (iii) assessed intestinal recovery outcomes, complications, and LOS after gastrointestinal surgery; (iv) published in English, Italian, and German. The primary outcome is the time to the first passage of stool, while secondary outcomes include the time to first flatus, LOS, and any negative effects, such as nausea, vomiting, infection, organ failure, and major complications, as classified according to the Clavien-Dindo Classification (33). Studies were excluded if the intervention involved the exclusive use of the nasogastric tube. Moreover, studies referring to patients treated for bariatric surgery, appendectomy, and hemorrhoid surgery were excluded. Studies involving gynecological procedures were also excluded.

### 2.2 Selection process

The records identified through the search methods were transferred to Excel® (Microsoft Corporation, Redmond, WA) spreadsheets and then uploaded to Covidence. First, two review authors (blinded for review) independently screened titles and abstracts and then performed full-text revision. A third review author (blinded for review) resolved any disagreements.

### 2.3 Data extraction and management

For each study, data were extracted by two independent authors using electronic data collection forms in Covidence. The extracted data included article references (first author, journal, and year), setting, research methods (study design, total duration of the study, and washout period), type of surgery (emergency or elective surgery); participant characteristics (age and sex), intervention (experimental and control), study’s primary and secondary outcomes, main results, and free notes. We dealt with missing data by contacting the authors of the trials to retrieve relevant information.

### 2.4 Risk of bias assessment

Two independent reviewers performed the quality and risk of bias assessment of the included studies using the revised Cochrane risk of bias tool for randomized trials (RoB2) (34), and a third reviewer solved any disagreements. The risk of bias in each study was classified as high, low, or moderate according to the overall grade agreed upon by the reviewers (34).

### 2.5 Data analysis

The mean difference (MD) with 95% confidence intervals (CIs) was calculated to estimate the effect size of the continuous variables, including the first passage of the stool, first flatus, and LOS. The risk ratio with 95% CI was calculated to estimate the risk likelihood of incurring postoperative complications and vomiting episodes. The risk of complication was defined based on the number of patients who underwent at least one postoperative complication, since it was not possible to classify the complications according to the Clavien-Dindo Classification due to missing information in most of the articles.

A random-effect meta-analysis was conducted for all outcomes, considering the differences in intervention characteristics identified during the data collection and extraction process.

The statistical heterogeneity was assessed by visual inspection of the forest plot and applying the I2 statistic with the Q statistic test. Values greater than 75% were considered as expressing considerable heterogeneity (31).

We performed two subgroup analyses to explore the source of considerable heterogeneity according to the types of interventions and the surgery site. Types of interventions were classified into two categories, namely “early feeding” and “multimodal interventions or ERAS interventions.” The category of multimodal interventions includes studies that investigated programs composed of early feeding and other elements, such as fast-track, preoperative routine changes in feeding, and early mobilization. Regarding the surgery sites, we grouped studies into two categories, one targeting only patients undergoing colon and rectal surgery, while the other including a broader site definition (bowel and abdominal surgeries) or different sites (gastric surgery).

To corroborate the results of the overall analysis, a sensitivity analysis was performed by removing studies at high risk of bias and studies with a sample smaller than 100 participants.

We assessed the publication bias through the funnel plot inspections, and for continuous outcomes, we assessed using Egger’s test. The analysis was performed with RevMan 5.4 (35) and R software (36). The results reported in the included studies as median and interquartile ranges were described narratively.

### 2.6 Summary of evidence

The quality of evidence was evaluated for all outcomes by adopting the Grading of Recommendations Assessment, Development, and Evaluation (GRADE) working group methodology (37). The level of evidence certainly was considered ‘high’, ‘moderate’, ‘low’, or ‘very low’ based on the risk of bias, inconsistency, indirectness, imprecision, publication bias, and additional domains.

## 3 Results

### 3.1 Characteristics of studies

**Figure 1:**
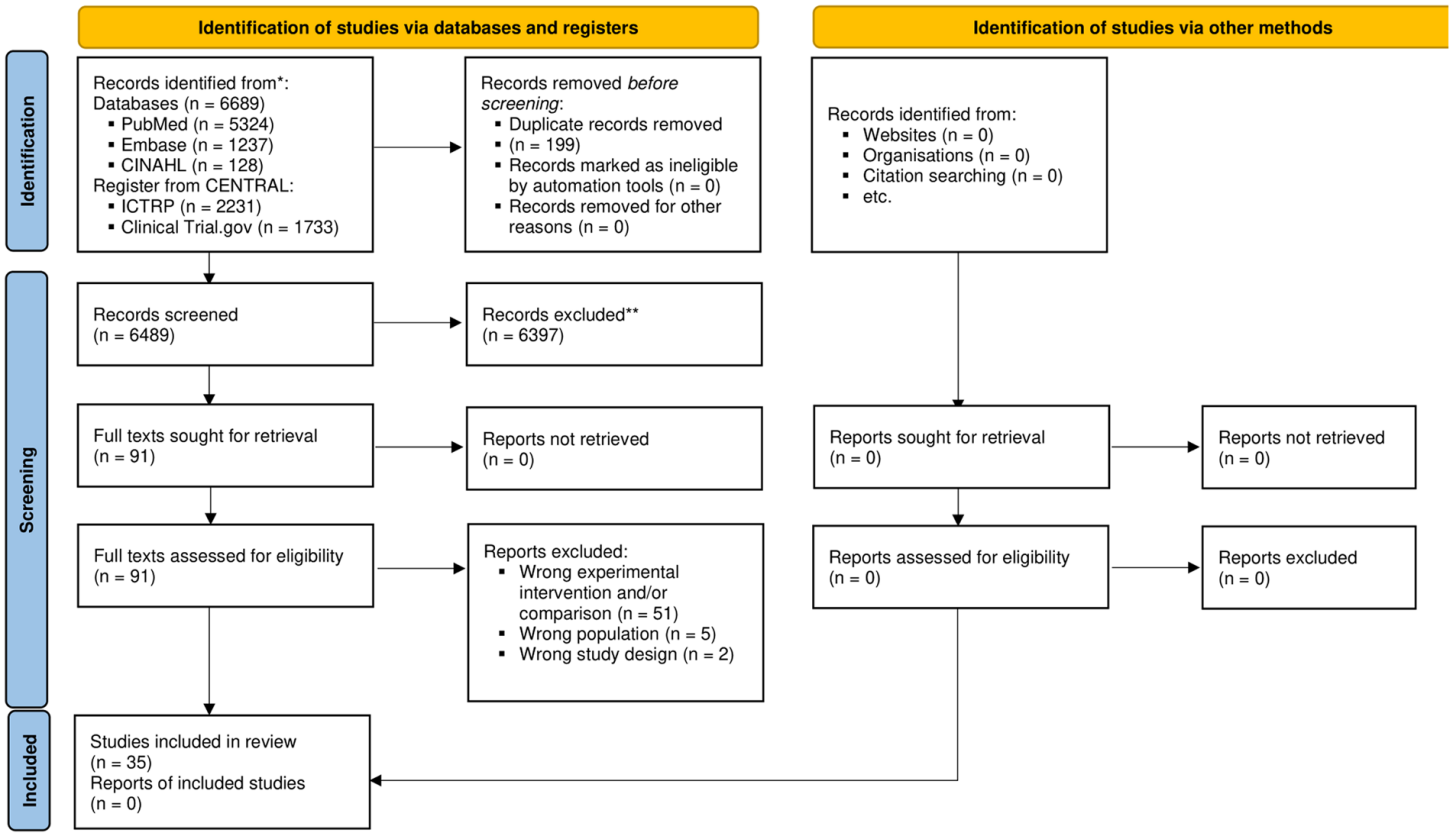
PRISMA 2020 flow diagram for new systematic reviews, which included searches of databases, registers, and other sources. CENTRAL, Cochrane Central Register of Controlled Trials; CINAHL, Cumulative Index to Nursing and Allied Health Literature; ICTRP, International Clinical Trials Registry Platform.

After removing duplicates, we retrieved 6,490 records, of which 35 (38–72) were included (Figure 1; Supplementary File S2: List of excluded studies). The majority of RCTs were conducted in China (15, 44.1%), followed by Korea (4, 11.8%) (Table 1). The median sample size of the studies was 101 participants (IQR, 80–185, min =29, max = 1735). Furthermore, 12 studies evaluated the ERAS protocol, 11 studies evaluated the effectiveness of early feeding interventions, and the remaining 9 studies evaluated multimodal interventions (Table 1). The multimodal interventions included components similar to the ERAS protocol, for example, diet changes in the preoperative phase (53), the use of chewing gum and appetite stimulation programs (56), and early mobilization (48) (Table 1). Nineteen studies included patients undergoing colon and rectal surgery, while another other eight included patients undergoing gastric surgery. The remaining studies referred to the bowel (50, 71) or abdominal (56) surgery, including hepatectomy (51), liver resection (57), cholecystectomy (51), and pancreaticoduodenectomy (61).

**Table 1:** Characteristics of the included studies.

Author, year, country Time of RCT	Intervention (G1)	Control (G2)	Study population	Sample size	Outcomes investigated
**Early feeding**					
Reissman et al. (1995) Florida November 1992–April 1994	Clear liquid diet on the first PO day and regular diet within the next 24 to 48 h, as tolerated (absence of vomiting or abdominal distention). The NGT was removed immediately after surgery	NPO until the resolution of the ileus, then a clear liquid diet, followed by a regular diet. The NGT was removed immediately after surgery	Ad undergoing elective laparotomy with bowel resection	161 G1: 80 G2: 81	NA LOS Complications
Ortiz et al. (1996) Spain Not specified	The NGT was removed in the post-anesthesia care unit. On the PO evening, patients were allowed to intake clear liquids; this continued until the first PO day, at which time they progressed to a regular diet as desired.	The NGT was removed when the surgeon considered that the PO ileus had resolved (indicated by the return of bowel sounds, the absence of nausea, vomiting, and the passage of flatus or stool). At the time of ileus resolution, patients were started on a diet of clear liquids; if this was tolerated for 24 h, then they were advanced to a regular diet.	Ad undergoing elective colon or rectal surgery	190 G1: 93 (2 excluded) G1:95	NA Vomiting Complications
Hartsell et al. (1997) Texas May 1995–February 1996	POD 1: full liquid diet. If the patient consumed 1,000 mL in a 24-h period, he was advanced to a regular diet the next day.	After the return to normal bowel function with passage of flatus or stool, patients began a full liquid diet: if the patient consumed 1,000 mL in a 24-h period, he was advanced to a regular diet the next day.	Ad undergoing elective colorectal surgery	58 G1: 29 G2:29	NA LOS Nausea/Vomiting Complications
Stewart et al. (1998) Australia Not specified	Free fluids from 4 h after the operation and progressed to a solid diet from the first PO day at their own discretion.	Fasting until passage of flatus or bowel motion and was then commenced on clear fluids and progressed to a solid diet over 24–48 h at the surgeon’s discretion	Ad undergoing elective colorectal resection with anastomosis	80 G1: 40 G2:40	NA Time to first flatus LOS Nausea/Vomiting Complications
Zhou et al. (2006) China January–September 2005	Nasogastric tubes were removed within 12–24 h after the operation. The patients were immediately provided with water and gradually transitioned to a liquid fibreless diet after 1 day, followed by a semi-liquid fiber diet after 3 days.	Nasogastric tubes were removed upon the report of passage of flatus by the patient, usually within 3–5 days after surgery.	Ad receiving excision and anastomosis for colorectal tumor.	316 G1: 161 G2: 155	Time to first defecation Time to first flatus LOS Complications
El Nakeeb et al. (2009) Egypt June 2005–April 2008	Early feeding: patients began fluids on the first PO day and advanced to a regular diet within the next 24–48 h, as tolerated (indicated by an absence of vomiting or abdominal distension)	Regular feeding: NPO until the resolution of ileus, then a fluid diet, followed by a regular diet.	Ad undergoing elective open colonic anastomosis	120 G1: 60 G2: 60	Time to first defecation Time to first flatus LOS Vomiting Complications
Consoli et al. (2010) Brazil July 2006–January 2008	Post operatively, on the 1 day, patients in the early fed group (EF) received 500 mL of restricted fluid as the first intake, and if no nausea and vomits were observed, they were able to eat a free diet immediately thereafter.	The traditional care group (trad) received nil by mouth until flatus or evacuation happened.	Ad undergoing elective laparoscopic colonic resection with primary anastomosis	29 G1: 15 G2: 14	NA Time to first flatus PO; Hospital stay Nausea/vomiting Diarrhea Complications
Da Fonseca et al. (2010) Brazil May 2006–February 2009	Early feeding group (EFG): POD1 patients received an oral liquid diet (approximately 500 cm3) and were advanced to a regular diet within the next 24 h, as tolerated (absence of vomiting or abdominal distention) and at their discretion.	Traditional care group (TCG): patients received NPO until the elimination of the first flatus and then received an oral liquid diet, followed by a regular diet within the next 24 h, as described for the EFG.	Ad undergoing elective colonic surgery.	54 G1: 24 (3 excluded) G2: 26 (1 excluded)	NA Time to first flatus Nausea/Vomiting LOS Complications
Dag et al. (2011) Turkey August 2007–September 2009	EOF—fluid diet 12 h after the operation; this was gradually increased to a solid diet as tolerated by the patient.	Fasting until the patient passes first flatus or stools.	Ad undergoing elective open colorectal cancer surgery	199 G1: 99 G2: 100	Time to first defecation LOS Complications
Pragatheeswarane et al. (2014) India September 2011–July 2013	Early oral feeding (EOF)—The nasogastric tube was removed within 24 h of recovery from anesthesia; clear liquid diet of 30 cm3 /h at the 24th h—advanced to 60 cm3 /h in the next 12 h—full fluid diet within 48 h—solid diet over the next 24 h	Traditional oral feeding (TOF)—NPO until the resolution of the ileus, then a clear liquid diet, progressing to a solid diet as tolerated	Elective bowel surgeries	120 G1: 60 G2: 60	Time to first defecation Time to first flatus Vomiting LOS Complications
Wu et al. (2019), China February 2015–August 2017	Water was provided by nurses in the PACU if patients were fully conscious, had stable vital signs, had grade 5 muscle strength, and had well-recovered cough and swallowing reflex. Total water volume 3 mL/kg. The first test volume of water administered was 1 to 5 mL; if negative, patients drank the remaining volume of water by themselves	Patients could not drink water until 4 h after surgery	Ad in the PACU who had undergone elective laparoscopic cholecystectomy	1735 G1: 867 G2: 868	NA Nausea/Vomiting
**Multimodal intervention**					
Feo et al. (2004) Italy March 2000–July 2002	No NGT for decompression. POD 1: liquids, POD 2: soft diet, regardless of the passage of flatus, POD 3: solid diet as tolerated. NG tube was inserted after two PO episodes of vomiting.	NGT for decompression. After the first flatus, patients were gradually given oral feeding from a liquid diet to a soft and solid diet as tolerated. The NG tube was reinserted after two episodes of vomiting that occurred after its removal.	Ad undergoing elective laparotomy colorectal resection for cancer	100 G1: 50 G2: 50	Time to first defecation LOS Nausea/Vomiting Complications
Khoo et al. (2007) United Kingdom May 2003–October 2004	Multimodal group: Nasogastric tubes were removed in the recovery room; diet was allowed immediately after the operation; Patients received regular domperidone, magnesium hydroxide 8%, and liquid protein/calorie supplements from admission.	Conventional care: nasogastric tubes were removed the following morning unless there was 200 mL of free drainage overnight. The diet was commenced only upon observing signs of returning bowel motility	Ad undergoing elective colorectal resection for cancer	81 G1:41 G2:40	Time to first defecation LOS Complications
Ionescu et al. (2009) Romania October 2006–May 2007	Fast track Group: Day of surgery: Fluids if tolerated (no NG tube unless severe PONV) POD1: fluids, Solid food (yogurt and cheese) POD2: Solid food (normal feeding)	Conventional care: Day of surgery: Nasogastric tube, nil by mouth POD1: Nasogastric tube, nil by mouth POD2: If bowel passage occurs, remove the nasogastric tube, and start fluids orally; if not, retain the nasogastric tube	Ad who underwent elective open colorectal surgery for neoplasm	96 G1: 48 G2: 48	NA LOS Nausea/vomiting Complications
Liu et al. (2010) China June 2006–January 2007	Multimodal Optimization of Surgical Care: Early (free fluids on the day of surgery followed by a regular diet as tolerated). Day of surgery: Oral intake of clear fluid ≈50 mL + GS 10% 1,000 mL and GN 500 mL (IV) Sit in bed for about 20 min. POD 1: Semiliquid diet 50–100 mL + GS 10% 1,000 mL and GN 500 mL Stand out of bed for at least 20 min; POD2: Semiliquid diet 100–200 mL + GS 10% 500 mL and GN 500 mL Walk the length of the ward for at least 1 h; POD3: semiliquid diet 200–400 mL + GS 10% 500 mL and GN 500 mL; POD 4: Semiliquid diet; POD 5: Solid diet	Conventional care: NPO until bowel venting. Day of surgery: NPO + GS 10% 1,000 mL and GN 1500 mL (IV); POD1: NPO + GS 10% 1,000 mL and GN 1500 mL; POD2: NPO + GS 10% 1,000 mL and GN 1500 mL; POD3: Usually the presence of bowel flatus; remove the nasogastric tube. Diet and IV fluid reintroduced in the same manner as in the optimized group on PODs 1, 2, and 3; POD5: Semiliquid diet; POD6: Solid diet	Ad between 28 and 81 years of age who underwent gastrectomy procedures.	63 G1: 33 G2: 30	NA LOS Diarrhea/vomiting Complications
Lee et al. (2011) Korea September 2007 and October 2009	Rehabilitation program: Day of surgery: Sit in a chair for <1 h Sips of water <1; POD 1: Sit in chair for >3 h; ward ambulation >400 m; mobilize in bed Semifluid diet >1 L; POD 2: Ward ambulation >600 m; soft blend diet or regular diet; use the laxative routinely.	Conventional care: Day of surgery: Bed rest; Nothing by mouth; POD 1: Sit in chair for >1 h; mobilize in bed; NPO until flatus; POD 2: Ward ambulation >400 m; sips of water if bowel passage occurs; use the laxative if necessary.	Ad who had received laparoscopic colon surgery	100 G1: 46 G2:54	Time to first defecation Time to first flatus LOS Complications
Wang et al. (2012) China April 2006–October 2009	Fast track group: Early food intake—Water when patients returned to consciousness, fluid diet on the POD1 increased in the following days; normal diet on POD 3 and edible oil to facilitate defecation.	Conventional care: Fluid diet was fed after the passage of the first flatus.	Age > 65 years with colorectal cancer and undergoing laparoscopic colorectal resection	78 G1:40 G2:38	NA Time to first flatus LOS Complications
Lee et al. (2013) Korea July 2007–September 2011	Rehabilitation program: Day of surgery: Sips of water \1 L POD1: Semi-fluid diet [1 L]; POD 2: Soft blend diet or regular diet	Day of surgery: NPO; POD 1: Nil by mouth until flatus POD2: Sips of water if bowel passage occurs	Ad aged 20 to 80 years underwent laparoscopic low anterior resection with a defunctioning ileostomy for rectal adenocarcinomas	98 G1: 52 G2:46	Time to first defecation Time to first flatus LOS Complications
Li et al. (2014) China January 2011–February 2012	Fast-track group: POD1: with or without NGT in after 12 h; early oral feeding of water or tea at 12 h, use of EN emulsion (Fresubin®), 50% of total dose in 24 h (Total energy: 25–30 kcal/kg·d); no regular parenteral nutrition support; POD2: fluid restriction to 1,000 mL/kg·d, 100% total dose of EN in 48 h. (Total energy was 25–30 kcal/kg·d); POD3-5: fluid restriction to 500 mL/d	Conventional care: The NGT remains; NPO until flatus, sips of water if bowel passage occurs; transfuse fluid for patients at approximately 3,000 mL/kg until they intake food; TPN; oral feeding after aerocluxus.	Ad with colorectal cancer underwent colorectal surgery	445 G1: 208 G2: 237	Time to first defecation Time to first flatus LOS Complications
Feng et al. (2016) China August 2014–March 2015	Fast-track group: POD 1: If an NGT was placed, remove it after 12 h; Early oral feeding of water or tea at 12 h: oral feeding of emulsion (Fresubin®), 50% of total dose over 24 h (total energy: 25–30 kcal/kg · day); POD 2: Fluid restriction to 1,000 mL/kg · day; Normal diet or emulsion (100% of total dose over 48 h; total energy of 25–30 kcal/kg day; POD 3–4: Fluid restriction to 500 mL/day.	Conventional care: The NGT kept in place, nil by mouth until flatus; sips of water if bowel passage occurs, fluid transfusion (approximately 3,000 mL/kg day) until food intake begins, TPN, oral feeding after aerocluxus.	Ad between the age of 18 and 70 years who underwent colorectal surgery	241 G1: 121 analysis (n = 116) G2: 120 Analysis (n = 114)	Time to first defecation Time to first flatus LOS Complications
Shichinohe eta al. (2017) Japan Not specified	Elental® following the protocol: POD 0: 300 mL, commencing 5 h after the operation and when the patient was able to sit up. POD 1–2: water and ED 900 mL/day; peripheral parenteral nutrition 500 mL; POD 3: ED 300–900 mL with the start of dietary intake of hospital food; POD 4: medium solid diet. Elental®: The composition of solution prepared is 1 kcaL/mL, 906 mOsm/kg, and a 300 mL solution (1 package) contains 63.41 g carbohydrates (provided as dextrin), 13.14 g amino acids (provided as 17 amino acids including 9 essential amino acids), 0.51 g of fat, and vitamins and minerals	POD 0: nil *per OS* and peripheral parenteral nutrition as needed; POD 1–2: Peripheral parenteral nutrition 2000 mL. POD 3: Peripheral parenteral nutrition 1,000 mL; POD 4: normal diet.	Ad under 76 years of age diagnosed colorectal cancer located in the colon and the rectosigmoid, planned laparoscopic surgery, histologically proven colorectal adenocarcinoma.	102 G1: 45 Allocated 51 G2: 49 Allocated 51	Time to first defecation Time to flatus LOS Nausea/Vomiting Complications
Sun et al. (2017) China April 2014–April 2016	Multimodal early oral nutrition: (1) chewing sugar-free gum (30 min 3 times per day) until first defecation; (2) appetite stimulation (including playing a favorite food-related media program) [30 min 3 times/day], seeing colors of and tasting favorite foods [5 min at least 3–4 times/day], watching other people dine [15 min 3 times/day] until first defecation; (3) drinking water immediately on waking and drinking 100 mL juice (orange juice, apple juice or grape juice, containing 30 g of glucose) 6 h after surgery, oral administration of 300 mL enteral nutrition suspension (Peptisorb liquid, Nutricia) divided into 4–5 administrations from 12 h after surgery; enteral nutrition 500 mL at 24 h after surgery, and oral intake gradually increased Day of surgery: 6 h after the operation, parenteral nutrition started in both groups. From POD2 to POD7, parenteral nutrition was initiated after 6 p.m. if oral nutrition was not sufficient.	Conventional care: patients were sent to the ward, intake of water and 300 mL enteral nutrition suspension (Peptisorb liquid, Nutricia) that was divided into 4–5 administrations was commenced after the first defecation, and oral intake was gradually increased. Intake of water after the operation according to the patients’ wishes. Both regimens were isonitrogenous [0.2 g/kg (±0.01 Kcal) (±5%)] and isocaloric [24 Kcal/kg (±1.2 Kcal) (±5%)]. Vitamins and electrolytes were added as required.	Ad undergoing major abdominal surgery	107 G1:53 G2:54	Time to first defecation Time to flatus LOS
Wendler et al. (2022) Brazil Not specified	24-h postoperative period: liquid diet +1,000 mL of ringer lactate solution, 1,000 mL of glucose solution, antibiotic prophylaxis (Kefazol 1 g 8/8 h), analgesics and antiemetics, when needed. 48 h postoperative period: restricted liquid diet 36 h postoperative period: 500 mL of ringer lactate, 500 mL of glucose solution, analgesics, and antiemetics if necessary.	24-h postoperative period: fasting +2000 mL of physiologic solution, 1,000 mL of glucose solution, antibiotic prophylaxis (Kefazol 1 g 8/8 h), analgesics, and antiemetics, if needed. 48 h postoperative period: restricted liquid diet 36 h postoperative period: 1000 mL of physiological solution, 1,000 mL of glucose solution, analgesics, and antiemetics, if needed.	Patients indicated for Roux-en-Y gastro jejunal Bypass, BMI > 35 kg/m^2^+ hypertension and/or diabetes or BMI 40–46 kg/m^2^, surgical time < 120 min + procedure by the same team.	80 G1:40 G2:40	NA Time to first flatus Nausea
**ERAS**					
Ren et al. (2012) China July 2007–May 2010	ERAS protocol. Oral intake of carbohydrate-loaded liquids until 2 h before surgery, drinking 500 mL of water as early as 6 h after surgery, increased to 1,000 mL combined with 500 mL of nutritional supplements on each POD. The patients shifted to a clear liquid diet after the first flatus.	The control patients underwent preoperative fasting and did not start oral intake (diet consisted of full liquids) until the first flatus after surgery.	Ad between 20 and 80 years of age who underwent open radical resection for colorectal cancer.	676 (79 excluded) G1: 299 G2:298	NA Time to first flatus LOS Complications
Abdikarim et al. (2015) China June 2010–December 2012	ERAS protocol. Intraoperative: No NGT or drainage tube; POD 1: Soluble contrast swallow study is done to check the anastomosis. If intact, fluids are started; POD 2: Patient started on soft food. POD 3: Patient progresses to solid food.	Conventional: Intraoperative: routine use of abdominal drainage tubes. POD 1: keep NPO; POD 2: NPO; POD 3: Remove NGT and liquids started POD 4: solid food intake.	Gastric cancer Ad, under 75 years of age, who underwent elective laparoscopic-assisted radical gastrectomy.	61 G1: 30 G2:31	Time to first defecation LOS Complications
He et al. (2015) China April 2014–October 2014	ERAS group: Water intake began at 4 h after surgery and liquid diet restored 12 h after surgery.	Conventional care: if the gastrointestinal tract restores peristalsis, anus exhaust, and defecation without abdominal pain or abdominal distension, patients can be advanced to feed liquid food, then gradually to ordinary food.	Ad undergoing laparoscopic hepatectomy.	99 G1:50 Analyzed 48 G2:49 Analyzed 38	NA Time to first flatus LOS Complications
Mari et al. (2016) Italy Not specified	ERAS protocol. POD 0: start of oral feeding and removal of the nasogastric tube	Conventional care. POD0: The NGT kept in place, nil by mouth; POD1: start of oral feeding and removal of the nasogastric tube	Ad aged over 70 years undergoing elective colorectal laparoscopic surgery	83 G1:40 38 analyzed G2:43	NA Time to first flatus LOS Complications
Liang et al. (2018) China August 2015–June 2016	ERAS protocol. POD 0: drink water 6 h after surgery; Restricted ev fluid; PONV evaluated and multimodal PONV prophylaxis; POD 1: Oral nutritional supplements (liquid) or semi-liquid diet; restricted ev fluid; POD 2: oral semi-liquid diet; stop maintenance ev fluid; POD 3: normal diet	Conventional. POD 0: fast; fluid therapy at the direction of the medical team (2500–3,000 mL); PONV drugs or used based on the symptom of PONV; POD 1: fast or liquid if gastrointestinal function was recovered; POD 2: liquid; POD 3: liquid or semi-liquid diet.	Ad between the age of 16 and 85 years who underwent laparoscopic liver resection.	126 G1: allocated 60, analyzed 58 G2: allocated 66, analyzed 61	NA LOS Nausea/Vomiting Complications
Mingjie et al. (2017) China September 2013–August 2014	ERAS rehabilitation. POD1: NG tube removed, oral fluids 0,5 L; I/V fluids 1 mL/Kg/h; POD2: stop I/V fluids if drinks >2000 mL; oral diet initiated from water to carbohydrate drink to enteral nutritional suspension, then to semifluids and normal food; POD 3–4: continue as above	Conventional PO. POD1: Parenteral nutrition until flatus; POD2: Parenteral nutrition until flatus; POD3-4: oral liquid started; POD 5–6: oral diet changed from liquids to semifluids and normal food	Ad undergoing elective laparoscopic radical gastrectomy for cancer	152 G1: 76 (3 not received intervention) G2: 76	Time to first defecation LOS Complications
Kang et al. (2018) Korea October 2012–August 2014	ERAS protocol, which provides for early feeding: Day of surgery: No NGT insertion; fluid restriction (1–2 L), Pod1: sips of water if tolerable; fluid restriction (1–2 L) Pod2: semifluid diet if tolerable; Fluid removal, Pod3: soft blended diet, if tolerable	Conventional: Day of surgery: No NGT insertion; Fluid (Dextrose 5% according to weight), Pod1: NPO; fluid (Dextrose 5% according to weight), Pod2: sips of water; Fluid (dextrose 5% according to body weight), Pod3: semifluid diet, Pod4: soft blended diet	Ad aged 20 to 80 years, undergoing totally laparoscopic distal gastrectomy for gastric cancer	97 G1:46 G2:51	NA LOS PO first flatus Complications
Geubbels et al. (2019), Netherlands January 2013–July 2014	ERAS protocol. After surgery: No nasogastric tubing Early oral feeding, restricted administration of fluids, Clear antiemetic protocol	Nasogastric tubing on the indication, No early oral feeding Conventional administration of fluids, No clear antiemetic protocol, at the discretion of the caring staff	Ad <76 years, BMI was 40 kg/m^2^ or above or 35 kg/m^2^, undergoing elective laparoscopic Roux-en-Y gastric bypass surgery	220 G1: 110 G2: 110 intention-to-treat	NA LOS Nausea/Vomiting Complications
Hwang et al. (2019) Korea March 2015–May 2017	ERAS Guidelines: Preventing PO nausea and vomiting with prokinetic agents; PO glycemic control; Early oral intake (PO artificial nutrition not routinely applied); Stimulation of bowel movement (oral laxatives and chewing gum); Artificial nutrition in the case of delayed gastric emptying.	Conventional: PO glycemic control; PO nasogastric intubation; Probably fasting	Ad <76 years of age undergoing elective open pancreaticoduodenectomy	276 G1:138 123 Analyzed G2: 138 124 Analyzed	NA LOS Complications
Li et al. (2019) China June 2014–June 2017	ERAS group: The ERAS group focused on the needs of the patients and avoided excessive fluid intake, mainly as oral water supplementation to prevent gastrointestinal edema	Conventional group: Glucose saline and amino acid were administered ev on the day of surgery, which was reasonably controlled according to the patient’s physiological requirements, intake, and output.	Cancer Ad aged 55 to 65 years undergoing elective laparoscopic colorectal surgery	200 G1:100 G2:100	Time to first defecation PO exhaust Complications
Wang et al. (2019) China March–October 2016	Attempt to drink warm water (B50 mL/h) 6 h after surgery. Routine prevention of nausea and vomiting for 2–3 days; POD1: Oral fluid intake 500 mL, ev fluid volume reduced, caloric intake 25–30 kcal/kg per day; NGT removed according to accepted criteria for extubation; oral lactulose for 2 days (in general) and stopped after passing of flatus; Chewing gum; POD2: Oral fluid intake 1,000 mL, liquid diet, ev fluids reduced; POD 3: Oral fluid intake 1,500 mL, liquid diet increased, ev fluid reduced; POD 4: frequent small amounts of oral fluids, small amounts of semi-liquid foods (porridge, noodles, or other soft foods), ev fluids stopped if possible, and oral intake increased; POD 5: frequent small amounts of oral fluids, gradual transition to total semi-liquid diet and soft foods; total intake maintained.	No water for 6 h before surgery. POD 1, out-of-bed activities were arranged according to the will of the patients. The number of ev fluids was not controlled, and oral fluids and food were permitted after flatus was passed.	Gastric cancer Ad <76 years of age undergoing elective radical gastrectomy	60 G1:30 G2:30	Time to first defecation LOS Time to first flatus Nausea/Vomiting Complications
Cao et al. (2021) China January 2014–December 2018	ERAS protocol, which provides for early feeding: Intraoperative: No NGT drainage; oral intake of a little clear water after-effects of anesthesia disappear; POD1: Start of clear liquid diet at dinner; POD3: Start of soft diet as tolerated	Intraoperative: Routine use of NGT drainage PO: Ev infusion of 2.0–3.0 L of Ringer lactate for 3 days; start to drink water if bowel sounds are heard; diet build-up from the day after flatus; three steps (clear liquid-full liquid-soft diet)	Ad aged 65 to 85 years, with primary gastric cancer, undergoing elective laparoscopy-assisted radical gastrectomy.	171 G1: 85 G2: 86	Time to first defecation Time to first flatus LOS

Ev, intravenous; Ad, adults; POD; Postoperative Day, ERAS; Enhanced Recovery After Surgery; LOS, length of postoperative stay NGT; Nasogastric tube, NPO; Nil per os.

### 3.2 Risk of bias and publication bias

Twenty-two studies resulted in an unclear risk of bias due to different reasons: 16 had an unclear randomization process, 13 had unclear information concerning the deviations from the intended interventions, 13 regarding the selection of the results, mainly due to a prespecified protocol not available, and nine regarding the measurement of the outcome (Supplementary File S3). Regarding the remaining articles, seven were judged at low risk of bias, while the other six were at high risk of bias. A high risk of bias was detected in three studies due to issues related to the outcome measurement; in one study, it was due to the randomization process, and in another study, it was due to outcome measurement process deviations from the intended protocol.

The funnel plot (Supplementary File S4) and Egger’s test revealed a strongly suspected publication bias in favor of the intervention for the outcomes “First passage of the stool,” “First flatus,” and LOS, but not for “Complications” and “Vomiting.”

### 3.3 Outcomes

We have reported the results according to the five outcomes investigated, which are the first passage of stool, the first passage of flatus, LOS, complications, and vomiting.

Furthermore, 17 trials (50%) reported the first passage of stool, 33 studies (94.3%) reported LOS and postoperative complications, 23 studies (65.7%) investigated the first passage of flatus, and 15 studies (47.1%) reported vomiting (Table 1). Meta-analyses were conducted, including 12 studies for the first passage of stool, 13 studies for the first flatus, 12 studies for LOS, 31 studies for complications, and 12 studies for vomiting (Table 2).

**Table 2:** Summary of findings Table.

Outcome № of participants (studies)	Relative effect (95% CI)	Anticipated absolute effects (95% CI)			Certainty	What happens
		Without early feeding	With early feeding	Difference		
First passage of stool N of participants: 2112 (12 RCTs)	–		–	MD **0.99 days lower** (1.25 lower to 0.72 lower)	⨁⨁⨁◯ Moderate^a,b^	Early feeding may result in a large reduction in the time of first passage of stool.
First Flatus N of participants: 2496 (13 RCTs)	–		–	MD **0.7 days lower** (0.87 lower to 0.53 lower)	⨁⨁⨁◯ Moderate^a,b^	Early feeding probably reduces the time to the first flatus.
LOS N of participants: 2421 (12 RCTs)	–		–	MD **1.54 days lower** (1.98 lower to 1.1 lower)	⨁⨁◯◯ Low^a,b,c^	Early feeding may result in a large reduction of the postoperative length of hospital stay
Complications N of participants: 4887 (33 RCTs)	**RR 0.69** (0.59 to 0.80)	23.5%	**16%** (13.9 to 18.8)	**7.3% fewer** (9,7 fewer to 4,7 fewer)	⨁⨁⨁◯ Moderate^d^	Early feeding probably reduces complications.
Vomiting N of participants: 2796 (12 RCTs)	**RR 0.89** (0.67 to 1.18)	10.3%	**9.1%** (6.9 to 12.1)	**1.1% fewer** (3,4 fewer to 1,8 more)	⨁⨁⨁◯ Moderate^e^	Early feeding likely does not reduce vomiting.

Patient or population: patients undergoing gastrointestinal surgery. Intervention: Early oral feeding. Comparison: Delayed oral feeding. ^*^The risk in the intervention group (and its 95% confidence interval) is based on the assumed risk in the comparison group and the relative effect of the intervention (and its 95% CI). CI, confidence interval; LOS, Length of postoperative hospital stay; MD, mean difference; N, number; RR, risk ratio. GRADE Working Group grades of evidence. High certainty: we are very confident that the true effect lies close to that of the estimate of the effect. Moderate certainty: we are moderately confident in the effect estimate: the true effect is likely to be close to the estimate of the effect, but there is a possibility that it is substantially different. Low certainty: our confidence in the effect estimate is limited: the true effect may be substantially different from the estimate of the effect. Very low certainty: we have very little confidence in the effect estimate: the true effect is likely to be substantially different from the estimate of effect. Explanations: ^a^Downgraded for inconsistency (Wide variance of point estimates across studies and considerable heterogeneity). ^b^Funnel plots and Egger’s Test suggest a publication bias in favor of the intervention. ^c^Downgraded for indirectness (Length of hospital stay was deemed to be influenced by several other interventions in the multimodal and ERAS program). ^d^Downgraded for indirectness (outcome including different complications: minor and major complications in all grades of Clavien-Dindo Classification). ^e^Downgraded for indirectness (included various episodes of vomiting, nausea and vomiting, vomiting and diarrhea).

#### 3.3.1 First passage of stool

We pooled data from 12 studies out of 17 investigating the first passage of stool, four of which were evaluated as interventions for early oral feeding, while eight were the ERAS program or other multimodal programs. With a moderate certainty of evidence (Table 2), early feeding, whether standalone or within a wider program, may reduce the time to first passage of stool compared to delayed feeding (2,112 patients; MD −0.99 days; CI95% −1.25, −0.72; I2 88%, Supplementary File S5).

Out of the five studies investigating multimodal (40, 48) or ERAS (63) interventions not pooled in the meta-analysis, three reported a statistically significant reduction in the time to first defecation in favor of the intervention (Supplementary File S6).

#### 3.3.2 First passage of flatus

Out of 22 studies (64.7%) evaluating the time to first passage of flatus, 13 provided useful data to be pooled in the meta-analysis. There is moderate certainty of evidence that early feeding, either alone or as part of a larger program, may reduce the time to the first flatus among the intervention group compared to delayed feeding (2,496 patients; MD −0.70 days; CI 95% −0.87, −0.53; I2 85%, Table 2; Supplementary File S7). Seven out of eight studies, which were not pooled in the meta-analysis, showed a statistically significant difference in the time to the first flatus in favor of the intervention (Supplementary File S6). Two of these studies involved early feeding (65, 68), while the remaining used the ERAS program (51, 57, 58, 63) or multimodal interventions (45, 48). One study investigating a multimodal intervention reported no differences between the groups (72).

#### 3.3.3 Los

All but two of the 34 included studies evaluated LOS. Pooled data from 16 studies with low certainty of evidence showed that early feeding, either alone or as part of a larger program, may lead to a reduced postoperative hospital stay (2,819 patients; MD −1.31 days; CI 95% −1.59, −1.03 days; I2 83%, Table 2; Supplementary File S8).

Sixteen studies reported median values, and in half of these studies, early feeding favored the intervention group with statistically significant results (Supplementary File S6), both as a single intervention (45, 65) and when embedded in a multimodal (40, 56) or ERAS program (51, 57, 58, 63).

#### 3.3.4 Complications

Thirty-three studies (94.2%) investigated the occurrence of postoperative complications and were pooled in the meta-analysis to assess the risk likelihood of incurring at least one complication. The most common complications were anastomotic leakage and wound infection (Supplementary File S9). With moderate certainty of the evidence, early feeding, either alone or as part of a larger program, may reduce the risk of incurring at least one complication by 31%, with the risk reduction ranging from 41 to 20% compared to delayed feeding (4,887 participants; RR 0.69; CI 95% 0.59, 0.80; I^2^ 34%, Supplementary File S10A).

#### 3.3.5 Vomiting

Vomiting was reported in 15 studies. Based on a meta-analysis of 13 studies, there is moderate certainty in the evidence that early feeding, either alone or as part of a larger program, has no overall effect on vomiting compared to delayed feeding (2,856 patients; RR 0.90; CI 95% 0.68, 1.18; I^2^ 32%, Supplementary File S10B).

### 3.4 Subgroup and sensitivity analyses

The subgroup analysis was performed for the first passage of the stool, the first flatus, and LOS due to the significant heterogeneity detected in the overall analysis. The subgroup analysis based the type of the intervention (early oral feeding vs. multimodal/ERAS interventions) revealed no differences in the time to the first passage of the stool (Supplementary Files S11A, S12 studies) and the first flatus (Supplementary Files S12A, S13 studies). However, multimodal or ERAS programs led to a greater reduction in LOS (*p* = 0.02, Supplementary Files S13A, S16 studies). However, heterogeneity remained high among subgroups for all the outcomes. The subgroup analysis based on the site of intervention (colon and rectal surgery vs. bowel/abdominal/gastric surgery) showed no differences in the outcomes (Supplementary Files S11B–S13B). However, a reduction from an overall considerable (I^2^ = 85%) to substantial (I^2^ = 52%) heterogeneity was observed among studies targeting colon and rectal surgery for the outcome “First flatus” (Supplementary File S12B).

All the sensitivity analyses confirmed the results obtained from the overall analysis (Supplementary Files S14–S18).

### 3.5 Effects on outcomes by the type of oral feeding

A meta-analysis according to the type of oral feeding or start of the oral diet was not possible due to the high heterogeneity among studies. However, a visual representation shows that there is no hypothetical association between the type of oral feeding on the first postoperative day and differences in outcomes (Supplementary File S19).

## 4 Discussion

### 4.1 Main findings

To the best of our knowledge, this is the first review assessing the effectiveness of early oral feeding, both alone and within a wider peri-operative program, on the recovery of intestinal motility among patients undergoing gastrointestinal surgery. In 34 trials, our findings suggest that early oral feeding may reduce the time to the first defecation and flatus while reducing the length of hospital stay (LOS) and postoperative complications without increasing the risk of vomiting. Studies support the safety of early oral feeding, whether standalone or within broader programs. The subgroup analysis indicates that the intervention type and the surgery site do not impact its effectiveness in reducing time to first defecation, flatus, and LOS. However, multimodal and ERAS programs show a greater LOS reduction compared to studies on early oral feeding alone, possibly due to additional components such as early mobilization and diverse pain management strategies.

The explanation for the considerable statistical heterogeneity among studies may have been missed due to differences in intervention components, which made it difficult to group the studies. For example, Sun et al. (56) investigated multiple oral feeding strategies, including appetite stimulation programs, drinking juice, enteral nutrition suspension, and the use of chewing gum, all of which demonstrated efficacy in reducing the likelihood of postoperative ileus (POI) (73). Relevant differences in nasogastric tube management were also detected; for example, in some studies, the tube was not positioned (52), removed according to accepted criteria for extubating (60), or removed within 12–24 h after surgery (38, 50). Different types of diet and start timing of fluids and solid food were detected, with or without parenteral nutrition (53) or oral supplements (56, 57). The administration of fluids alone (38) compared with a soft diet on the first postoperative day, as well as administering lactulose (60) or laxatives (61), could also affect the recovery of intestinal motility.

We could not categorize articles by feeding modality due to missing information, terminology variations, and differences in the timing and type of diets. Consequently, recommendations on the most beneficial early oral feeding type for investigated outcomes are unavailable. Despite diverse concepts and timings of “early oral feeding,” all effect estimates for the primary endpoint, first stool passage, consistently favor significance.

In addition, differences in the type of intervention and the underlying pathology could differently impact the incidence and length of POI, LOS, and complications and, therefore, increase the statistical heterogeneity among studies. However, analyzing the first stool passage as a proxy for postoperative ileus (POI), we conducted a subgroup analysis comparing colon-rectal interventions to bowel, abdominal, and gastric surgeries. No outcome differences were observed, affirming the reliability of the results.

Moreover, differences between countries might be relevant in terms of progress in surgery techniques and perioperative management protocols.

Despite the significant heterogeneity among the studies, results support the safe and beneficial transferability of early oral feeding in clinical practice. However, publication bias in favor of early feeding for the time to first defecation, first flatus, and LOS among studies included in the meta-analysis should be considered in interpreting results since it might result in overestimating effects. However, among the studies not included in the meta-analysis, two, one, and eight studies with no statistically significant results were found for the time to the first defecation, first stool, and LOS, respectively. This balances the publication bias, further mitigated by the negative rating assigned by applying the GRADE approach, thus reducing the quality of the evidence. According to the GRADE approach, the results are based on a moderate quality of evidence for the first passage of stool, the first flatus, complications, and vomiting, meaning that we are moderately confident that the true effect is likely to be close to the estimate of the effect. The evidence was rated low quality for the LOS, reducing our confidence in the effect estimate because the true effect may be substantially different.

### 4.2 Comparison with previous evidence

Our findings were mostly consistent with the evidence available on the time to first stool passage, LOS, and vomiting, while discordant results emerged on complications. We compared our findings with those of five recently published reviews on the effectiveness of early feeding in gastrointestinal surgery (28) (14 studies), digestive tract surgery (29) (11 studies), lower gastrointestinal surgery (22) (17 studies), and colorectal surgery [7 studies (30), plus 8 of only fluids (27)].

We found a similar reduction in the time to the first defecation (28) (−0.99 vs. −1 day) in the group receiving early nutritional support compared to those receiving delayed feeding (28).

According to previous reviews, early feeding reduces LOS (22, 28, 30); however, we found a smaller reduction of −1.31 days compared to −1.59 days (30), −1.95 days (22), and − 2.29 days (28).

By comparing the findings on complication prevention, discordant results emerged. Specifically, we found a significant reduction in the likelihood of incurring a postoperative complication, according to two previous reviews with almost similar statistically significant results [RR 0.70 compared to 0.70 (30), 0.72 (29), and 0.61 (28)]. In addition, our positive results on complications are further supported by the review of Shu and colleagues (29), which showed a reduction in infectious complication rates (RR 0.50, CI95% 0.38, 0.67). However, two reviews reported no effects on complication prevention by early feeding (22, 27), but Herbert and colleagues’ review was not entirely comparable with our results, as the authors analyzed the risk of mortality, anastomotic leakage, wound infection, abdominal abscess, and pneumonia separately. Regarding nausea and vomiting, this review found no evidence of the beneficial effect of early feeding, which is consistent with the previous literature (22, 27, 30).

Therefore, future research should focus on the effect of early oral feeding on LOS to confirm the consistency of our positive findings. Furthermore, despite the moderate confidence in the effect estimate, more studies are needed to investigate the effect of early feeding on several types of complications since we investigated the risk of at least one complication without specifying it.

Other suggestions for future research, gathered by comparing with previous research studies, encompass the need for a standardized definition of early oral feeding and the need to investigate the relationship between different modalities of early oral feeding with components of the multimodal program recommended by the ERAS guidelines (19). The lack of clarity on the type of food, start timing, and food consistency could threaten the reliability of the comparison between studies, and further efforts should be devoted to solving this issue by academics and clinicians. This could be helped by a more detailed reporting of interventions in the published studies. According to the ERAS guidelines, early oral feeding is considered safe in patients with a new non-diverted colorectal anastomosis, starting 4 h post-surgery. Furthermore, adopting a low residue diet and incorporating oral nutritional supplements might better improve outcomes (19). However, we were not able to confirm these results or provide further recommendations due to heterogeneity among studies. Therefore, we suggest additional research to determine the best type of early diet and its most effective combination with other perioperative interventions. Furthermore, differences in surgery sites and techniques should be further investigated as confounders of the effect of early oral feeding on POI and LOS.

### 4.3 Strengths and limitations

This review has some strengths and limitations. The inclusion of studies assessing the multimodal or ERAS program was considered both a weakness and a strength point. Specifically, our findings might have been biased by other interventions, including the use of opioids, vomiting prevention protocol, parenteral nutrition, and early mobilization. However, the subgroup analysis confirmed the benefit of early feeding alone and provided evidence for the effectiveness of multimodal and ERAS interventions in promoting recovery of intestinal motility and LOS. This subgroup analysis was possible since we included early oral feeding both as a single intervention or a component of complex interventions, which is different from previous reviews. Indeed, available reviews included studies only on oral feeding or multimodal interventions, with a range of 7 (26, 27, 30) to 17 (22) studies, while we gathered 34 studies.

Combining complications into a single outcome poses a limitation in assessing the postoperative risk, potentially yielding biased results due to variations in severity. Early feeding may not be directly linked to many detected complications, and outcomes could be influenced by perioperative patient management in studies incorporating multimodal or ERAS programs. If statistically significant results favored the intervention, confidence in establishing a direct association between early feeding and mortality, bleeding, anastomotic leakage, and infections would be uncertain. Vomiting was the only directly associable complication, and we performed a separate analysis for it.

Additionally, our study did not specify a publication time frame, encompassing studies from 1995 to 2021. This lack of temporal specificity could have introduced potential influences from advancements in surgical techniques and LOS reduction. Nevertheless, upon scrutinizing the extracted data, we found no linear improvement in LOS or other outcomes based on the publication year or the study’s country.

Finally, there was substantial heterogeneity in the surgeries included in terms of (i) the type (upper and lower gastrointestinal surgeries and hepatobiliopancreatic procedures); (ii) the underlying disease (benign diseases and malignant tumors); (iii) the complexity (laparoscopic cholecystectomy and some bariatric surgeries have a lower risk of POI, compared to pancreaticoduodenectomy or colorectal surgeries); and (iv) the surgical approach (both open and minimally invasive surgical approaches were gathered). This merger reasonably may have impacted the results. However, we still consider that the results are reliable and generalizable.

## 5 Conclusion

Our study supports the practice of postoperative early oral feeding as a standalone intervention or within a multi-component program, including the ERAS protocol, after gastrointestinal surgery, especially referring to colorectal, bowel, abdominal, and gastric surgeries. We showed that postoperative early oral feeding may shorten the time of the first passage of the stool by 1 day on average, thereby reducing POI by fastening intestinal mobility. This could help to improve the nutritional status and autonomy recovery and prevent complications and prolonged LOS (6, 9–11). Indeed, our results support moderate confidence to a 30% reduction in the risk complications and a decrease of 1.3 days in LOS, even though the effect on LOS is of lower confidence.

## Data availability statement

The original contributions presented in the study are included in the article/Supplementary material, further inquiries can be directed to the corresponding author.

## Author contributions

FC: Conceptualization, Methodology, Project administration, Writing – review & editing. JL: Formal analysis, Investigation, Visualization, Writing – original draft. AC: Investigation, Methodology, Writing – review & editing. MC: Conceptualization, Methodology, Writing – review & editing. EM: Investigation, Methodology, Writing – review & editing. SP: Conceptualization, Methodology, Writing – review & editing. EA: Data curation, Investigation, Methodology, Project administration, Writing – review & editing.
