# Supplementary material for: The effect of early oral postoperative feeding on the recovery of intestinal motility after gastrointestinal surgery: a systematic review and meta-analysis of randomized clinical trials
Source: Front Nutr. 2024 May 16;11:1369141. doi: 10.3389/fnut.2024.1369141 (PMC11137291; doi:10.3389/fnut.2024.1369141)
Supplement: Supplementary file 1 [file Data_Sheet_1.pdf]

Supplementary Files

Supplementary File 1. Search string 2
Supplementary File 2. List of excluded studies 3
Supplementary File 3. Risk of bias of the included studies 6
Supplementary File 4. Funnel plots 7
Supplementary File 5. Forest plot for the first passage of the stool 8
Supplementary File 6. Outcomes reported as median and interquartile range not pooled in the meta-analysis 9
Supplementary File 7. Forest plot for the first passage of flatus 10
Supplementary File 8. Forest plot for the length of post-operative hospitalization (LOS) 11
Supplementary File 9. Complications evaluated in the included articles 12
Supplementary File 10. Forest Plot for Complications 16
Supplementary File 11. Subgroup analysis for the outcome “First passage of the stool” 17
Supplementary File 12. Subgroup analysis for the outcome “First flatus” 18
Supplementary File 13. Subgroup analysis for the outcome “Length of hospitalization” 19
Supplementary File 14. Sensitivity analysis for the outcome “First passage of the stool” 20
Supplementary File 15. Sensitivity analysis for the outcome “First passage of the flatus” 21
Supplementary File 16. Sensitivity analysis for the outcome “Length of Hospitalization” 22
Supplementary File 17. Sensitivity analysis for the outcome “Complications” 23
Supplementary File 18. Sensitivity analysis for the outcome “Vomiting” 24
Supplementary file 19. Differences in outcomes by type of diet in the first postoperative day 25

Supplementary File 1. Search string

**Population**	("upper abdominal surgery" OR "abdominal surgery" OR "gastrointestinal surgery" OR "surgical procedure" OR "reconstructive surgical procedures" OR "digestive system surgical procedure" OR "digestive system" OR "upper gastrointestinal tract" OR "lower gastrointestinal tract" OR "resection anastomosis"[Title/Abstract] OR "gastrointestinal tract" OR gastric OR stomach OR epigastric OR gastrectomy OR "Billroth I" OR "Billroth II" OR Roux-en-Y OR "partial gastrectomy" OR "total gastrectomy" OR "distal gastrectomy" OR gastropexy OR oesophagus OR esophagus OR esophageal OR oesophageal OR esophagectomy OR oesophagectomy OR esophagostomy OR oesophagostomy OR esophagotomy OR oesophagotomy OR esophagoplasty OR oesophagoplasty OR "esophagogastric junction" OR "oesophagogastric junction" OR "esophago-gastric junction" OR "oesophago-gastric junction" OR cardias OR "heller myotomy" OR pylorus OR pyloric OR pyloromyotomy OR pancreas OR pancreatic OR pancreatectomy OR pancreaticoduodenectomy OR duodenopancreatectomy OR pancreatoduodenectomy OR pancreaticojejunostomy OR pancreatojejunostomy OR biliary OR "bile duct" OR liver OR hepatic OR hepato OR hepato-biliary OR biliopancreatic OR cholecist OR gallbladder OR "gall bladder" OR cholecystectomy OR cholecystostomy OR choledochostomy OR hepatectomy OR intestine OR intestinal OR colonic OR gut OR bowel OR ileum OR ileo OR "anal canal" OR cecum OR colon OR rectum OR rectal OR colorectal OR sigmoid OR sigmoidal OR jejunum OR colectomy OR sigmoidectomy OR proctocolectomy OR proctectomy OR sphincterotomy OR diverticulectomy)
**Intervention**	("early oral intake"[Title/Abstract] OR "early oral feeding"[Title/Abstract] OR "early enteral feeding"[Title/Abstract] OR "feeding methods"[Title/Abstract] OR eating OR nutrition OR "nutrition therapy"[Title/Abstract] OR intake OR "late oral feeding"[Title/Abstract] OR "early feeding"[Title/Abstract] OR "early nutrition"[Title/Abstract] OR "enteral nutrition"[Title/Abstract] OR diet OR fasting OR "postoperative nutrition"[Title/Abstract] OR "sip feeding"[Title/Abstract] OR "tube feeding"[Title/Abstract] OR nutritional OR "early enteral nutrition"[Title/Abstract] OR "dietary intake"[Title/Abstract] OR food OR liquid OR "clear liquid diet"[Title/Abstract] OR "typical diet"[Title/Abstract] OR Sip OR "oral intake"[Title/Abstract] OR "semi-solid"[Title/Abstract] OR semisolid OR semiliquid OR "semi-liquid"[Title/Abstract] OR "nil by mouth"[Title/Abstract] OR "food avoidance"[Title/Abstract] OR "soft diet"[Title/Abstract] OR "regular diet"[Title/Abstract] OR water OR "late enteral feeding" OR "late feeding" OR "late nutrition" OR "late enteral nutrition" OR "enhanced recovery after surgery")
**Outcomes**	(motility OR dysmotility OR nausea OR cramp OR cramping OR pain OR ache OR "gastrointestinal distress"[Title/Abstract] OR spasm OR flatus OR "epigastric pain"[Title/Abstract] OR regurgitation OR "feeding tolerance" OR "oral tolerance" OR "postoperative ileus"[Title/Abstract] OR "gut motility"[Title/Abstract] OR "gut dysmotility" OR "bowel dysfunction"[Title/Abstract] OR "bowel function" OR "intestinal dysfunction"[Title/Abstract] OR "intestinal function" OR "gastrointestinal motility"[Title/Abstract] OR "gastrointestinal dysmotility"[Title/Abstract] OR "gastrointestinal symptoms"[Title/Abstract] OR "gastrointestinal function"[Title/Abstract] OR "gastrointestinal dysfunction"[Title/Abstract] OR "gastrointestinal distress"[Title/Abstract] OR "gastrointestinal disturbance"[Title/Abstract] OR "gastrointestinal adverse events"[Title/Abstract] OR "gastrointestinal transit"[Title/Abstract] OR "gastrointestinal disorders"[Title/Abstract] OR "abdominal discomfort"[Title/Abstract] OR "abdominal comfort"[Title/Abstract] OR "abdominal pain"[Title/Abstract] OR "Abdominal distention"[Title/Abstract] OR "abdominal dysfunction"[Title/Abstract] OR "gastric motility"[Title/Abstract] OR "gastric dysmotility" OR constipation OR defecation OR dyspepsia OR stool OR vomiting OR "paralytic ileus"[Title/Abstract] OR ileus OR bloating OR "bowel function" OR "intestinal function")

Note. The search string was adapted in the other databases according to search rules.

Supplementary File 2. List of excluded studies

Excluded for wrong intervention

1. Schmidt M, Eckardt R, Scholtz K, et al. Patient Empowerment Improved Perioperative Quality of Care in Cancer Patients Aged ≥ 65 Years - A Randomized Controlled Trial. PLoS One. 2015;10(9):e0137824. doi:10.1371/journal.pone.0137824
2. Kim TH, Lee YJ, Bae K, et al. The investigation of diet recovery after distal gastrectomy. Medicine (Baltimore). Oct 2019;98(41):e17543. doi:10.1097/md.0000000000017543
3. Li B, Liu HY, Guo SH, Sun P, Gong FM, Jia BQ. Impact of early postoperative enteral nutrition on clinical outcomes in patients with gastric cancer. Genet Mol Res. Jun 29 2015;14(2):7136-41. doi:10.4238/2015.June.29.7
4. Huang L, Li G, Zhou B, Wei W, Chen H, Wei Q. Clinical effects of total protein and short peptide enteral nutrition during recovery after radical gastrectomy. Asia Pac J Clin Nutr. 2020;29(2):239-244. doi:10.6133/apjcn.202007_29(2).0005
5. Yang P, Long WJ, Wei L. Chewing Xylitol Gum could Accelerate Bowel motility Recovery after Elective Open Proctectomy for Rectal Cancer. Rev Invest Clin. 2018;70(1):53-58. doi:10.24875/ric.18002428
6. de Leede EM, van Leersum NJ, Kroon HM, van Weel V, van der Sijp JRM, Bonsing BA. Multicentre randomized clinical trial of the effect of chewing gum after abdominal surgery. Br J Surg. Jun 2018;105(7):820-828. doi:10.1002/bjs.10828
7. Pattamatta M, Smeets BJJ, Evers S, Rutten HJT, Luyer MDP, Hiligsmann M. Health-related quality of life and cost-effectiveness analysis of gum chewing in patients undergoing colorectal surgery: results of a randomized controlled trial. Acta Chir Belg. Oct 2018;118(5):299-306. doi:10.1080/00015458.2018.1432742
8. Byrne CM, Zahid A, Young JM, Solomon MJ, Young CJ. Gum chewing aids bowel function return and analgesic requirements after bowel surgery: a randomized controlled trial. Colorectal Dis. May 2018;20(5):438-448. doi:10.1111/codi.13930
9. Shum NF, Choi HK, Mak JC, Foo DC, Li WC, Law WL. Randomized clinical trial of chewing gum after laparoscopic colorectal resection. Br J Surg. Oct 2016;103(11):1447-52. doi:10.1002/bjs.10277
10. Topcu SY, Oztekin SD. Effect of gum chewing on reducing postoperative ileus and recovery after colorectal surgery: A randomised controlled trial. Complement Ther Clin Pract. May 2016;23:21-5. doi:10.1016/j.ctcp.2016.02.001
11. Atkinson C, Penfold CM, Ness AR, et al. Randomized clinical trial of postoperative chewing gum versus standard care after colorectal resection. Br J Surg. Jul 2016;103(8):962-70. doi:10.1002/bjs.10194
12. Gong Y, Zhang Q, Qiao L, et al. Xylitol Gum Chewing to Achieve Early Postoperative Restoration of Bowel Motility After Laparoscopic Surgery. Surg Laparosc Endosc Percutan Tech. Aug 2015;25(4):303-6. doi:10.1097/sle.0000000000000174
13. Andersson T, Bjerså K, Falk K, Olsén MF. Effects of chewing gum against postoperative ileus after pancreaticoduodenectomy--a randomized controlled trial. BMC Res Notes. Feb 10 2015;8:37. doi:10.1186/s13104-015-0996-0
14. van den Heijkant TC, Costes LM, van der Lee DG, et al. Randomized clinical trial of the effect of gum chewing on postoperative ileus and inflammation in colorectal surgery. Br J Surg. Feb 2015;102(3):202-11. doi:10.1002/bjs.9691
15. Tazegül Pekin A, Kerimoğlu OS, Doğan NU, et al. Gum chewing reduces the time to first defaecation after pelvic surgery: A randomised controlled study. J Obstet Gynaecol. 2015;35(5):494-8. doi:10.3109/01443615.2014.970146
16. Wronski S. Chew on this: reducing postoperative ileus with chewing gum. Nursing. Aug 2014;44(8):19-23. doi:10.1097/01.NURSE.0000451535.63211.a8
17. Forrester DA, Doyle-Munoz J, McTigue T, D'Andrea S, Natale-Ryan A. The efficacy of gum chewing in reducing postoperative ileus: a multisite randomized controlled trial. J Wound Ostomy Continence Nurs. May-Jun 2014;41(3):227-32. doi:10.1097/won.0000000000000019
18. Lim P, Morris OJ, Nolan G, Moore S, Draganic B, Smith SR. Sham feeding with chewing gum after elective colorectal resectional surgery: a randomized clinical trial. Ann Surg. Jun 2013;257(6):1016-24. doi:10.1097/SLA.0b013e318286504a
19. Zaghiyan K, Felder S, Ovsepyan G, et al. A prospective randomized controlled trial of sugared chewing gum on gastrointestinal recovery after major colorectal surgery in patients managed with early enteral feeding. Dis Colon Rectum. Mar 2013;56(3):328-35. doi:10.1097/DCR.0b013e31827e4971
20. Kobayashi T, Masaki T, Kogawa K, Matsuoka H, Sugiyama M. Efficacy of Gum Chewing on Bowel Movement After Open Colectomy for Left-Sided Colorectal Cancer: A Randomized Clinical Trial. Dis Colon Rectum. Nov 2015;58(11):1058-63. doi:10.1097/dcr.0000000000000452

21. You XM, Mo XS, Ma L, et al. Randomized Clinical Trial Comparing Efficacy of Simo Decoction and Acupuncture or Chewing Gum Alone on Postoperative Ileus in Patients With Hepatocellular Carcinoma After Hepatectomy. Medicine (Baltimore). Nov 2015;94(45):e1968. doi:10.1097/md.0000000000001968
22. Yang Y, Zuo HQ, Li Z, et al. Comparison of efficacy of simo decoction and acupuncture or chewing gum alone on postoperative ileus in colorectal cancer resection: a randomized trial. Sci Rep. Jan 19 2017;7:37826. doi:10.1038/srep37826
23. Müller PC, Ruzza C, Kuemmerli C, et al. 4/5 Gastrectomy in Patients Undergoing Pancreaticoduodenectomy Reduces Delayed Gastric Emptying. J Surg Res. May 2020;249:180-185. doi:10.1016/j.jss.2019.12.028
24. van Barneveld KW, Smeets BJ, Heesakkers FF, et al. Beneficial Effects of Early Enteral Nutrition After Major Rectal Surgery: A Possible Role for Conditionally Essential Amino Acids? Results of a Randomized Clinical Trial. Crit Care Med. Jun 2016;44(6):e353-61. doi:10.1097/ccm.0000000000001640
25. Cvetkovic A, Kalezic N, Milicic B, et al. The impact of different infusion solutions on postoperative recovery following colorectal surgery. J buon. Sep-Oct 2018;23(5):1369-1379.
26. Garulli G, Lucchi A, Berti P, Gabbianelli C, Siani LM. "Ultra" E.R.A.S. in laparoscopic colectomy for cancer: discharge after the first flatus? A prospective, randomized trial. Surg Endosc. Apr 2017;31(4):1806-1813. doi:10.1007/s00464-016-5177-2
27. Carrier G, Cotte E, Beyer-Berjot L, Faucheron JL, Joris J, Slim K. Post-discharge follow-up using text messaging within an enhanced recovery program after colorectal surgery. J Visc Surg. Aug 2016;153(4):249-52. doi:10.1016/j.jviscsurg.2016.05.016
28. Parekh D, Dancer RCA, Scott A, et al. Vitamin D to Prevent Lung Injury Following Esophagectomy-A Randomized, Placebo-Controlled Trial. Crit Care Med. Dec 2018;46(12):e1128-e1135. doi:10.1097/ccm.0000000000003405
29. Chen B, He Y, Xiao Y, et al. Heated fennel therapy promotes the recovery of gastrointestinal function in patients after complex abdominal surgery: A single-center prospective randomized controlled trial in China. Surgery. Nov 2020;168(5):793-799. doi:10.1016/j.surg.2020.05.040
30. Maruyama M, Goshi S, Kashima Y, Mizuhara A, Higashiguchi T. Clinical Effects of a Pectin-Containing Oligomeric Formula in Tube Feeding Patients: A Multicenter Randomized Clinical Trial. Nutr Clin Pract. Jun 2020;35(3):464-470. doi:10.1002/ncp.10392
31. Xiao-Bo Y, Qiang L, Xiong Q, et al. Efficacy of early postoperative enteral nutrition in supporting patients after esophagectomy. Minerva Chir. Feb 2014;69(1):37-46.
32. Joos AK, Palma P, Jonescheit JO, Hasenberg T, Herold A. Enteral vs parenteral nutrition in reconstructive anal surgery--a prospective-randomized trial. Colorectal Dis. Jul 2008;10(6):605-9. doi:10.1111/j.1463-1318.2007.01403.x
33. Bednarski BK, Nickerson TP, You YN, et al. Randomized clinical trial of accelerated enhanced recovery after minimally invasive colorectal cancer surgery (RecoverMI trial). Br J Surg. Sep 2019;106(10):1311-1318. doi:10.1002/bjs.11223
34. Sala P, Belarmino G, Machado NM, et al. The SURMetaGIT study: Design and rationale for a prospective pan-omics examination of the gastrointestinal response to Roux-en-Y gastric bypass surgery. J Int Med Res. Dec 2016;44(6):1359-1375. doi:10.1177/0300060516667862
35. Liu Y, Song X, Zhang Y, Zhou L, Ni R. The effects of comprehensive mental intervention on the recovery time of patients with postsurgical gastroparesis syndrome. J Clin Nurs. Nov 2014;23(21-22):3138-47. doi:10.1111/jocn.12554
36. Ulusar UD. Recovery of gastrointestinal tract motility detection using Naive Bayesian and minimum statistics. Comput Biol Med. Aug 2014;51:223-8. doi:10.1016/j.compbiomed.2014.05.013
37. Yao D, Zheng L, Wang J, Guo M, Yin J, Li Y. Perioperative Alanyl-Glutamine-Supplemented Parenteral Nutrition in Chronic Radiation Enteritis Patients With Surgical Intestinal Obstruction: A Prospective, Randomized, Controlled Study. Nutr Clin Pract. Apr 2016;31(2):250-6. doi:10.1177/0884533615591601
38. Uhlig C, Rössel T, Denz A, Seifert S, Koch T, Heller AR. Effects of a metabolic optimized fast track concept (MOFA) on bowel function and recovery after surgery in patients undergoing elective colon or liver resection: a randomized controlled trial. BMC Anesthesiol. Aug 17 2019;19(1):156. doi:10.1186/s12871-019-0823-6
39. Yaegashi M, Otsuka K, Itabashi T, et al. Daikenchuto stimulates colonic motility after laparoscopic-assisted colectomy. Hepatogastroenterology. Jan-Feb 2014;61(129):85-9.
40. Nagata S, Fukuzawa K, Iwashita Y, et al. Comparison of enteral nutrition with combined enteral and parenteral nutrition in post-pancreaticoduodenectomy patients: a pilot study. Nutr J. Jun 11 2009;8:24. doi:10.1186/1475-2891-8-24
41. Woodcock NP, Zeigler D, Palmer MD, Buckley P, Mitchell CJ, MacFie J. Enteral versus parenteral nutrition: a pragmatic study. Nutrition. Jan 2001;17(1):1-12. doi:10.1016/s0899-9007(00)00576-1
42. Shi Y, Zhang XP, Qin H, Yu YJ. Naso-intestinal tube is more effective in treating postoperative ileus than naso-gastric tube in elderly colorectal cancer patients. Int J Colorectal Dis. Jul 2017;32(7):1047-1050. doi:10.1007/s00384-017-2760-5

43. Liu L, Lv N, Hou C. Effects of a multifaceted individualized pneumoperitoneum strategy in elderly patients undergoing laparoscopic colorectal surgery: A retrospective study. Medicine (Baltimore). Apr 2019;98(14):e15112. doi:10.1097/md.0000000000015112
44. Trautvetter U, Camarinha-Silva A, Jahreis G, Lorkowski S, Glei M. High phosphorus intake and gut-related parameters - results of a randomized placebo-controlled human intervention study. Nutr J. Feb 16 2018;17(1):23. doi:10.1186/s12937-018-0331-4
45. Hamamsy ME, Bondok R, Shaheen S, Eladly GH. Safety and efficacy of adding intravenous N-acetylcysteine to parenteral L-alanyl-L-glutamine in hospitalized patients undergoing surgery of the colon: a randomized controlled trial. Ann Saudi Med. Jul-Aug 2019;39(4):251-257. doi:10.5144/0256-4947.2019.251
46. Ludwig K, Enker WE, Delaney CP, et al. Gastrointestinal tract recovery in patients undergoing bowel resection: results of a randomized trial of alvimopan and placebo with a standardized accelerated postoperative care pathway. Arch Surg. Nov 2008;143(11):1098-105. doi:10.1001/archsurg.143.11.1098
47. Narita K, Tsunoda A, Takenaka K, Watanabe M, Nakao K, Kusano M. Effect of mosapride on recovery of intestinal motility after hand-assisted laparoscopic colectomy for carcinoma. Dis Colon Rectum. Nov 2008;51(11):1692-5. doi:10.1007/s10350-008-9407-0
48. Brandl B, Lee YM, Dunkel A, Hofmann T, Hauner H, Skurk T. Effects of Extrinsic Wheat Fiber Supplementation on Fecal Weight; A Randomized Controlled Trial. Nutrients. Jan 22 2020;12(2)doi:10.3390/nu12020298
49. Li B, Liu HY, Guo SH, Sun P, Gong FM, Jia BQ. The postoperative clinical outcomes and safety of early enteral nutrition in operated gastric cancer patients. J buon. Mar-Apr 2015;20(2):468-72.
50. Karlsson A, Wendel K, Polits S, Gislason H, Hedenbro JL. Preoperative Nutrition and Postoperative Discomfort in an ERAS Setting: A Randomized Study in Gastric Bypass Surgery. Obes Surg. Apr 2016;26(4):743-8. doi:10.1007/s11695-015-1848-7

Excluded for wrong population

1. Kraus K, Fanning J. Prospective trial of early feeding and bowel stimulation after radical hysterectomy. Am J Obstet Gynecol. May 2000;182(5):996-8. doi:10.1016/s0002-9378(00)70134-7
2. Pearl ML, Frandina M, Mahler L, Valea FA, DiSilvestro PA, Chalas E. A randomized controlled trial of a regular diet as the first meal in gynecologic oncology patients undergoing intraabdominal surgery. Obstet Gynecol. Aug 2002;100(2):230-4. doi:10.1016/s0029-7844(02)02067-7
3. Peng AW, Juraschek SP, Appel LJ, Miller ER, 3rd, Mueller NT. Effects of the DASH Diet and Sodium Intake on Bloating: Results From the DASH-Sodium Trial. Am J Gastroenterol. Jul 2019;114(7):1109-1115. doi:10.14309/ajg.0000000000000283
4. Schilder JM, Hurteau JA, Look KY, et al. A prospective controlled trial of early postoperative oral intake following major abdominal gynecologic surgery. Gynecol Oncol. Dec 1997;67(3):235-40. doi:10.1006/gyno.1997.4860
5. The FO, Buist MR, Lei A, et al. The role of mast cell stabilization in treatment of postoperative ileus: a pilot study. Am J Gastroenterol. Sep 2009;104(9):2257-66. doi:10.1038/ajg.2009.268

Excluded for wrong study design

1. Zang YF, Li FZ, Ji ZP, Ding YL. Application value of enhanced recovery after surgery for total laparoscopic uncut Roux-en-Y gastrojejunostomy after distal gastrectomy. World J Gastroenterol. 2018;24(4):504-510. doi:10.3748/wjg.v24.i4.504
2. Zargar-Shoshtari K, Paddison JS, Booth RJ, Hill AG. A prospective study on the influence of a fast-track program on postoperative fatigue and functional recovery after major colonic surgery. J Surg Res. 2009;154(2):330-5. doi:10.1016/j.jss.2008.06.023

Supplementary File 3. Risk of bias of the included studies

3.A Summary Plot

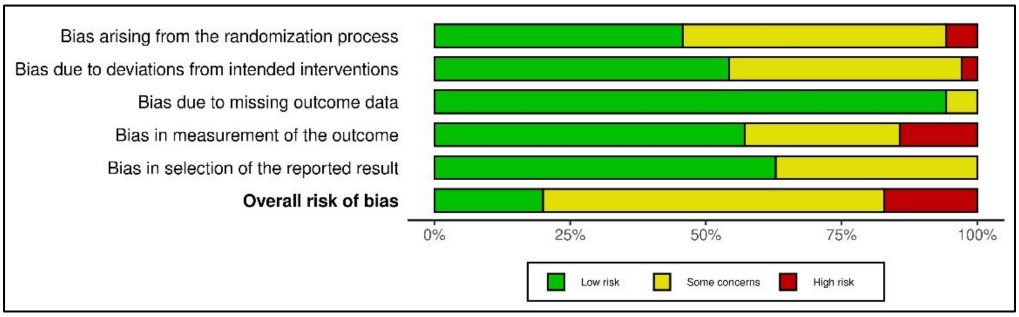

3.B Traffic Light Plot

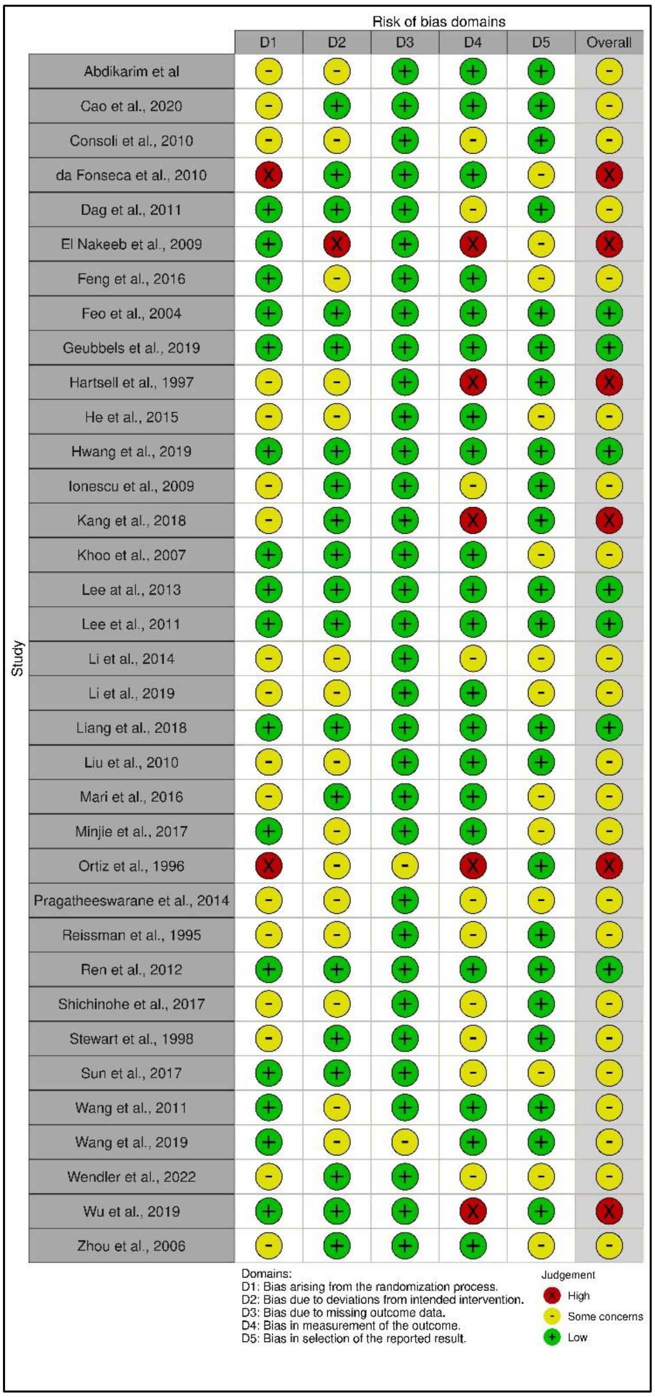

Supplementary File 4. Funnel plots

4.A First passage of the stool

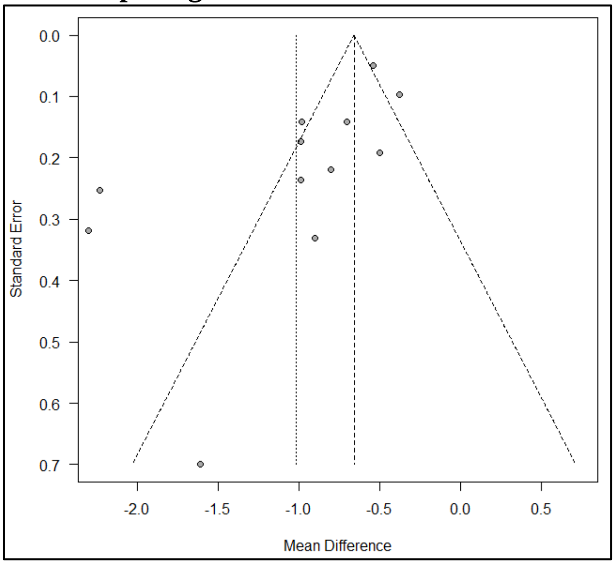

Note: Egger's test: bias = -3.19, SE= 1.07, p = .014

4.B First Flatus

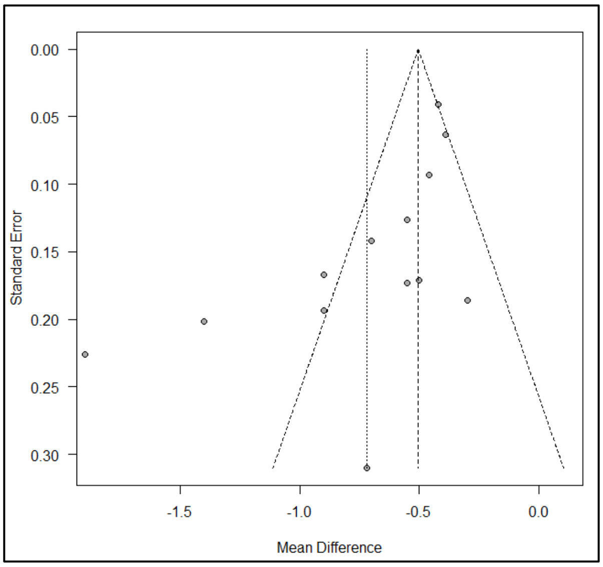

Note: Egger's test: bias = -2.93, SE= 0.97, p = .018

4.C Length of Hospitalization

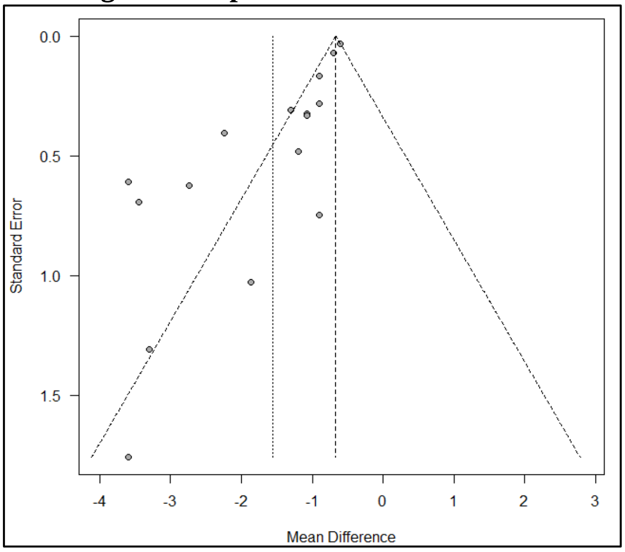

Note: Egger's test: bias = -2.41, SE= 0.38, p < .0001

4.D Complications

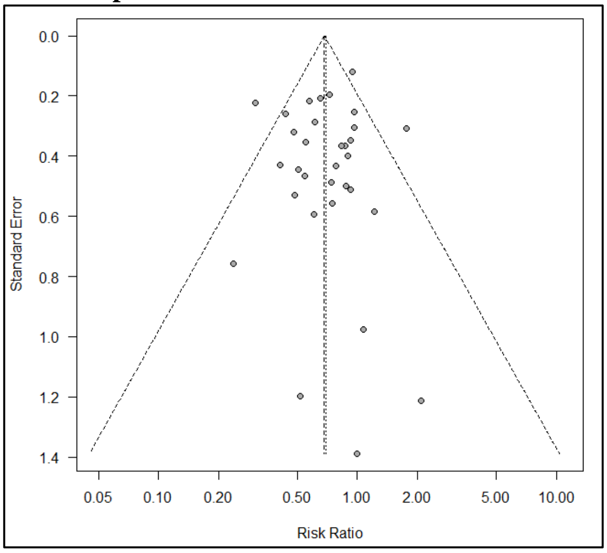

4.E Vomiting

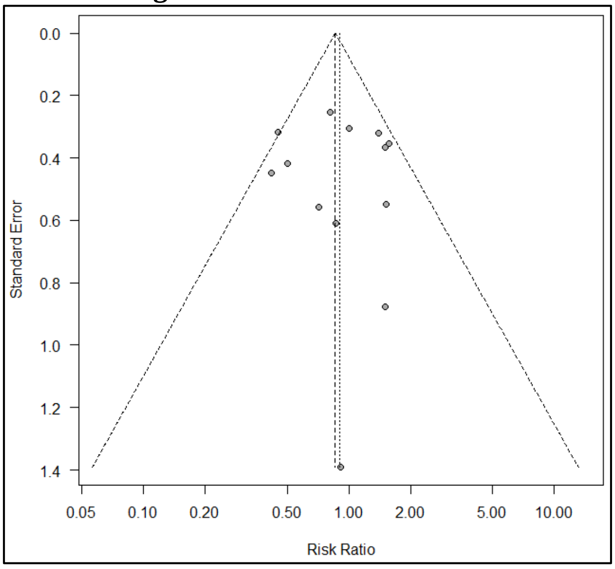

Supplementary File 5. Forest plot for the first passage of the stool

Study or Subgroup	Early feeding Mean	Early feeding SD	Early feeding Total	Delayed feeding Mean	Delayed feeding SD	Delayed feeding Total	Weight	Mean Difference IV, Random, 95% CI
Pragatheeswarane et al., 2014 (Bowel surgery)	3.8	1.3	60	6.1	2.1	60	6.8%	-2.30 [-2.92, -1.68]
Mingjie et al.,2017 (LP radical gastrectomy)	2.97	1.23	73	5.2	1.81	76	7.9%	-2.23 [-2.73, -1.73]
Wang et al., 2019 (Radical gastrectomy)	4.25	3.02	30	5.86	2.36	30	2.8%	-1.61 [-2.98, -0.24]
Li et al., 2014 (Colorectal surgery)	4.84	1.59	208	5.83	2.05	237	9.3%	-0.99 [-1.33, -0.65]
Feng et al., 2016 (Colorectal surgery)	4.84	1.59	121	5.83	2.05	120	8.2%	-0.99 [-1.45, -0.53]
Dag et al., 2011 (Colorectal surgery)	3.4	0.77	99	4.38	1.18	100	9.8%	-0.98 [-1.26, -0.70]
Shichinohe et al., 2017 (LP Colorectal surgery)	2.2	1.6	45	3.1	1.6	49	6.6%	-0.90 [-1.55, -0.25]
El Nakeeb et al., 2009 (Open colonic anast)	4.1	1.2	60	4.9	1.2	60	8.5%	-0.80 [-1.23, -0.37]
Zhou et al., 2006 (Colorectal res+Anast)	4.1	1.1	161	4.8	1.4	155	9.8%	-0.70 [-0.98, -0.42]
Sun et al., 2017 (Major abdominal surgery)	2.04	0.29	53	2.58	0.21	54	10.9%	-0.54 [-0.64, -0.44]
Abdikarim et al., 2015 (LP radical gastrectomy)	3.1	0.7	30	3.6	0.8	31	9.0%	-0.50 [-0.88, -0.12]
Li et al., 2019 (LP Colorectal surgery)	2.51	0.72	100	2.89	0.65	100	10.4%	-0.38 [-0.57, -0.19]
**Total (95% CI)**			**1040**			**1072**	**100.0%**	**-0.99 [-1.25, -0.72]**

Heterogeneity: $Tau^2 = 0.16$; $Chi^2 = 93.68$, df = 11 ($P < 0.00001$); $I^2 = 88\%$
Test for overall effect: Z = 7.34 ($P < 0.00001$)

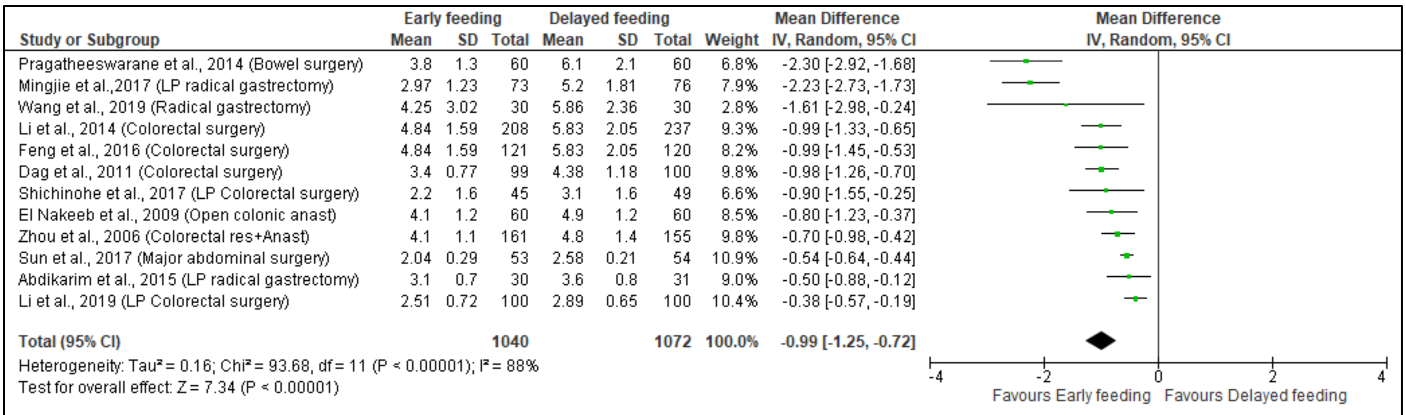

Note. LP; laparoscopic; Res, Resection; Anast, Anastomosis

Supplementary File 6. Outcomes reported as median and interquartile range not pooled in the meta-analysis

Author	Type of intervention	Intervention Group	Control group	
		**Median (IQR)**	**Median (IQR)**	**p-value**
**First passage of the stool**				
**Days**				
Khoo et al., 2007 (+ stoma functioning)	Multimodal	3 (1-5)	5 (0-23)	p <0.001
Feo et al., 2004	Multimodal	4 (2-8)	4 (2-7)	p > 0.05
Cao et al., 2020	ERAS	4 (2-6)	4.5 (2-7)	p=0.003
**Hours**				
Lee et al., 2013	Multimodal	65 (47 - 126)	98 (77 - 161)	p=0.015
Lee et al., 2011	Multimodal	113 (79 - 144)	120 (86 - 145)	p=0.485
**First passage of flatus**				
**Days**				
Stewart et al., 1998	Early feeding	3 (1-5)	4 (2-6)	p<0.01
Consoli et al., 2010	Early feeding	1 (NA)	2 (NA)	p<0.05
Cao et al., 2020	ERAS	2 (1-5)	3.5 (2-6)	p<0.001
He et al., 2015	ERAS	2 (1-4)	3 (2-5)	p=0.02
Kang et al., 2018 (mean, IQR)	ERAS	2.9 (2.25-3)	3.4 (3-4)	p = 0.004
**Hours**				
Lee et al., 2013	Multimodal	31.5 (22-49)	46 (31-66)	p=0.006
Lee et al., 2011	Multimodal	58 (39-74.2)	62 (41-79)	p=0.452
Wang et al., 2012	Multimodal	31 (26-40)	38 (32-51)	p<0.001
Liang et al., 2017	ERAS	38 (17-59)	64 (44-84)	p<0.001
**Incidence within 24 hours**				
Wendler et al., 2022		10 (25%)	5 (12.5%)	NSS
**Post-operative length of stay**				
Consoli et al., 2010	Early feeding	3 (NA)	5 (NA)	p<0.01
Wang et al., 2012	Early feeding	5.5 (5-6)	7 (6-8)	p<0.001
Lee et al., 2013	Early feeding	7.5 (7-11)	8 (7-10)	p=0.882
Stewart et al., 1998	Early feeding	9 (5-28)	11 (6-18)	p=0.10
Hwang et al., 2019	ERAS	11 (9-15)	11 (9.5-13)	p > 0.05
Liang et al., 2018	ERAS	5 (1-24)	8 (6-11)	p<0.001
Cao et al., 2020	ERAS	11 (7-11)	13 (8-20)	p<0.001
Kang et al., 2018	ERAS	5.4 (NA)	5.8 (NA)	p=0.038
Geubbels et al., 2019	ERAS	21.2 (15-95.3)	21.3 (6.2-143.3)	p=0.343
He et al., 2015	ERAS	6 (4-8)	10 (7-15)	p=0.04
Lee et al., 2011	Multimodal	7 (6-8)	8 (7-9)	p=0.065
Feo et al., 2004	Multimodal	7 (5-14)	7 (5-13)	p > 0.05
Khoo et al., 2007 (with readmissions)	Multimodal	5 (3-37)	7 (4-63)	p<0.001
Shichinohe eta al., 2017	Multimodal	9 (5-29)	10 (7-68)	p=0.176
Sun et al., 2017	Multimodal	8 (6-12)	10 (7-18)	p < 0.001

Note. NSS, Not statistically significant, p-value not reported

Supplementary File 7. Forest plot for the first passage of flatus

Study or Subgroup	Early feeding Mean	Early feeding SD	Early feeding Total	Delayed feeding Mean	Delayed feeding SD	Delayed feeding Total	Weight	Mean Difference IV, Random, 95% CI
Pragatheeswarane et al., 2014 (Bowel surgery)	2.6	0.9	60	4.5	1.5	60	6.1%	-1.90 [-2.34, -1.46]
Liu et al., 2010 (Gastrectomy surgery)	3.2	0.8	33	4.6	0.8	30	6.7%	-1.40 [-1.80, -1.00]
El Nakeeb et al., 2009 (Open colonic anast)	3.3	0.9	60	4.2	1.2	60	6.9%	-0.90 [-1.28, -0.52]
Mari et al., 2016 (LP Colorectal surgery)	1.5	0.6	40	2.4	0.9	43	7.6%	-0.90 [-1.23, -0.57]
Wang et al., 2019 (Radical gastrectomy)	2.63	1.07	30	3.35	1.32	30	4.5%	-0.72 [-1.33, -0.11]
Zhou et al., 2006 (Colorectal res+Anast)	4.1	1.1	161	4.8	1.4	155	8.2%	-0.70 [-0.98, -0.42]
Feng et al., 2016 (Colorectal surgery)	3.71	1.14	121	4.26	1.52	120	7.4%	-0.55 [-0.89, -0.21]
Li et al., 2014 (Colorectal surgery)	3.71	1.14	208	4.26	1.52	237	8.6%	-0.55 [-0.80, -0.30]
da Fonseca et al., 2011 (Colonic surgery)	1.5	0.5	24	2	0.7	26	7.4%	-0.50 [-0.84, -0.16]
Li et al., 2019 (LP Colorectal surgery)	2.35	0.52	100	2.8	0.77	100	9.4%	-0.45 [-0.63, -0.27]
Sun et al., 2017 (Major abdominal surgery)	1.37	0.22	53	1.79	0.2	54	10.3%	-0.42 [-0.50, -0.34]
Ren et al., 2012 (Colorectal radical res)	2.24	0.71	299	2.63	0.83	298	9.9%	-0.39 [-0.51, -0.27]
Shichinohe et al., 2017 (LP Colorectal surgery)	1.2	0.9	45	1.5	0.9	49	7.1%	-0.30 [-0.66, 0.06]
**Total (95% CI)**			**1234**			**1262**	**100.0%**	**-0.70 [-0.87, -0.53]**

Heterogeneity: Tau² = 0.07; Chi² = 79.39, df = 12 (P < 0.00001); I² = 85%
Test for overall effect: Z = 8.09 (P < 0.00001)

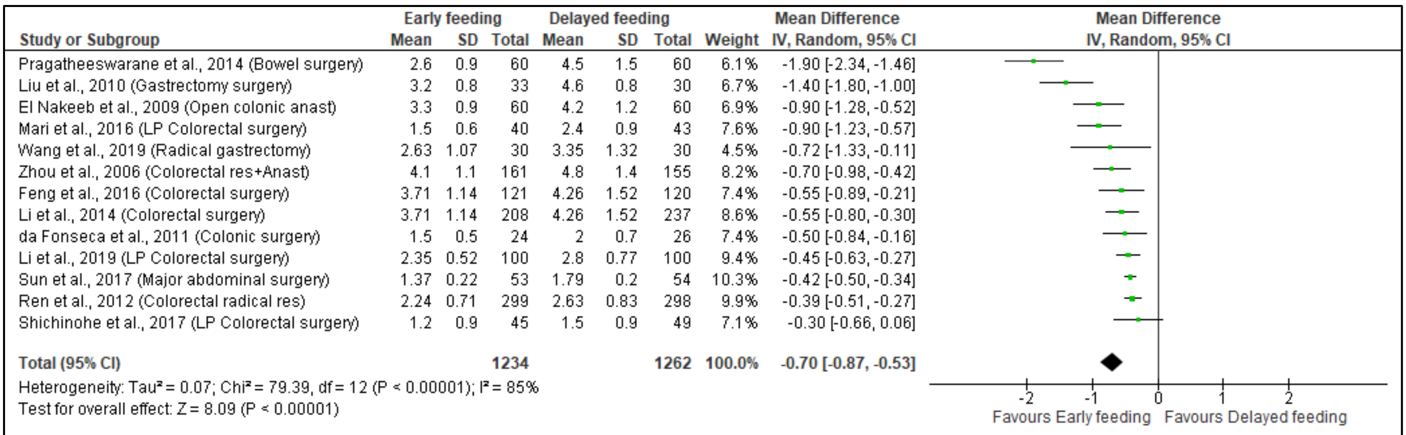

Note. LP; laparoscopic; Res, Resection; Anast, Anastomosis

Supplementary File 8. Forest plot for the length of post-operative hospitalization (LOS)

	Early feeding			Delayed feeding				Mean Difference
Study or Subgroup	Mean	SD	Total	Mean	SD	Total	Weight	IV, Random, 95% CI
Liu et al., 2010 (Gastrectomy surgery)	6.2	1.9	33	9.8	2.8	30	3.9%	-3.60 [-4.79, -2.41]
da Fonseca et al., 2011 (Colonic surgery)	4	3.7	24	7.6	8.1	26	0.6%	-3.60 [-7.05, -0.15]
Dag et al., 2011 (Colorectal surgery)	5.55	2.35	99	9	6.5	100	3.2%	-3.45 [-4.81, -2.09]
Pragatheeswarane et al., 2014 (Bowel surgery)	11.1	5.5	60	14.4	8.5	60	1.1%	-3.30 [-5.86, -0.74]
Ionescu et al., 2009 (Colorectal surgery)	6.43	3.41	48	9.16	2.67	48	3.7%	-2.73 [-3.96, -1.50]
Mingjie et al.,2017 (LP radical gastrectomy)	6.38	2.04	73	8.62	2.87	76	6.4%	-2.24 [-3.04, -1.44]
Wang et al., 2019 (Radical gastrectomy)	8.89	3.27	30	10.76	4.58	30	1.7%	-1.87 [-3.88, 0.14]
Mari et al., 2016 (LP Colorectal surgery)	4.7	1.2	40	6	1.6	43	8.3%	-1.30 [-1.91, -0.69]
Zhou et al., 2006 (Colorectal res+Anast)	8.4	3.4	161	9.6	5	155	5.3%	-1.20 [-2.15, -0.25]
Li et al., 2014 (Colorectal surgery)	8.54	3.18	208	9.62	3.83	237	7.8%	-1.08 [-1.73, -0.43]
Feng et al., 2016 (Colorectal surgery)	7.54	2.18	121	8.62	2.83	120	7.9%	-1.08 [-1.72, -0.44]
Abdikarim et al., 2015 (LP radical gastrectomy)	6.8	1.1	30	7.7	1.1	31	8.9%	-0.90 [-1.45, -0.35]
Hartsell et al., 1997 (Colorectal surgery)	7.2	3.3	29	8.1	2.3	29	2.9%	-0.90 [-2.36, 0.56]
Ren et al., 2012 (Colorectal radical res)	5.7	1.6	299	6.6	2.4	298	11.5%	-0.90 [-1.23, -0.57]
El Nakeeb et al., 2009 (Open colonic anast)	6.2	0.2	60	6.9	0.5	60	13.2%	-0.70 [-0.84, -0.56]
Reissman et al.,1995 (LPT Bowel resection)	6.2	0.2	80	6.8	0.2	81	13.6%	-0.60 [-0.66, -0.54]
**Total (95% CI)**			**1395**			**1424**	**100.0%**	**-1.31 [-1.59, -1.03]**

Heterogeneity: Tau² = 0.15; Chi² = 89.49, df = 15 (P < 0.00001); I² = 83%
Test for overall effect: Z = 9.24 (P < 0.00001)

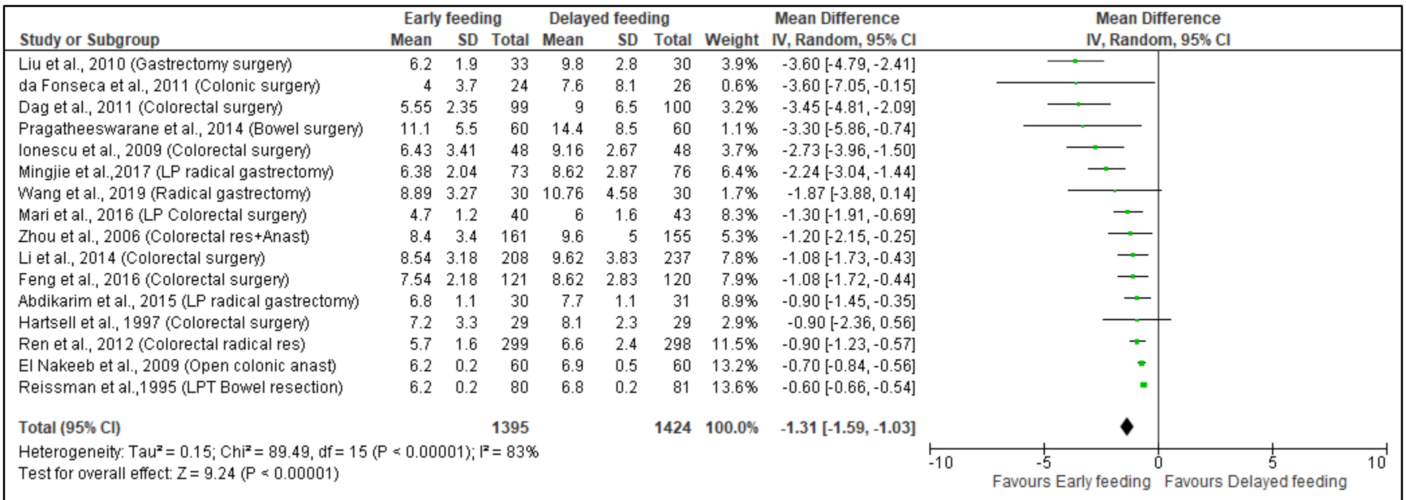

Note. LP; laparoscopic; LPT, Laparotomy; Res, Resection; Anast, Anastomosis

Supplementary File 9. Complications evaluated in the included articles

First author, year	Complication evaluated
Abdikarim 2015	Anastomotic leakage
El Nakeeb 2009	Anastomotic leakage
Hartsell 1997	Anastomotic leakage
Pragatheeswarane 2014	Anastomotic leakage
Reissman 1995	Anastomotic leakage
Zhou 2006	Anastomotic leakage
Liu 2010	Anastomotic leakage
Feng 2016	Anastomotic bleeding
Li 2014	Anastomotic bleeding
Cao 2020	Anastomotic bleeding ≥ C-D grade II
Cao 2020	Anastomotic bleeding ≥ C-D grade IIIa
Ortiz 1996	Anastomotic breakdown
Stewart 1998	Anastomotic dehiscence
Mari 2016	Anastomotic fistula
Wang 2019	Anastomotic fistula
Ionescu 2009	Anastomotic leakage
Ren 2012	Anastomotic leakage
Da Fonseca 2010	Anastomotic leakage
Dag 2011	Anastomotic leakage
Feng 2016	Anastomotic leakage
He 2015	anastomotic leakage
Li 2014	Anastomotic leakage
Shichinohe 2017	Anastomotic leakage
Wang 2011	Anastomotic leakage
Lee 2011	Anastomotic leakage
Lee 2013	Anastomotic leakage
Cao 2020	Anastomotic leakage ≥ C-D grade II
Cao 2020	Anastomotic leakage ≥ C-D grade IIIa
Li 2010	Anastomotic leakage
Consoli 2010	Anastomotic leakage
Liang 2017	Bile leakage
He 2015	Bile leakage
Cao 2020	Duodenal leakage ≥ C-D grade II
Cao 2020	Duodenal leakage ≥ C-D grade IIIa
Dag 2011	Evisceration
Shichinohe 2017	Fistula
Ortiz 1996	Ileostomy necrosis
Khoo 2007	Intestinal leaks
Mingjie 2017	Leakage
Cao 2020	Pancreatic fistula ≥ C-D grade II
Cao 2020	Pancreatic fistula ≥ C-D grade IIIa
Ren 2012	Pancreatic leakage
Ionescu 2009	Postoperative hernia
Hwang 2019	Postoperative pancreatic fistula ≥grade B
Lee 2013	Rectovaginal fistula
Mingjie 2017	Stenosis
Abdikarim 2015,	Stenosis
Wang 2019	Deep vein thrombosis
Liu 2010	Deep vein thrombosis
Da Fonseca 2010	Deep vein thrombosis
Shichinohe 2017	Thrombosis
Ortiz 1996	Venous thrombosis
Da Fonseca 2010	Angina pectoris
He 2015	Atrial fibrillation
Feo 2004	Cardiac arrhythmia
Khoo 2007	Cardiorespiratory compromise
Stewart 1998	Cardiovascular complication

Ren 2012	Cardiovascular and cerebrovascular complication
Hwang 2019	Cardiovascular complication
Cao 2020	Cardiovascular-associated complication
Cao 2020	Cardiovascular-associated complication
Dag 2011	Cerebral Infarct
Wang 2011	Intrapulmonary infection
Liang 2017	Liver failure
He 2015	Liver infection
He 2015	Lung infection
Liang 2017	Multiple organ failure
Shichinohe 2017	Neurogenic bladder
Hwang 2019	Neurologic complication
Hwang 2019	Organ failure Liver and Kidney
Da Fonseca 2010	Pancreatitis
Liang 2017	Pleural effusion
Li 2019	Pneumonia
Reissman 1995	Pneumonia
Shichinohe 2017	Pneumonia
Liang 2017	Pneumonia
Dag 2011	Pneumonia
Ortiz 1996	Pneumonia
Hwang 2019	Pulmonary complication
Liang 2017	Pulmonary embolism
Ionescu 2009	Pulmonary embolism
El Nakeeb 2009	Pulmonary infection
Feng 2016	Pulmonary infection
Li 2014	Pulmonary infection
Zhou 2006	Pulmonary infection
Wang 2019	Pulmonary infection
Feo 2004	pulmonary oedema
Cao 2020	Pulmonary-associated complication
Stewart 1998	Respiratory
Shichinohe 2017	Stroke
Dag 2011	Toxic Hepatitis
Hartsell 1997	Aspiration pneumonia
Da Fonseca 2010	Aspiration pneumonia
Da Fonseca 2010	Catheter sepsis
Ionescu 2009	Hematuria
Feo 2004	Anemia
Abdikarim 2015,	Bleeding
He 2015	Bleeding
Shichinohe 2017	Bleeding
Liang 2017	Hemorrhage and reoperation
Ortiz 1996	Hemorrhage
Cao 2020	Intra-abdominal bleeding
Hwang 2019	Post-pancreatectomy hemorrhage ≥grade B
Mingjie 2017	Postoperative bleeding
Feo 2004	Delirium
Cao 2020	Delirium
Wang 2011	Bowel obstruction
He 2015	Constipation
Abdikarim 2015,	Ileus
Feng 2016	Ileus
Cao 2020	Ileus
Mingjie 2017	Ileus
Shichinohe 2017	Ileus
Lee 2011	Ileus
Liu 2010	Ileus
Li 2019	Intestinal obstruction
Li 2014	Intestinal Obstruction

Ren 2012	Intestinal obstruction
Liang 2017	Intestinal obstruction
Wang 2019	Intestinal obstruction
Ortiz 1996	Intestinal obstruction
Reissman 1995	Intestinal obstruction
Kang 2018	one delayed return of bowel motility
Lee 2013	Postoperative ileus
Da Fonseca 2010	Prolonged ileus
Consoli 2010	Death
Liu 2010	Death
Stewart 1998	Deaths
Khoo 2007	Deaths
Pragatheeswarane 2014 et al,	Mortality
El Nakeeb 2009	Mortality
Consoli 2010	Diarrhea
Ren 2012	Diarrhea
Liu 2010	Diarrhea and vomiting
El Nakeeb 2009	Vomiting
Pragatheeswarane 2014 et al,	Vomiting
Feo 2004	Fever
Pragatheeswarane 2014 et al,	Fever
Zhou 2006	Fever
Li 2019	Incision infection
Wang 2011	Incision infection
Wang 2019	Incision infection
Hwang 2019	Infectious complication including surgical-site infection
Khoo 2007	Pressure sores
Liu 2010	Septic
Feo 2004	wound bleeding
El Nakeeb 2009	Wound complication
Zhou 2006	Wound complication
Kang 2018	wound complications and one patient had intraluminal bleeding that needed embolization
Feo 2004	wound dehiscence
Lee 2011	Wound discharge
Abdikarim 2015	Wound infection
Feng 2016	Wound infection
Lee 2013	Wound infection
Li 2014	Wound infection
Pragatheeswarane 2014 et al,	Wound infection
Reissman 1995	Wound infection
Ren 2012	Wound infection
Cao 2020	Wound infection
Liang 2017	Wound infection
Mingjie 2017	Wound infection
Shichinohe 2017	Wound infection
Dag 2011	Wound infection
Stewart 1998	Wound infection
Da Fonseca 2010	Wound infection
Ionescu 2009	Wound infection
Ortiz 1996	Wound infection
Liu 2010	Wound infection or breakdown
Shichinohe 2017	Meniere's disease
Feo 2004	allergic drug reaction
Hwang 2019	Reoperation
Hwang 2019	Readmission
Liu 2010	Readmission
Khoo 2007	Transient urinary retention
Ortiz 1996	Urinary infection
Feng 2016	Urinary retention

Lee 2011	Urinary retention
Li 2014	Urinary retention
Wang 2019	Urinary retention
He 2015	urinary tract infection
Reissman 1995	Urinary tract infection
Shichinohe 2017	Urinary tract infection
Cao 2020	Urinary tract infection
Wang 2019	Urinary tract infection
Khoo 2007	Urinary tract infection
Liu 2010	Urinary tract infection
Stewart 1998	Urinary tract infection
Ionescu 2009	Urinary tract infection
Wang 2019	Gastrointestinal stasis
Liang 2017	Abdominal abscess
Ortiz 1996	Abdominal abscess
Reissman 1995	Pelvic abscess
Pragatheeswarane et al, 2014	Abdominal distention
Kang 2018	Abdominal fluid collection that needed percutaneous drainage
Liu 2010	Abdominal infection
Zhou 2006	Acute dilation of stomach
Lee 2013	Acute voiding difficulty
Lee 2013	Chylous ascites
He 2015	Postoperative ascites
Hwang 2019	Delayed gastric emptying ≥grade B
Li 2019	Gastric retention
Ren 2012	Gastric retention
Ren 2012	Intestinal perforation
Shichinohe 2017	Intra-abdominal abscess
Cao 2020	Intra-abdominal infection
Cao 2020	Intra-abdominal infection
He 2015	Mild abdominal distension and nausea
Wang 2019	Nausea/Vomiting
Consoli 2010	Nausea/Vomiting
Wendler 2022	Nausea
Khoo 2007	NGT decompression
Liang 2017	Sepsis
Dag 2011	Sepsis
Hartsell 1997	Morbidity
Guebbels 2019	No specification in the results, except for narrative results: “The most frequent complications were staple line bleeding, leakage, pneumonia, dehydration and trocar site infection”
Kang 2018	Other complications were classified as Clavien-Dindo grade I
Liang 2017	Other minor
Pragatheeswarane 2014	Others
Feo 2004	Faintness of vagal origin
He 2015	hypokalemia
Mari 2016	laparotomic conversion due to peritoneal adhesions and bulky tumor
Zhou 2006	Pharyngolaryngitis

Supplementary File 10. Forest Plot for Complications

10.A Overall complications

Study or Subgroup	Early feeding Events	Early feeding Total	Routine care Events	Routine care Total	Weight	Risk Ratio M-H, Random, 95% CI
Wang et al., 2012	2	40	8	38	1.0%	0.24 [0.05, 1.05]
Zhou et al., 2006	21	161	66	155	5.7%	0.31 [0.20, 0.48]
Feng et al., 2016	7	121	17	120	2.5%	0.41 [0.18, 0.95]
Wendler et al., 2022	3	40	7	40	1.2%	0.43 [0.12, 1.54]
Liang et al., 2018	18	208	47	237	4.9%	0.44 [0.26, 0.73]
Li et al., 2019	12	100	25	100	3.8%	0.48 [0.26, 0.90]
da Fonseca et al., 2011	4	24	9	26	1.8%	0.48 [0.17, 1.36]
Lee et al., 2011	6	46	14	54	2.4%	0.50 [0.21, 1.20]
Abdikarim et al., 2015	1	30	2	31	0.4%	0.52 [0.05, 5.40]
Mari et al., 2016	1	40	2	43	0.4%	0.54 [0.05, 5.70]
Ionescu et al., 2009	6	48	11	48	2.2%	0.55 [0.22, 1.36]
Khoo et al., 2007	9	41	16	40	3.3%	0.55 [0.28, 1.09]
Cao et al., 2021	22	85	39	86	5.8%	0.57 [0.37, 0.88]
Liu et al., 2010	4	33	6	30	1.5%	0.61 [0.19, 1.94]
El Nakeeb et al., 2009	14	60	23	60	4.4%	0.61 [0.35, 1.07]
Li et al., 2014	21	58	34	61	6.1%	0.65 [0.43, 0.98]
Pragatheeswarane et al., 2014	24	60	33	60	6.4%	0.73 [0.49, 1.07]
Kang et al., 2018	6	46	9	51	2.1%	0.74 [0.28, 1.92]
Consoli et al., 2010	4	15	5	14	1.6%	0.75 [0.25, 2.23]
Wang et al., 2019	7	30	9	30	2.5%	0.78 [0.33, 1.82]
Stewart et al., 1998	10	40	12	40	3.2%	0.83 [0.41, 1.70]
Dag et al., 2011	12	99	14	100	3.2%	0.87 [0.42, 1.78]
Geubbels et al., 2019	7	110	8	110	2.0%	0.88 [0.33, 2.33]
Shichinohe et al., 2017	9	45	11	49	2.8%	0.89 [0.41, 1.95]
Feo et al., 2004	12	50	13	50	3.4%	0.92 [0.47, 1.82]
He et al., 2015	7	48	6	38	1.9%	0.92 [0.34, 2.52]
Hwang et al., 2019	64	123	68	124	8.6%	0.95 [0.75, 1.20]
Ren et al., 2012	28	299	29	298	5.0%	0.96 [0.59, 1.58]
Ortiz et al., 1996	17	93	18	95	4.0%	0.96 [0.53, 1.75]
Hartsell et al., 1997	1	29	1	29	0.3%	1.00 [0.07, 15.24]
Reissman et al.,1995	6	80	5	81	1.5%	1.22 [0.39, 3.82]
Lee et al., 2013	22	52	11	46	4.0%	1.77 [0.97, 3.24]
Mingjie et al.,2017	2	73	1	76	0.4%	2.08 [0.19, 22.47]
**Total (95% CI)**		**2427**		**2460**	**100.0%**	**0.69 [0.59, 0.80]**
Total events	389		579			

Heterogeneity: Tau² = 0.06; Chi² = 48.59, df = 32 (P = 0.03); I² = 34%
Test for overall effect: Z = 4.83 (P < 0.00001)

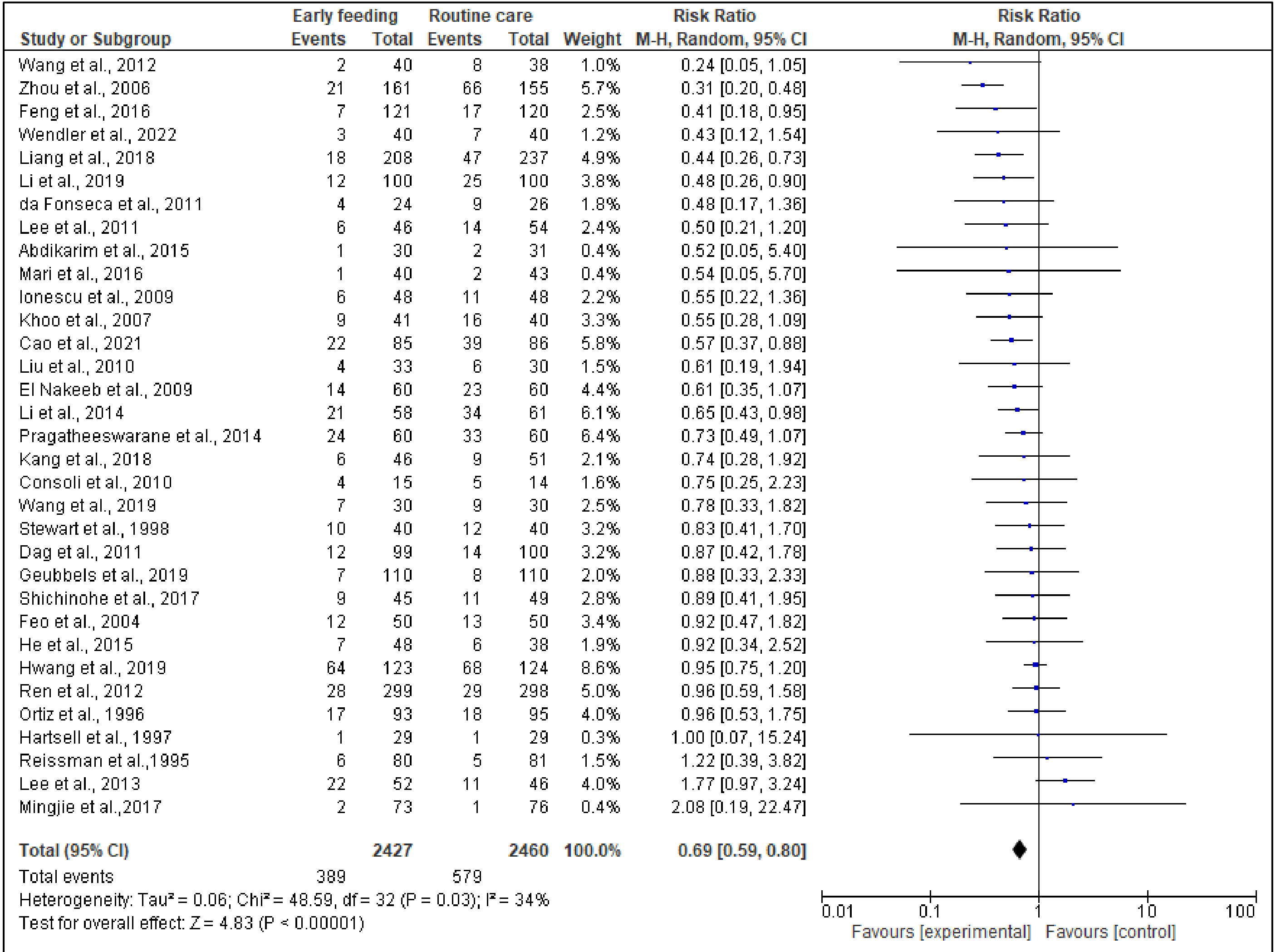

Note. AD, Adenocarcinoma; Ileosto, Ileostomy; LA, low anterior; LP; laparoscopic; LPT, Laparotomy; Res, Resection; Anast, Anastomosis

10.B. Vomiting *

Study or Subgroup	Early feeding Events	Early feeding Total	Routine care Events	Routine care Total	Weight	Risk Ratio M-H, Random, 95% CI
Liang et al., 2017	6	58	15	61	7.2%	0.42 [0.18, 1.01]
Wu et al., 2019	14	867	31	868	11.1%	0.45 [0.24, 0.84]
Feo et al., 2004	7	50	14	50	7.9%	0.50 [0.22, 1.13]
Pragatheeswarane et al., 2014	5	60	7	60	5.1%	0.71 [0.24, 2.13]
Ionescu et al., 2009	17	48	21	48	13.9%	0.81 [0.49, 1.33]
Da Fonseca et al., 2010	4	24	5	26	4.4%	0.87 [0.26, 2.85]
Liu et al., 2010	1	33	1	30	1.0%	0.91 [0.06, 13.90]
Stewart et al., 1998	14	40	14	40	11.6%	1.00 [0.55, 1.82]
Hartsell et al., 1997	14	29	10	29	11.0%	1.40 [0.75, 2.62]
El Nakeeb et al., 2009	15	60	10	60	9.4%	1.50 [0.73, 3.07]
Wang et al., 2019	3	30	2	30	2.3%	1.50 [0.27, 8.34]
Shichinohe et al., 2017	7	45	5	49	5.2%	1.52 [0.52, 4.46]
Reissman et al.,1995	17	80	11	81	9.8%	1.56 [0.78, 3.13]
**Total (95% CI)**		**1424**		**1432**	**100.0%**	**0.90 [0.68, 1.18]**
Total events	124		146			

Heterogeneity: Tau² = 0.08; Chi² = 17.75, df = 12 (P = 0.12); I² = 32%
Test for overall effect: Z = 0.76 (P = 0.44)

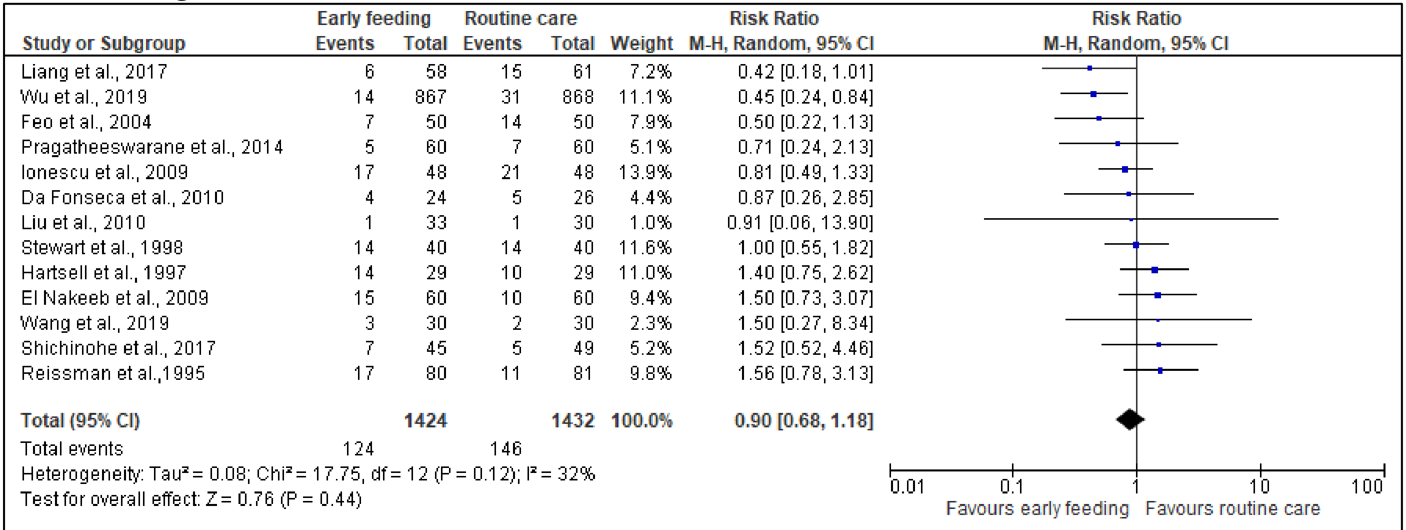

Note. LP; laparoscopic; LPT, Laparotomy; Res, Resection; Anast, Anastomosis

*Four studies evaluated PONV (da Fonseca et al., 2011; Ionescu et al., 2009; Liang et al., 2018; Wang et al., 2019), one diarrea and vomiting (Liu et al., 2010); eight vomiting (El Nakeeb et al., 2009; Feo et al., 2004; Hartsell et al., 1997; Pragatheeswarane et al., 2014; Reissman et al., 1995; Shichinohe et al., 2017; Stewart et al., 1998; Wu et al., 2019). Four studies evaluated both nausea and vomiting separately; we pooled in the metanalysis only data on vomiting for these studies (Feo et al., 2004; Hartsell et al., 1997; Shichinohe et al., 2017; Wu et al., 2019).

Supplementary File 11. Subgroup analysis for the outcome "First passage of the stool"

11.A Subgroup analysis according to the type of intervention for the outcome "First passage of the stool"

Study or Subgroup	Early feeding Mean	Early feeding SD	Early feeding Total	Delayed feeding Mean	Delayed feeding SD	Delayed feeding Total	Weight	Mean Difference IV, Random, 95% CI
**1.13.1 Early Feeding**								
Pragatheeswarane et al., 2014 (Bowel surgery)	3.8	1.3	60	6.1	2.1	60	6.8%	-2.30 [-2.92, -1.68]
Dag et al., 2011 (Colorectal surgery)	3.4	0.77	99	4.38	1.18	100	9.8%	-0.98 [-1.26, -0.70]
El Nakeeb et al., 2009 (Open colonic anast)	4.1	1.2	60	4.9	1.2	60	8.5%	-0.80 [-1.23, -0.37]
Zhou et al., 2006 (Colorectal res+Anast)	4.1	1.1	161	4.8	1.4	155	9.8%	-0.70 [-0.98, -0.42]
**Subtotal (95% CI)**			**380**			**375**	**34.9%**	**-1.13 [-1.62, -0.63]**
Heterogeneity: Tau² = 0.21; Chi² = 21.54, df = 3 (P < 0.0001); I² = 86%								
Test for overall effect: Z = 4.48 (P < 0.00001)								
**1.13.2 ERAS - Multicomponent intervention**								
Mingjie et al.,2017 (LP radical gastrectomy)	2.97	1.23	73	5.2	1.81	76	7.9%	-2.23 [-2.73, -1.73]
Wang et al., 2019 (Radical gastrectomy)	4.25	3.02	30	5.86	2.36	30	2.8%	-1.61 [-2.98, -0.24]
Feng et al., 2016 (Colorectal surgery)	4.84	1.59	121	5.83	2.05	120	8.2%	-0.99 [-1.45, -0.53]
Li et al., 2014 (Colorectal surgery)	4.84	1.59	208	5.83	2.05	237	9.3%	-0.99 [-1.33, -0.65]
Shichinohe et al., 2017 (LP Colorectal surgery)	2.2	1.6	45	3.1	1.6	49	6.6%	-0.90 [-1.55, -0.25]
Sun et al., 2017 (Major abdominal surgery)	2.04	0.29	53	2.58	0.21	54	10.9%	-0.54 [-0.64, -0.44]
Abdikarim et al., 2015 (LP radical gastrectomy)	3.1	0.7	30	3.6	0.8	31	9.0%	-0.50 [-0.88, -0.12]
Li et al., 2019 (LP Colorectal surgery)	2.51	0.72	100	2.89	0.65	100	10.4%	-0.38 [-0.57, -0.19]
**Subtotal (95% CI)**			**660**			**697**	**65.1%**	**-0.92 [-1.24, -0.59]**
Heterogeneity: Tau² = 0.16; Chi² = 59.25, df = 7 (P < 0.00001); I² = 88%								
Test for overall effect: Z = 5.58 (P < 0.00001)								
**Total (95% CI)**			**1040**			**1072**	**100.0%**	**-0.99 [-1.25, -0.72]**
Heterogeneity: Tau² = 0.16; Chi² = 93.68, df = 11 (P < 0.00001); I² = 88%								
Test for overall effect: Z = 7.34 (P < 0.00001)								
Test for subgroup differences: Chi² = 0.49, df = 1 (P = 0.48), I² = 0%								

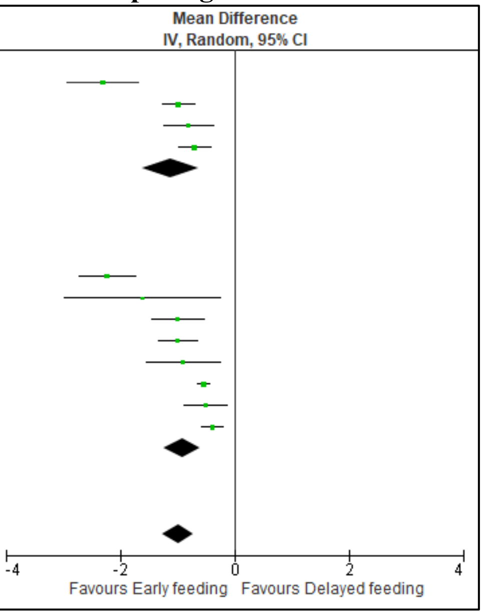

Note. LP; laparoscopic; Res, Resection; Anast, Anastomosis

11.B Subgroup analysis according to the site of intervention for the outcome "First passage of the stool"

Study or Subgroup	Early feeding Mean	Early feeding SD	Early feeding Total	Delayed feeding Mean	Delayed feeding SD	Delayed feeding Total	Weight	Mean Difference IV, Random, 95% CI
**1.14.1 Colon - Colonrectal**								
Dag et al., 2011 (Colorectal surgery)	3.4	0.77	99	4.38	1.18	100	9.8%	-0.98 [-1.26, -0.70]
El Nakeeb et al., 2009 (Open colonic anast)	4.1	1.2	60	4.9	1.2	60	8.5%	-0.80 [-1.23, -0.37]
Feng et al., 2016 (Colorectal surgery)	4.84	1.59	121	5.83	2.05	120	8.2%	-0.99 [-1.45, -0.53]
Li et al., 2014 (Colorectal surgery)	4.84	1.59	208	5.83	2.05	237	9.3%	-0.99 [-1.33, -0.65]
Li et al., 2019 (LP Colorectal surgery)	2.51	0.72	100	2.89	0.65	100	10.4%	-0.38 [-0.57, -0.19]
Shichinohe et al., 2017 (LP Colorectal surgery)	2.2	1.6	45	3.1	1.6	49	6.6%	-0.90 [-1.55, -0.25]
Zhou et al., 2006 (Colorectal res+Anast)	4.1	1.1	161	4.8	1.4	155	9.8%	-0.70 [-0.98, -0.42]
**Subtotal (95% CI)**			**794**			**821**	**62.7%**	**-0.79 [-1.02, -0.56]**
Heterogeneity: Tau² = 0.06; Chi² = 19.71, df = 6 (P = 0.003); I² = 70%								
Test for overall effect: Z = 6.77 (P < 0.00001)								
**1.14.2 Bowel - Abdominal - Gastrectomy**								
Abdikarim et al., 2015 (LP radical gastrectomy)	3.1	0.7	30	3.6	0.8	31	9.0%	-0.50 [-0.88, -0.12]
Mingjie et al.,2017 (LP radical gastrectomy)	2.97	1.23	73	5.2	1.81	76	7.9%	-2.23 [-2.73, -1.73]
Reissman et al.,1995 (LPT Bowel resection)	3.8	1.3	60	6.1	2.1	60	6.8%	-2.30 [-2.92, -1.68]
Sun et al., 2017 (Major abdominal surgery)	2.04	0.29	53	2.58	0.21	54	10.9%	-0.54 [-0.64, -0.44]
Wang et al., 2019 (Radical gastrectomy)	4.25	3.02	30	5.86	2.36	30	2.8%	-1.61 [-2.98, -0.24]
**Subtotal (95% CI)**			**246**			**251**	**37.3%**	**-1.39 [-2.17, -0.61]**
Heterogeneity: Tau² = 0.69; Chi² = 73.31, df = 4 (P < 0.00001); I² = 95%								
Test for overall effect: Z = 3.48 (P = 0.0005)								
**Total (95% CI)**			**1040**			**1072**	**100.0%**	**-0.99 [-1.25, -0.72]**
Heterogeneity: Tau² = 0.16; Chi² = 93.68, df = 11 (P < 0.00001); I² = 88%								
Test for overall effect: Z = 7.34 (P < 0.00001)								
Test for subgroup differences: Chi² = 2.07, df = 1 (P = 0.15), I² = 51.7%								

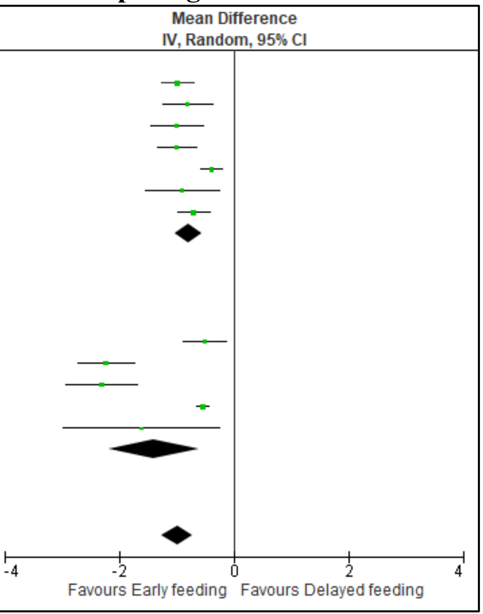

Note. LP; laparoscopic; Res, Resection; Anast, Anastomosis

Supplementary File 12. Subgroup analysis for the outcome “First flatus”

12.A Subgroup analysis according to the type of intervention for the outcome “First flatus”

Study or Subgroup	Early feeding Mean	Early feeding SD	Early feeding Total	Delayed feeding Mean	Delayed feeding SD	Delayed feeding Total	Weight	Mean Difference IV, Random, 95% CI
**1.3.1 Early feeding**								
Pragatheeswarane et al., 2014 (Bowel surgery)	2.6	0.9	60	4.5	1.5	60	6.1%	-1.90 [-2.34, -1.46]
El Nakeeb et al., 2009 (Open colonic anast)	3.3	0.9	60	4.2	1.2	60	6.9%	-0.90 [-1.28, -0.52]
Zhou et al., 2006 (Colorectal res+Anast)	4.1	1.1	161	4.8	1.4	155	8.2%	-0.70 [-0.98, -0.42]
da Fonseca et al., 2011 (Colonic surgery)	1.5	0.5	24	2	0.7	26	7.4%	-0.50 [-0.84, -0.16]
**Subtotal (95% CI)**			**305**			**301**	**28.6%**	**-0.98 [-1.51, -0.45]**
Heterogeneity: Tau² = 0.26; Chi² = 26.94, df = 3 (P < 0.00001); I² = 89%								
Test for overall effect: Z = 3.65 (P = 0.0003)								
**1.3.2 ERAS - Multicomponent**								
Liu et al., 2010 (Gastrectomy surgery)	3.2	0.8	33	4.6	0.8	30	6.7%	-1.40 [-1.80, -1.00]
Mari et al., 2016 (LP Colorectal surgery)	1.5	0.6	40	2.4	0.9	43	7.6%	-0.90 [-1.23, -0.57]
Wang et al., 2019 (Radical gastrectomy)	2.63	1.07	30	3.35	1.32	30	4.5%	-0.72 [-1.33, -0.11]
Feng et al., 2016 (Colorectal surgery)	3.71	1.14	121	4.26	1.52	120	7.4%	-0.55 [-0.89, -0.21]
Li et al., 2014 (Colorectal surgery)	3.71	1.14	208	4.26	1.52	237	8.6%	-0.55 [-0.80, -0.30]
Li et al., 2019 (LP Colorectal surgery)	2.35	0.52	100	2.8	0.77	100	9.4%	-0.45 [-0.63, -0.27]
Sun et al., 2017 (Major abdominal surgery)	1.37	0.22	53	1.79	0.2	54	10.3%	-0.42 [-0.50, -0.34]
Ren et al., 2012 (Colorectal radical res)	2.24	0.71	299	2.63	0.83	298	9.9%	-0.39 [-0.51, -0.27]
Shichinohe et al., 2017 (LP Colorectal surgery)	1.2	0.9	45	1.5	0.9	49	7.1%	-0.30 [-0.66, 0.06]
**Subtotal (95% CI)**			**929**			**961**	**71.4%**	**-0.58 [-0.72, -0.43]**
Heterogeneity: Tau² = 0.03; Chi² = 33.07, df = 8 (P < 0.0001); I² = 76%								
Test for overall effect: Z = 7.54 (P < 0.00001)								
**Total (95% CI)**			**1234**			**1262**	**100.0%**	**-0.70 [-0.87, -0.53]**
Heterogeneity: Tau² = 0.07; Chi² = 79.39, df = 12 (P < 0.00001); I² = 85%								
Test for overall effect: Z = 8.09 (P < 0.00001)								
Test for subgroup differences: Chi² = 2.12, df = 1 (P = 0.15), I² = 52.7%								

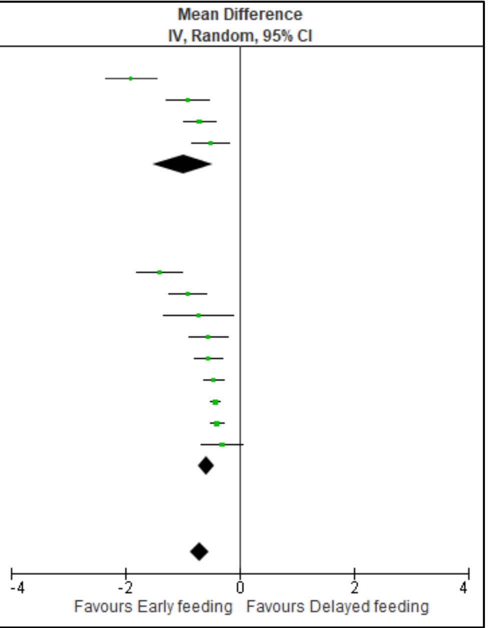

Note. LP; laparoscopic; Res, Resection; Anast, Anastomosis

12.B Subgroup analysis according to the site of intervention for the outcome “First flatus”

Study or Subgroup	Early feeding Mean	Early feeding SD	Early feeding Total	Delayed feeding Mean	Delayed feeding SD	Delayed feeding Total	Weight	Mean Difference IV, Random, 95% CI
**1.4.1 Colon and rectal**								
El Nakeeb et al., 2009 (Open colonic anast)	3.3	0.9	60	4.2	1.2	60	6.9%	-0.90 [-1.28, -0.52]
Mari et al., 2016 (LP Colorectal surgery)	1.5	0.6	40	2.4	0.9	43	7.6%	-0.90 [-1.23, -0.57]
Zhou et al., 2006 (Colorectal res+Anast)	4.1	1.1	161	4.8	1.4	155	8.2%	-0.70 [-0.98, -0.42]
Feng et al., 2016 (Colorectal surgery)	3.71	1.14	121	4.26	1.52	120	7.4%	-0.55 [-0.89, -0.21]
Li et al., 2014 (Colorectal surgery)	3.71	1.14	208	4.26	1.52	237	8.6%	-0.55 [-0.80, -0.30]
da Fonseca et al., 2011 (Colonic surgery)	1.5	0.5	24	2	0.7	26	7.4%	-0.50 [-0.84, -0.16]
Li et al., 2019 (LP Colorectal surgery)	2.35	0.52	100	2.8	0.77	100	9.4%	-0.45 [-0.63, -0.27]
Ren et al., 2012 (Colorectal radical res)	2.24	0.71	299	2.63	0.83	298	9.9%	-0.39 [-0.51, -0.27]
Shichinohe et al., 2017 (LP Colorectal surgery)	1.2	0.9	45	1.5	0.9	49	7.1%	-0.30 [-0.66, 0.06]
**Subtotal (95% CI)**			**1058**			**1088**	**72.5%**	**-0.55 [-0.68, -0.43]**
Heterogeneity: Tau² = 0.02; Chi² = 16.71, df = 8 (P = 0.03); I² = 52%								
Test for overall effect: Z = 8.64 (P < 0.00001)								
**1.4.2 Bowel - Abdominal - Gastric surgery**								
Pragatheeswarane et al., 2014 (Bowel surgery)	2.6	0.9	60	4.5	1.5	60	6.1%	-1.90 [-2.34, -1.46]
Liu et al., 2010 (Gastrectomy surgery)	3.2	0.8	33	4.6	0.8	30	6.7%	-1.40 [-1.80, -1.00]
Wang et al., 2019 (Radical gastrectomy)	2.63	1.07	30	3.35	1.32	30	4.5%	-0.72 [-1.33, -0.11]
Sun et al., 2017 (Major abdominal surgery)	1.37	0.22	53	1.79	0.2	54	10.3%	-0.42 [-0.50, -0.34]
**Subtotal (95% CI)**			**176**			**174**	**27.5%**	**-1.10 [-1.88, -0.33]**
Heterogeneity: Tau² = 0.58; Chi² = 62.67, df = 3 (P < 0.00001); I² = 95%								
Test for overall effect: Z = 2.79 (P = 0.005)								
**Total (95% CI)**			**1234**			**1262**	**100.0%**	**-0.70 [-0.87, -0.53]**
Heterogeneity: Tau² = 0.07; Chi² = 79.39, df = 12 (P < 0.00001); I² = 85%								
Test for overall effect: Z = 8.09 (P < 0.00001)								
Test for subgroup differences: Chi² = 1.88, df = 1 (P = 0.17), I² = 46.8%								

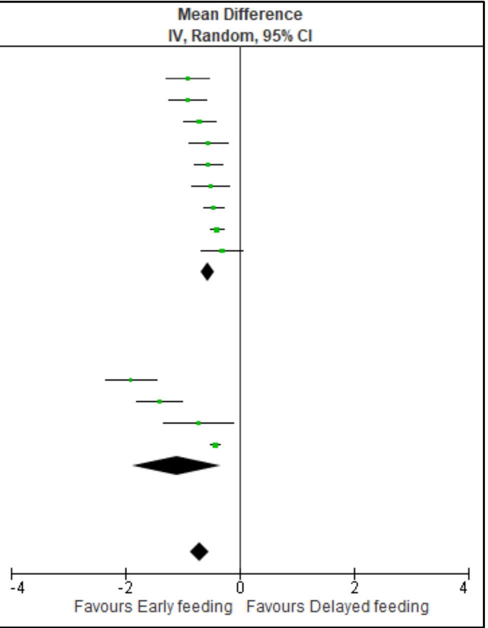

Note. LP; laparoscopic; Res, Resection; Anast, Anastomosis

Supplementary File 13. Subgroup analysis for the outcome “Length of hospitalization”

13.A Subgroup analysis according to the type of intervention for the outcome “Length of hospitalization”

	Early feeding			Delayed feeding				Mean Difference
Study or Subgroup	Mean	SD	Total	Mean	SD	Total	Weight	IV, Random, 95% CI
**1.9.1 Early feeding**								
da Fonseca et al., 2011 (Colonic surgery)	4	3.7	24	7.6	8.1	26	0.6%	-3.60 [-7.05, -0.15]
Dag et al., 2011 (Colorectal surgery)	5.55	2.35	99	9	6.5	100	3.2%	-3.45 [-4.81, -2.09]
Pragatheeswarane et al., 2014 (Bowel surgery)	11.1	5.5	60	14.4	8.5	60	1.1%	-3.30 [-5.86, -0.74]
Zhou et al., 2006 (Colorectal res+Anast)	8.4	3.4	161	9.6	5	155	5.3%	-1.20 [-2.15, -0.25]
Hartsell et al., 1997 (Colorectal surgery)	7.2	3.3	29	8.1	2.3	29	2.9%	-0.90 [-2.36, 0.56]
El Nakeeb et al., 2009 (Open colonic anast)	6.2	0.2	60	6.9	0.5	60	13.2%	-0.70 [-0.84, -0.56]
Reissman et al.,1995 (LPT Bowel resection)	6.2	0.2	80	6.8	0.2	81	13.6%	-0.60 [-0.66, -0.54]
**Subtotal (95% CI)**			**513**			**511**	**39.9%**	**-0.90 [-1.20, -0.59]**
Heterogeneity: Tau² = 0.06; Chi² = 27.08, df = 6 (P = 0.0001); I² = 78%								
Test for overall effect: Z = 5.68 (P < 0.00001)								
**1.9.2 ERAS - Multicomponent**								
Wang et al., 2019 (Radical gastrectomy)	6.2	1.9	33	9.8	2.8	30	3.9%	-3.60 [-4.79, -2.41]
Ionescu et al., 2009 (Colorectal surgery)	6.43	3.41	48	9.16	2.67	48	3.7%	-2.73 [-3.96, -1.50]
Feng et al., 2016 (Colorectal surgery)	6.38	2.04	73	8.62	2.87	76	6.4%	-2.24 [-3.04, -1.44]
Liu et al., 2010 (Gastrectomy surgery)	8.89	3.27	30	10.76	4.58	30	1.7%	-1.87 [-3.88, 0.14]
Mari et al., 2016 (LP Colorectal surgery)	4.7	1.2	40	6	1.6	43	8.3%	-1.30 [-1.91, -0.69]
Ren et al., 2012 (Colorectal radical res)	8.54	3.18	208	9.62	3.83	237	7.8%	-1.08 [-1.73, -0.43]
Mingjie et al.,2017 (LP radical gastrectomy)	7.54	2.18	121	8.62	2.83	120	7.9%	-1.08 [-1.72, -0.44]
Abdikarim et al., 2015 (LP radical gastrectomy)	6.8	1.1	30	7.7	1.1	31	8.9%	-0.90 [-1.45, -0.35]
Li et al., 2014 (Colorectal surgery)	5.7	1.6	299	6.6	2.4	298	11.5%	-0.90 [-1.23, -0.57]
**Subtotal (95% CI)**			**882**			**913**	**60.1%**	**-1.56 [-2.04, -1.08]**
Heterogeneity: Tau² = 0.36; Chi² = 33.20, df = 8 (P < 0.0001); I² = 76%								
Test for overall effect: Z = 6.37 (P < 0.00001)								
**Total (95% CI)**			**1395**			**1424**	**100.0%**	**-1.31 [-1.59, -1.03]**
Heterogeneity: Tau² = 0.15; Chi² = 89.49, df = 15 (P < 0.00001); I² = 83%								
Test for overall effect: Z = 9.24 (P < 0.00001)								
Test for subgroup differences: Chi² = 5.23, df = 1 (P = 0.02), I² = 80.9%								

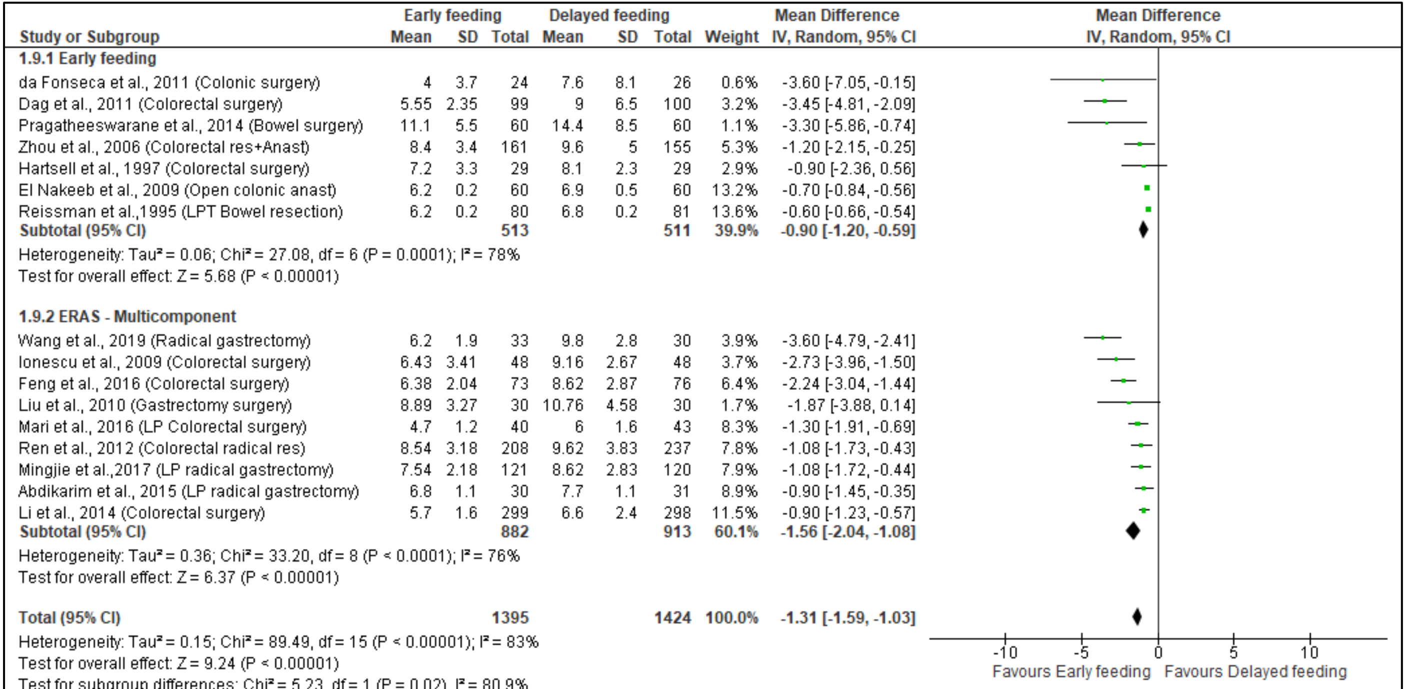

Note. LP; laparoscopic; LPT, Laparotomy; Res, Resection; Anast, Anastomosis

13.B Subgroup analysis according to the site of intervention for the outcome “Length of hospitalization”

	Early feeding			Delayed feeding				Mean Difference
Study or Subgroup	Mean	SD	Total	Mean	SD	Total	Weight	IV, Random, 95% CI
**1.11.1 Colon and rectal surgery**								
da Fonseca et al., 2011 (Colonic surgery)	4	3.7	24	7.6	8.1	26	0.7%	-3.60 [-7.05, -0.15]
Dag et al., 2011 (Colorectal surgery)	5.55	2.35	99	9	6.5	100	3.5%	-3.45 [-4.81, -2.09]
Ionescu et al., 2009 (Colorectal surgery)	6.43	3.41	48	9.16	2.67	48	4.1%	-2.73 [-3.96, -1.50]
Mari et al., 2016 (LP Colorectal surgery)	4.7	1.2	40	6	1.6	43	9.0%	-1.30 [-1.91, -0.69]
Zhou et al., 2006 (Colorectal res+Anast)	8.4	3.4	161	9.6	5	155	5.8%	-1.20 [-2.15, -0.25]
Feng et al., 2016 (Colorectal surgery)	7.54	2.18	121	8.62	2.83	120	8.6%	-1.08 [-1.72, -0.44]
Ren et al., 2012 (Colorectal radical res)	5.7	1.6	299	6.6	2.4	298	12.4%	-0.90 [-1.23, -0.57]
Hartsell et al., 1997 (Colorectal surgery)	7.2	3.3	29	8.1	2.3	29	3.1%	-0.90 [-2.36, 0.56]
El Nakeeb et al., 2009 (Open colonic anast)	6.2	0.2	60	6.9	0.5	60	14.3%	-0.70 [-0.84, -0.56]
**Subtotal (95% CI)**			**881**			**879**	**61.5%**	**-1.32 [-1.75, -0.90]**
Heterogeneity: Tau² = 0.23; Chi² = 33.18, df = 8 (P < 0.0001); I² = 76%								
Test for overall effect: Z = 6.10 (P < 0.00001)								
**1.11.2 Bowel - Abdominal - Gastric surgery**								
Liu et al., 2010 (Gastrectomy surgery)	6.2	1.9	33	9.8	2.8	30	4.2%	-3.60 [-4.79, -2.41]
Pragatheeswarane et al., 2014 (Bowel surgery)	11.1	5.5	60	14.4	8.5	60	1.2%	-3.30 [-5.86, -0.74]
Mingjie et al.,2017 (LP radical gastrectomy)	6.38	2.04	73	8.62	2.87	76	7.0%	-2.24 [-3.04, -1.44]
Wang et al., 2019 (Radical gastrectomy)	8.89	3.27	30	10.76	4.58	30	1.8%	-1.87 [-3.88, 0.14]
Abdikarim et al., 2015 (LP radical gastrectomy)	6.8	1.1	30	7.7	1.1	31	9.6%	-0.90 [-1.45, -0.35]
Reissman et al.,1995 (LPT Bowel resection)	6.2	0.2	80	6.8	0.2	81	14.6%	-0.60 [-0.66, -0.54]
**Subtotal (95% CI)**			**306**			**308**	**38.5%**	**-1.85 [-2.76, -0.93]**
Heterogeneity: Tau² = 0.93; Chi² = 46.88, df = 5 (P < 0.00001); I² = 89%								
Test for overall effect: Z = 3.97 (P < 0.0001)								
**Total (95% CI)**			**1187**			**1187**	**100.0%**	**-1.33 [-1.63, -1.04]**
Heterogeneity: Tau² = 0.15; Chi² = 87.94, df = 14 (P < 0.00001); I² = 84%								
Test for overall effect: Z = 8.97 (P < 0.00001)								
Test for subgroup differences: Chi² = 1.04, df = 1 (P = 0.31), I² = 3.5%								

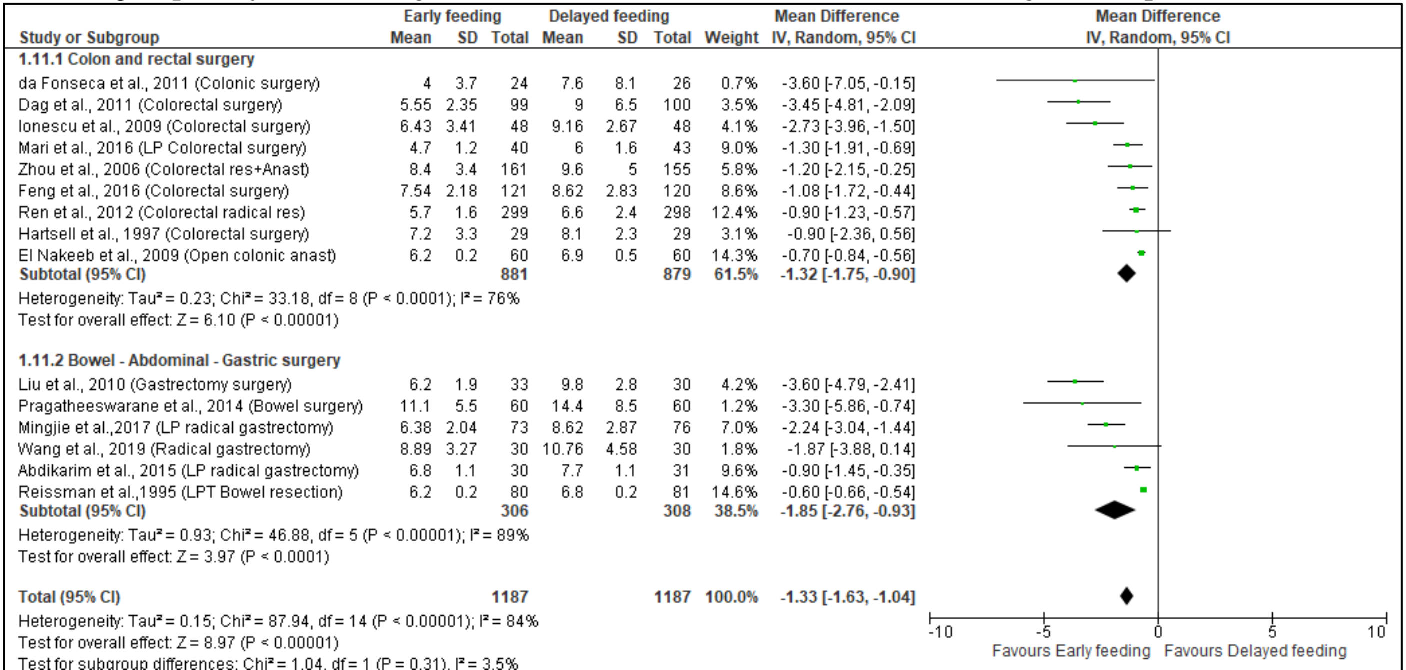

Note. LP; laparoscopic; LPT, Laparotomy; Res, Resection; Anast, Anastomosis

Supplementary File 14. Sensitivity analysis for the outcome “First passage of the stool”

14.A Only moderate or low risk of bias studies

Study or Subgroup	Early feeding Mean	Early feeding SD	Early feeding Total	Delayed feeding Mean	Delayed feeding SD	Delayed feeding Total	Weight	Mean Difference IV, Random, 95% CI
Pragatheeswarane et al., 2014	3.8	1.3	60	6.1	2.1	60	7.5%	-2.30 [-2.92, -1.68]
Mingjie et al.,2017	2.97	1.23	73	5.2	1.81	76	8.7%	-2.23 [-2.73, -1.73]
Wang et al., 2019	4.25	3.02	30	5.86	2.36	30	3.1%	-1.61 [-2.98, -0.24]
Feng et al., 2016	4.84	1.59	121	5.83	2.05	120	9.0%	-0.99 [-1.45, -0.53]
Li et al., 2014	4.84	1.59	208	5.83	2.05	237	10.2%	-0.99 [-1.33, -0.65]
Dag et al., 2011	3.4	0.77	99	4.38	1.18	100	10.7%	-0.98 [-1.26, -0.70]
Shichinohe et al., 2017	2.2	1.6	45	3.1	1.6	49	7.3%	-0.90 [-1.55, -0.25]
El Nakeeb et al., 2009	4.1	1.2	60	4.9	1.2	60	0.0%	-0.80 [-1.23, -0.37]
Zhou et al., 2006	4.1	1.1	161	4.8	1.4	155	10.7%	-0.70 [-0.98, -0.42]
Sun et al., 2017	2.04	0.29	53	2.58	0.21	54	11.7%	-0.54 [-0.64, -0.44]
Abdikarim et al., 2015	3.1	0.7	30	3.6	0.8	31	9.8%	-0.50 [-0.88, -0.12]
Li et al., 2019	2.51	0.72	100	2.89	0.65	100	11.3%	-0.38 [-0.57, -0.19]
**Total (95% CI)**			**980**			**1012**	**100.0%**	**-1.01 [-1.29, -0.73]**

Heterogeneity: $Tau^2 = 0.17$; $Chi^2 = 93.25$, df = 10 (P < 0.00001); $I^2 = 89\%$
Test for overall effect: Z = 6.99 (P < 0.00001)

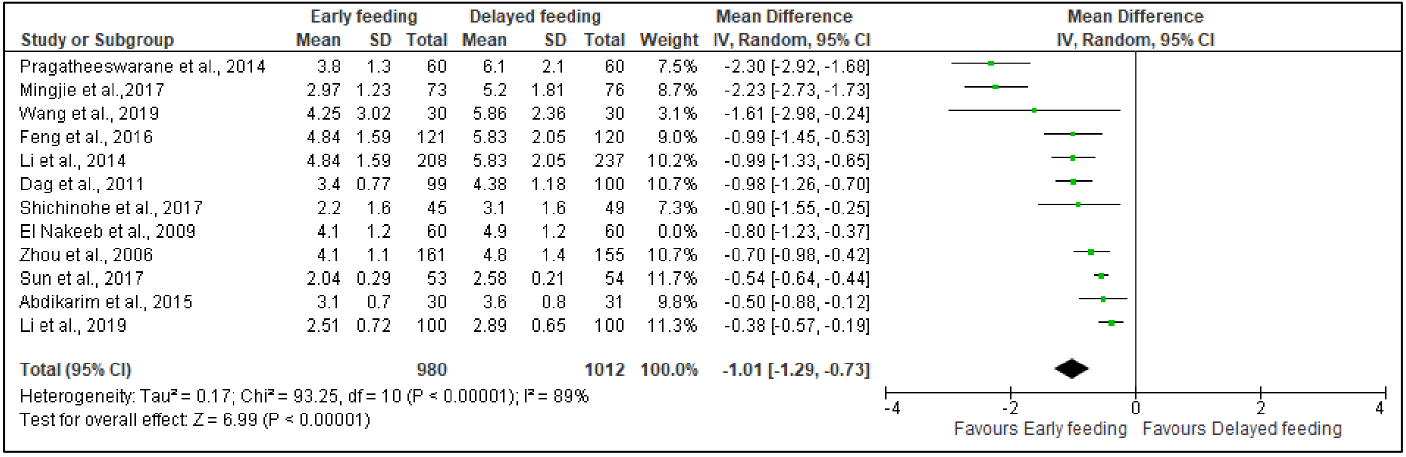

14.B Only studies with more than 100 participants

Study or Subgroup	Early feeding Mean	Early feeding SD	Early feeding Total	Delayed feeding Mean	Delayed feeding SD	Delayed feeding Total	Weight	Mean Difference IV, Random, 95% CI
Pragatheeswarane et al., 2014	3.8	1.3	60	6.1	2.1	60	8.5%	-2.30 [-2.92, -1.68]
Mingjie et al.,2017	2.97	1.23	73	5.2	1.81	76	9.8%	-2.23 [-2.73, -1.73]
Wang et al., 2019	4.25	3.02	30	5.86	2.36	30	0.0%	-1.61 [-2.98, -0.24]
Feng et al., 2016	4.84	1.59	121	5.83	2.05	120	10.2%	-0.99 [-1.45, -0.53]
Li et al., 2014	4.84	1.59	208	5.83	2.05	237	11.4%	-0.99 [-1.33, -0.65]
Dag et al., 2011	3.4	0.77	99	4.38	1.18	100	12.0%	-0.98 [-1.26, -0.70]
Shichinohe et al., 2017	2.2	1.6	45	3.1	1.6	49	0.0%	-0.90 [-1.55, -0.25]
El Nakeeb et al., 2009	4.1	1.2	60	4.9	1.2	60	10.5%	-0.80 [-1.23, -0.37]
Zhou et al., 2006	4.1	1.1	161	4.8	1.4	155	11.9%	-0.70 [-0.98, -0.42]
Sun et al., 2017	2.04	0.29	53	2.58	0.21	54	13.1%	-0.54 [-0.64, -0.44]
Abdikarim et al., 2015	3.1	0.7	30	3.6	0.8	31	0.0%	-0.50 [-0.88, -0.12]
Li et al., 2019	2.51	0.72	100	2.89	0.65	100	12.6%	-0.38 [-0.57, -0.19]
**Total (95% CI)**			**935**			**962**	**100.0%**	**-1.03 [-1.34, -0.73]**

Heterogeneity: $Tau^2 = 0.18$; $Chi^2 = 90.61$, df = 8 (P < 0.00001); $I^2 = 91\%$
Test for overall effect: Z = 6.64 (P < 0.00001)

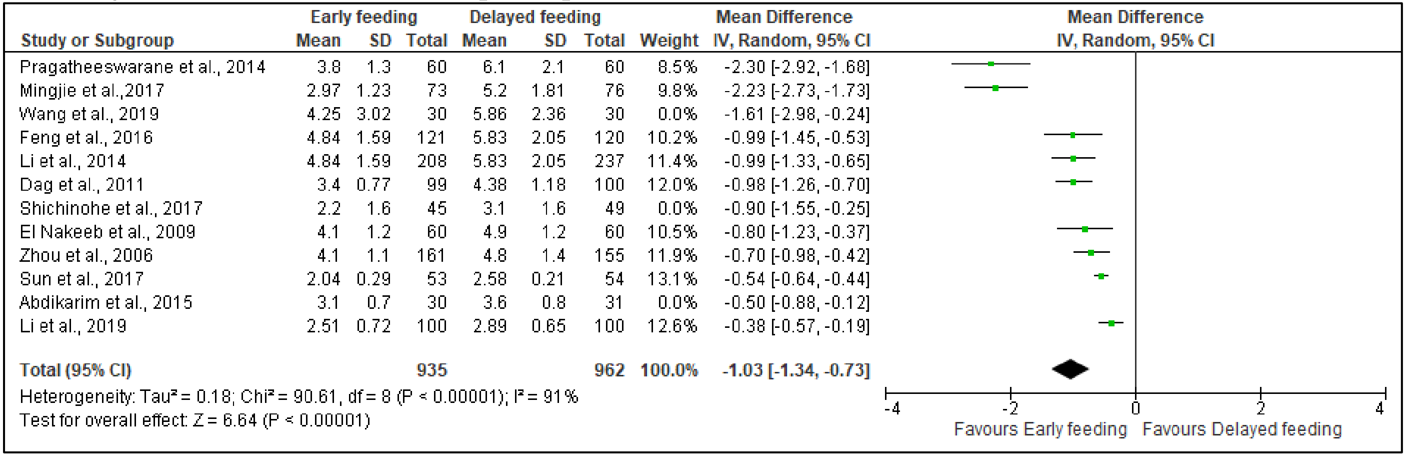

Supplementary File 15. Sensitivity analysis for the outcome "First passage of the flatus"

15.A Only moderate or low risk of bias studies

Study or Subgroup	Early feeding Mean	Early feeding SD	Early feeding Total	Delayed feeding Mean	Delayed feeding SD	Delayed feeding Total	Weight	Mean Difference IV, Random, 95% CI
Pragatheeswarane et al., 2014	2.6	0.9	60	4.5	1.5	60	7.1%	-1.90 [-2.34, -1.46]
Liu et al., 2010	3.2	0.8	33	4.6	0.8	30	7.8%	-1.40 [-1.80, -1.00]
El Nakeeb et al., 2009	3.3	0.9	60	4.2	1.2	60	0.0%	-0.90 [-1.28, -0.52]
Mari et al., 2016	1.5	0.6	40	2.4	0.9	43	0.0%	-0.90 [-1.23, -0.57]
Wang et al., 2019	2.63	1.07	30	3.35	1.32	30	5.1%	-0.72 [-1.33, -0.11]
Zhou et al., 2006	4.1	1.1	161	4.8	1.4	155	9.6%	-0.70 [-0.98, -0.42]
Feng et al., 2016	3.71	1.14	121	4.26	1.52	120	8.6%	-0.55 [-0.89, -0.21]
Li et al., 2014	3.71	1.14	208	4.26	1.52	237	10.0%	-0.55 [-0.80, -0.30]
da Fonseca et al., 2011	1.5	0.5	24	2	0.7	26	8.7%	-0.50 [-0.84, -0.16]
Li et al., 2019	2.35	0.52	100	2.8	0.77	100	11.0%	-0.45 [-0.63, -0.27]
Sun et al., 2017	1.37	0.22	53	1.79	0.2	54	12.1%	-0.42 [-0.50, -0.34]
Ren et al., 2012	2.24	0.71	299	2.63	0.83	298	11.7%	-0.39 [-0.51, -0.27]
Shichinohe et al., 2017	1.2	0.9	45	1.5	0.9	49	8.2%	-0.30 [-0.66, 0.06]
**Total (95% CI)**			**1134**			**1159**	**100.0%**	**-0.66 [-0.84, -0.48]**

Heterogeneity: Tau² = 0.07; Chi² = 69.04, df = 10 (P < 0.00001); I² = 86%
Test for overall effect: Z = 7.23 (P < 0.00001)

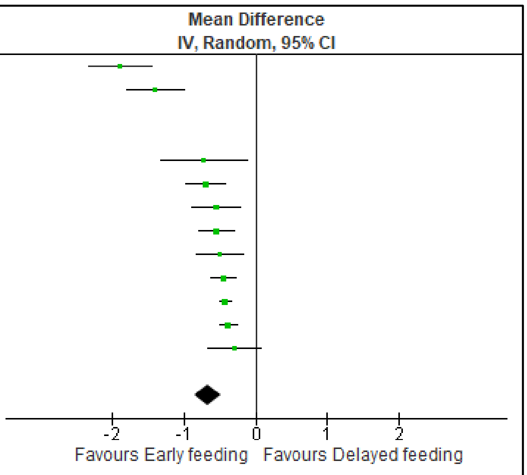

15.B Only studies with more than 100 participants

Study or Subgroup	Early feeding Mean	Early feeding SD	Early feeding Total	Delayed feeding Mean	Delayed feeding SD	Delayed feeding Total	Weight	Mean Difference IV, Random, 95% CI
Pragatheeswarane et al., 2014	2.6	0.9	60	4.5	1.5	60	8.7%	-1.90 [-2.34, -1.46]
Liu et al., 2010	3.2	0.8	33	4.6	0.8	30	0.0%	-1.40 [-1.80, -1.00]
El Nakeeb et al., 2009	3.3	0.9	60	4.2	1.2	60	9.9%	-0.90 [-1.28, -0.52]
Mari et al., 2016	1.5	0.6	40	2.4	0.9	43	0.0%	-0.90 [-1.23, -0.57]
Wang et al., 2019	2.63	1.07	30	3.35	1.32	30	0.0%	-0.72 [-1.33, -0.11]
Zhou et al., 2006	4.1	1.1	161	4.8	1.4	155	12.2%	-0.70 [-0.98, -0.42]
Feng et al., 2016	3.71	1.14	121	4.26	1.52	120	10.8%	-0.55 [-0.89, -0.21]
Li et al., 2014	3.71	1.14	208	4.26	1.52	237	12.8%	-0.55 [-0.80, -0.30]
da Fonseca et al., 2011	1.5	0.5	24	2	0.7	26	0.0%	-0.50 [-0.84, -0.16]
Li et al., 2019	2.35	0.52	100	2.8	0.77	100	14.3%	-0.45 [-0.63, -0.27]
Sun et al., 2017	1.37	0.22	53	1.79	0.2	54	16.0%	-0.42 [-0.50, -0.34]
Ren et al., 2012	2.24	0.71	299	2.63	0.83	298	15.4%	-0.39 [-0.51, -0.27]
Shichinohe et al., 2017	1.2	0.9	45	1.5	0.9	49	0.0%	-0.30 [-0.66, 0.06]
**Total (95% CI)**			**1062**			**1084**	**100.0%**	**-0.66 [-0.85, -0.47]**

Heterogeneity: Tau² = 0.06; Chi² = 51.39, df = 7 (P < 0.00001); I² = 86%
Test for overall effect: Z = 6.81 (P < 0.00001)

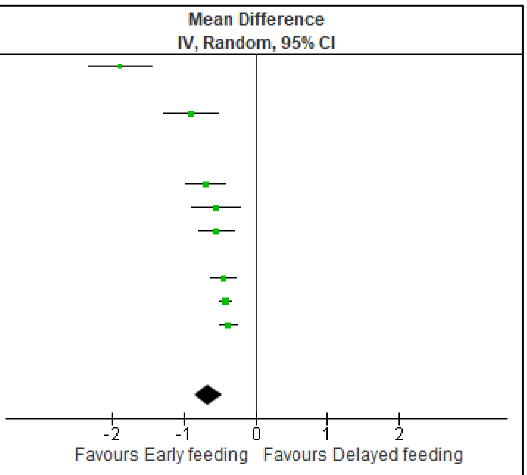

Supplementary File 16. Sensitivity analysis for the outcome "Length of Hospitalization"

16.A Only moderate or low risk of bias studies

Study or Subgroup	Early feeding Mean	Early feeding SD	Early feeding Total	Delayed feeding Mean	Delayed feeding SD	Delayed feeding Total	Weight	Mean Difference IV, Random, 95% CI
Liu et al., 2010	6.2	1.9	33	9.8	2.8	30	6.2%	-3.60 [-4.79, -2.41]
da Fonseca et al., 2011	4	3.7	24	7.6	8.1	26	0.0%	-3.60 [-7.05, -0.15]
Dag et al., 2011	5.55	2.35	99	9	6.5	100	5.4%	-3.45 [-4.81, -2.09]
Pragatheeswarane et al., 2014	11.1	5.5	60	14.4	8.5	60	2.3%	-3.30 [-5.86, -0.74]
Ionescu et al., 2009	6.43	3.41	48	9.16	2.67	48	6.0%	-2.73 [-3.96, -1.50]
Mingjie et al.,2017	6.38	2.04	73	8.62	2.87	76	8.4%	-2.24 [-3.04, -1.44]
Wang et al., 2019	8.89	3.27	30	10.76	4.58	30	3.3%	-1.87 [-3.88, 0.14]
Mari et al., 2016	4.7	1.2	40	6	1.6	43	9.6%	-1.30 [-1.91, -0.69]
Zhou et al., 2006	8.4	3.4	161	9.6	5	155	7.5%	-1.20 [-2.15, -0.25]
Li et al., 2014	8.54	3.18	208	9.62	3.83	237	9.3%	-1.08 [-1.73, -0.43]
Feng et al., 2016	7.54	2.18	121	8.62	2.83	120	9.4%	-1.08 [-1.72, -0.44]
Abdikarim et al., 2015	6.8	1.1	30	7.7	1.1	31	9.9%	-0.90 [-1.45, -0.35]
Hartsell et al., 1997	7.2	3.3	29	8.1	2.3	29	0.0%	-0.90 [-2.36, 0.56]
Ren et al., 2012	5.7	1.6	299	6.6	2.4	298	11.1%	-0.90 [-1.23, -0.57]
El Nakeeb et al., 2009	6.2	0.2	60	6.9	0.5	60	0.0%	-0.70 [-0.84, -0.56]
Reissman et al.,1995	6.2	0.2	80	6.8	0.2	81	11.8%	-0.60 [-0.66, -0.54]
**Total (95% CI)**			**1282**			**1309**	**100.0%**	**-1.57 [-2.00, -1.14]**

Heterogeneity: Tau² = 0.41; Chi² = 86.33, df = 12 (P < 0.00001); I² = 86%

Test for overall effect: Z = 7.18 (P < 0.00001)

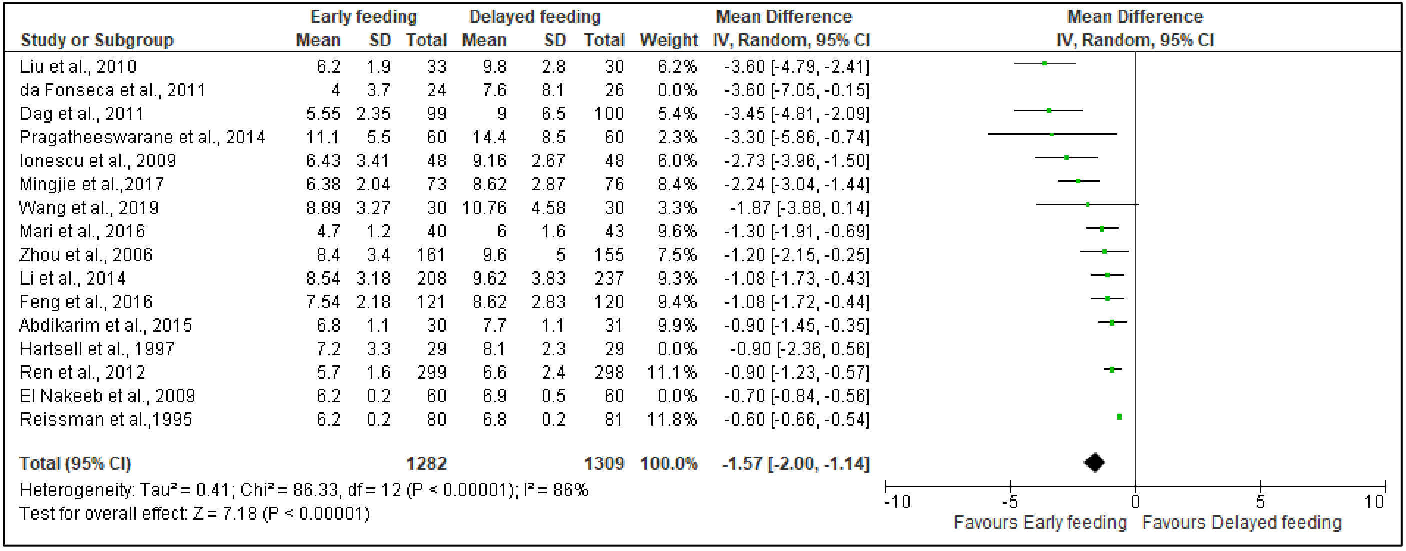

16.B Only studies with more than 100 participants

Study or Subgroup	Early feeding Mean	Early feeding SD	Early feeding Total	Delayed feeding Mean	Delayed feeding SD	Delayed feeding Total	Weight	Mean Difference IV, Random, 95% CI
Liu et al., 2010	6.2	1.9	33	9.8	2.8	30	0.0%	-3.60 [-4.79, -2.41]
da Fonseca et al., 2011	4	3.7	24	7.6	8.1	26	0.0%	-3.60 [-7.05, -0.15]
Dag et al., 2011	5.55	2.35	99	9	6.5	100	3.4%	-3.45 [-4.81, -2.09]
Pragatheeswarane et al., 2014	11.1	5.5	60	14.4	8.5	60	1.1%	-3.30 [-5.86, -0.74]
Ionescu et al., 2009	6.43	3.41	48	9.16	2.67	48	0.0%	-2.73 [-3.96, -1.50]
Mingjie et al.,2017	6.38	2.04	73	8.62	2.87	76	7.7%	-2.24 [-3.04, -1.44]
Wang et al., 2019	8.89	3.27	30	10.76	4.58	30	0.0%	-1.87 [-3.88, 0.14]
Mari et al., 2016	4.7	1.2	40	6	1.6	43	0.0%	-1.30 [-1.91, -0.69]
Zhou et al., 2006	8.4	3.4	161	9.6	5	155	6.0%	-1.20 [-2.15, -0.25]
Li et al., 2014	8.54	3.18	208	9.62	3.83	237	9.9%	-1.08 [-1.73, -0.43]
Feng et al., 2016	7.54	2.18	121	8.62	2.83	120	10.1%	-1.08 [-1.72, -0.44]
Abdikarim et al., 2015	6.8	1.1	30	7.7	1.1	31	0.0%	-0.90 [-1.45, -0.35]
Hartsell et al., 1997	7.2	3.3	29	8.1	2.3	29	0.0%	-0.90 [-2.36, 0.56]
Ren et al., 2012	5.7	1.6	299	6.6	2.4	298	17.2%	-0.90 [-1.23, -0.57]
El Nakeeb et al., 2009	6.2	0.2	60	6.9	0.5	60	21.8%	-0.70 [-0.84, -0.56]
Reissman et al.,1995	6.2	0.2	80	6.8	0.2	81	22.8%	-0.60 [-0.66, -0.54]
**Total (95% CI)**			**1161**			**1187**	**100.0%**	**-1.06 [-1.33, -0.79]**

Heterogeneity: Tau² = 0.08; Chi² = 45.77, df = 8 (P < 0.00001); I² = 83%

Test for overall effect: Z = 7.65 (P < 0.00001)

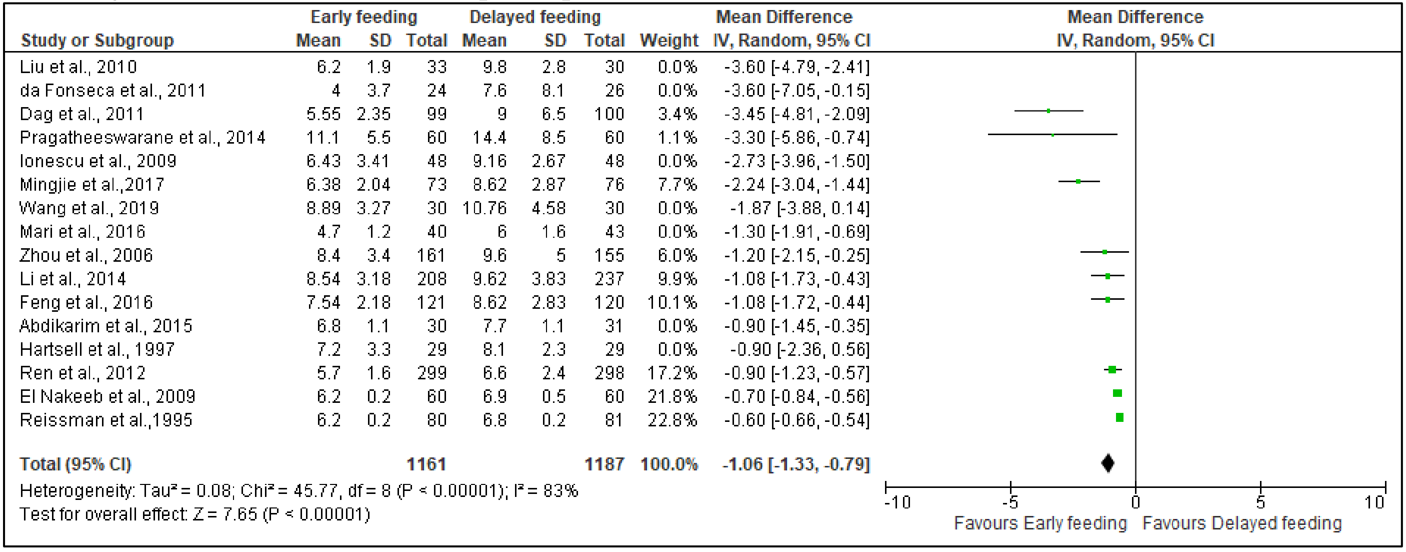

Supplementary File 17. Sensitivity analysis for the outcome “Complications”

17.A Only moderate or low risk of bias studies

Study or Subgroup	Early feeding Events	Early feeding Total	Routine care Events	Routine care Total	Weight	Risk Ratio M-H, Random, 95% CI
Wang et al., 2012	2	40	8	38	1.2%	0.24 [0.05, 1.05]
Zhou et al., 2006	21	161	66	155	6.2%	0.31 [0.20, 0.48]
Feng et al., 2016	7	121	17	120	3.0%	0.41 [0.18, 0.95]
Wendler et al., 2022	3	40	7	40	1.6%	0.43 [0.12, 1.54]
Liang et al., 2018	18	208	47	237	5.5%	0.44 [0.26, 0.73]
Li et al., 2019	12	100	25	100	4.4%	0.48 [0.26, 0.90]
da Fonseca et al., 2011	4	24	9	26	0.0%	0.48 [0.17, 1.36]
Lee et al., 2011	6	46	14	54	2.9%	0.50 [0.21, 1.20]
Abdikarim et al., 2015	1	30	2	31	0.5%	0.52 [0.05, 5.40]
Mari et al., 2016	1	40	2	43	0.5%	0.54 [0.05, 5.70]
Ionescu et al., 2009	6	48	11	48	2.7%	0.55 [0.22, 1.36]
Khoo et al., 2007	9	41	16	40	3.9%	0.55 [0.28, 1.09]
Cao et al., 2021	22	85	39	86	6.4%	0.57 [0.37, 0.88]
Liu et al., 2010	4	33	6	30	1.8%	0.61 [0.19, 1.94]
El Nakeeb et al., 2009	14	60	23	60	0.0%	0.61 [0.35, 1.07]
Li et al., 2014	21	58	34	61	6.6%	0.65 [0.43, 0.98]
Pragatheeswarane et al., 2014	24	60	33	60	6.9%	0.73 [0.49, 1.07]
Kang et al., 2018	6	46	9	51	0.0%	0.74 [0.28, 1.92]
Consoli et al., 2010	4	15	5	14	2.0%	0.75 [0.25, 2.23]
Wang et al., 2019	7	30	9	30	3.0%	0.78 [0.33, 1.82]
Stewart et al., 1998	10	40	12	40	3.8%	0.83 [0.41, 1.70]
Dag et al., 2011	12	99	14	100	3.7%	0.87 [0.42, 1.78]
Geubbels et al., 2019	7	110	8	110	2.4%	0.88 [0.33, 2.33]
Shichinohe et al., 2017	9	45	11	49	3.3%	0.89 [0.41, 1.95]
Feo et al., 2004	12	50	13	50	4.0%	0.92 [0.47, 1.82]
He et al., 2015	7	48	6	38	2.3%	0.92 [0.34, 2.52]
Hwang et al., 2019	64	123	68	124	8.8%	0.95 [0.75, 1.20]
Ren et al., 2012	28	299	29	298	5.6%	0.96 [0.59, 1.58]
Ortiz et al., 1996	17	93	18	95	0.0%	0.96 [0.53, 1.75]
Hartsell et al., 1997	1	29	1	29	0.0%	1.00 [0.07, 15.24]
Reissman et al.,1995	6	80	5	81	1.9%	1.22 [0.39, 3.82]
Lee et al., 2013	22	52	11	46	4.6%	1.77 [0.97, 3.24]
Mingjie et al.,2017	2	73	1	76	0.5%	2.08 [0.19, 22.47]
**Total (95% CI)**		**2175**		**2199**	**100.0%**	**0.68 [0.57, 0.81]**
Total events	347		519			

Heterogeneity: Tau² = 0.08; Chi² = 46.82, df = 27 (P = 0.01); I² = 42%
Test for overall effect: Z = 4.31 (P < 0.0001)

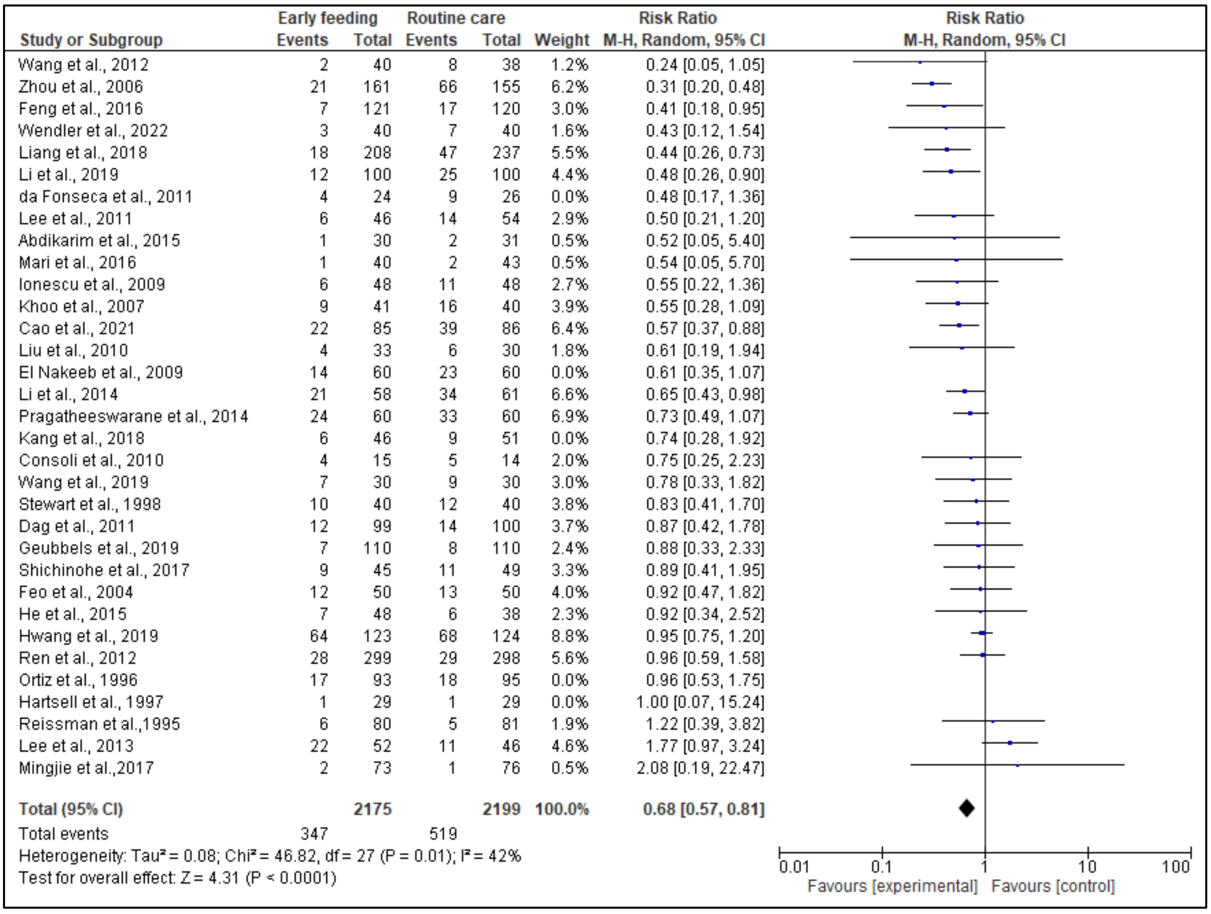

17.B Only studies with more than 100 participants

Study or Subgroup	Early feeding Events	Early feeding Total	Routine care Events	Routine care Total	Weight	Risk Ratio M-H, Random, 95% CI
Wang et al., 2012	2	40	8	38	0.0%	0.24 [0.05, 1.05]
Zhou et al., 2006	21	161	66	155	7.6%	0.31 [0.20, 0.48]
Feng et al., 2016	7	121	17	120	4.2%	0.41 [0.18, 0.95]
Wendler et al., 2022	3	40	7	40	0.0%	0.43 [0.12, 1.54]
Liang et al., 2018	18	208	47	237	6.8%	0.44 [0.26, 0.73]
Li et al., 2019	12	100	25	100	5.7%	0.48 [0.26, 0.90]
da Fonseca et al., 2011	4	24	9	26	0.0%	0.48 [0.17, 1.36]
Lee et al., 2011	6	46	14	54	0.0%	0.50 [0.21, 1.20]
Abdikarim et al., 2015	1	30	2	31	0.0%	0.52 [0.05, 5.40]
Mari et al., 2016	1	40	2	43	0.0%	0.54 [0.05, 5.70]
Ionescu et al., 2009	6	48	11	48	0.0%	0.55 [0.22, 1.36]
Khoo et al., 2007	9	41	16	40	0.0%	0.55 [0.28, 1.09]
Cao et al., 2021	22	85	39	86	7.7%	0.57 [0.37, 0.88]
Liu et al., 2010	4	33	6	30	0.0%	0.61 [0.19, 1.94]
El Nakeeb et al., 2009	14	60	23	60	6.3%	0.61 [0.35, 1.07]
Li et al., 2014	21	58	34	61	7.9%	0.65 [0.43, 0.98]
Pragatheeswarane et al., 2014	24	60	33	60	8.1%	0.73 [0.49, 1.07]
Kang et al., 2018	6	46	9	51	0.0%	0.74 [0.28, 1.92]
Consoli et al., 2010	4	15	5	14	0.0%	0.75 [0.25, 2.23]
Wang et al., 2019	7	30	9	30	0.0%	0.78 [0.33, 1.82]
Stewart et al., 1998	10	40	12	40	0.0%	0.83 [0.41, 1.70]
Dag et al., 2011	12	99	14	100	5.0%	0.87 [0.42, 1.78]
Geubbels et al., 2019	7	110	8	110	3.4%	0.88 [0.33, 2.33]
Shichinohe et al., 2017	9	45	11	49	0.0%	0.89 [0.41, 1.95]
Feo et al., 2004	12	50	13	50	5.3%	0.92 [0.47, 1.82]
He et al., 2015	7	48	6	38	0.0%	0.92 [0.34, 2.52]
Hwang et al., 2019	64	123	68	124	9.7%	0.95 [0.75, 1.20]
Ren et al., 2012	28	299	29	298	7.0%	0.96 [0.59, 1.58]
Ortiz et al., 1996	17	93	18	95	6.0%	0.96 [0.53, 1.75]
Hartsell et al., 1997	1	29	1	29	0.0%	1.00 [0.07, 15.24]
Reissman et al.,1995	6	80	5	81	2.7%	1.22 [0.39, 3.82]
Lee et al., 2013	22	52	11	46	5.9%	1.77 [0.97, 3.24]
Mingjie et al.,2017	2	73	1	76	0.8%	2.08 [0.19, 22.47]
**Total (95% CI)**		**1832**		**1859**	**100.0%**	**0.71 [0.57, 0.89]**
Total events	309		451			

Heterogeneity: Tau² = 0.11; Chi² = 42.60, df = 16 (P = 0.0003); I² = 62%
Test for overall effect: Z = 3.05 (P = 0.002)

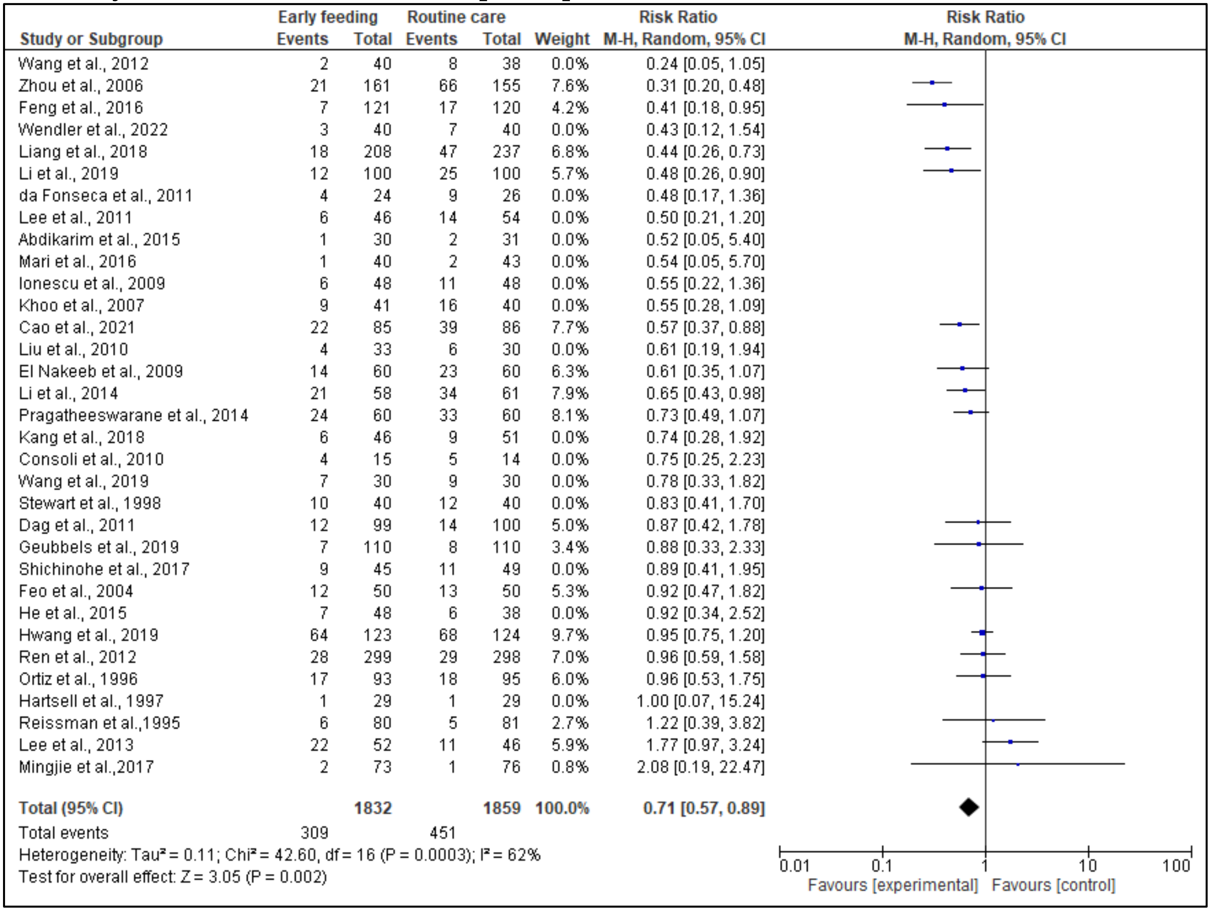

Supplementary File 18. Sensitivity analysis for the outcome "Vomiting"

18.A Only moderate or low risk of bias studies

Study or Subgroup	Early feeding Events	Early feeding Total	Routine care Events	Routine care Total	Weight	Risk Ratio M-H, Random, 95% CI
Liang et al., 2017	6	58	15	61	10.2%	0.42 [0.18, 1.01]
Wu et al., 2019	14	867	31	868	0.0%	0.45 [0.24, 0.84]
Feo et al., 2004	7	50	14	50	11.5%	0.50 [0.22, 1.13]
Pragatheeswarane et al., 2014	5	60	7	60	6.8%	0.71 [0.24, 2.13]
Ionescu et al., 2009	17	48	21	48	25.8%	0.81 [0.49, 1.33]
Da Fonseca et al., 2010	4	24	5	26	0.0%	0.87 [0.26, 2.85]
Liu et al., 2010	1	33	1	30	1.2%	0.91 [0.06, 13.90]
Stewart et al., 1998	14	40	14	40	19.5%	1.00 [0.55, 1.82]
Hartsell et al., 1997	14	29	10	29	0.0%	1.40 [0.75, 2.62]
El Nakeeb et al., 2009	15	60	10	60	0.0%	1.50 [0.73, 3.07]
Wang et al., 2019	3	30	2	30	2.9%	1.50 [0.27, 8.34]
Shichinohe et al., 2017	7	45	5	49	7.0%	1.52 [0.52, 4.46]
Reissman et al.,1995	17	80	11	81	15.3%	1.56 [0.78, 3.13]
**Total (95% CI)**		**444**		**449**	**100.0%**	**0.87 [0.65, 1.17]**
Total events	77		90			

Heterogeneity: Tau² = 0.02; Chi² = 9.02, df = 8 (P = 0.34); I² = 11%
Test for overall effect: Z = 0.91 (P = 0.37)

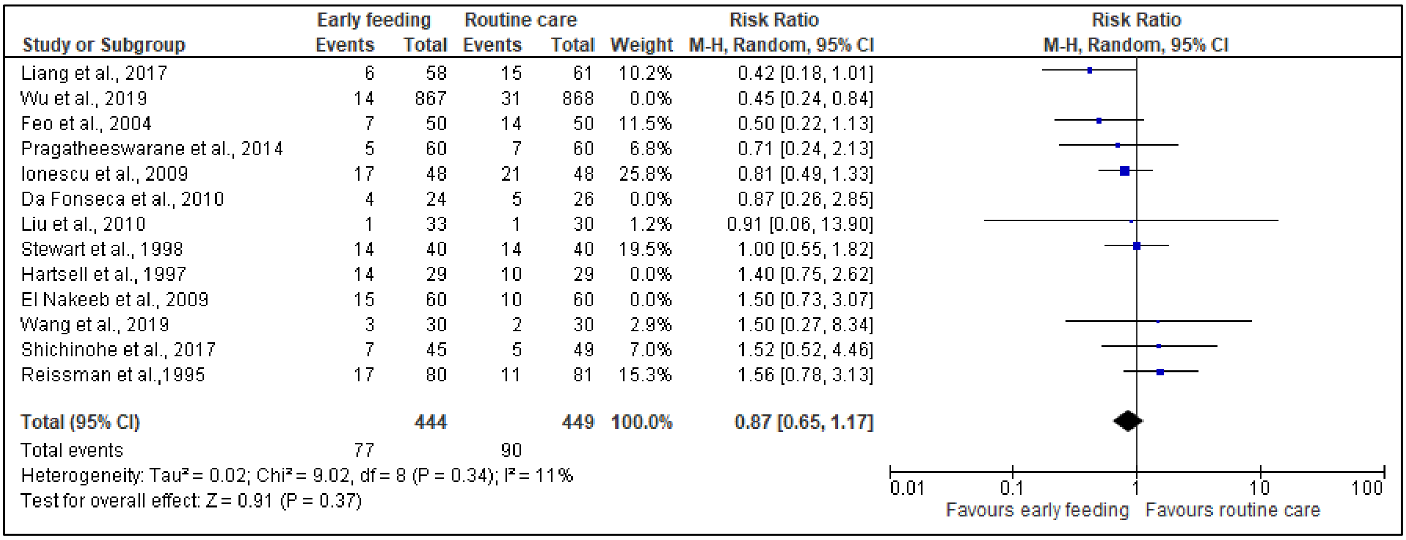

18.B Only studies with more than 100 participants

Study or Subgroup	Early feeding Events	Early feeding Total	Routine care Events	Routine care Total	Weight	Risk Ratio M-H, Random, 95% CI
Liang et al., 2017	6	58	15	61	15.4%	0.42 [0.18, 1.01]
Wu et al., 2019	14	867	31	868	19.6%	0.45 [0.24, 0.84]
Feo et al., 2004	7	50	14	50	16.3%	0.50 [0.22, 1.13]
Pragatheeswarane et al., 2014	5	60	7	60	12.4%	0.71 [0.24, 2.13]
Ionescu et al., 2009	17	48	21	48	0.0%	0.81 [0.49, 1.33]
Da Fonseca et al., 2010	4	24	5	26	0.0%	0.87 [0.26, 2.85]
Liu et al., 2010	1	33	1	30	0.0%	0.91 [0.06, 13.90]
Stewart et al., 1998	14	40	14	40	0.0%	1.00 [0.55, 1.82]
Hartsell et al., 1997	14	29	10	29	0.0%	1.40 [0.75, 2.62]
El Nakeeb et al., 2009	15	60	10	60	18.0%	1.50 [0.73, 3.07]
Wang et al., 2019	3	30	2	30	0.0%	1.50 [0.27, 8.34]
Shichinohe et al., 2017	7	45	5	49	0.0%	1.52 [0.52, 4.46]
Reissman et al.,1995	17	80	11	81	18.4%	1.56 [0.78, 3.13]
**Total (95% CI)**		**1175**		**1180**	**100.0%**	**0.75 [0.45, 1.26]**
Total events	64		88			

Heterogeneity: Tau² = 0.25; Chi² = 13.13, df = 5 (P = 0.02); I² = 62%
Test for overall effect: Z = 1.09 (P = 0.28)

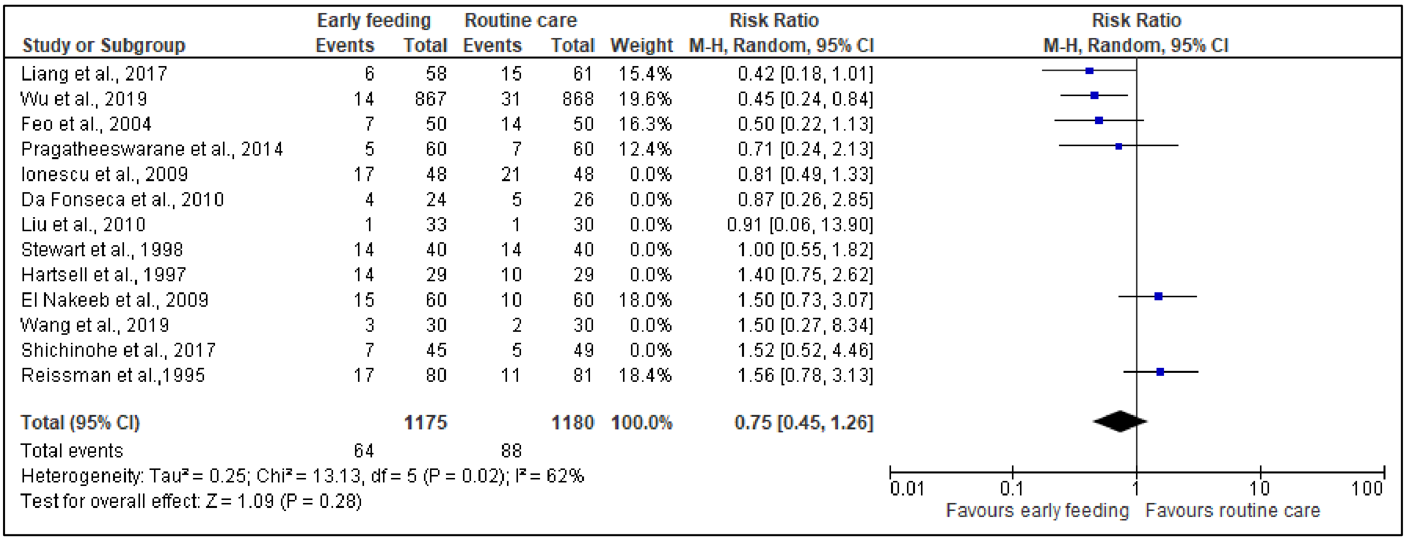

Supplementary file 19. Differences in outcomes by type of diet in the first postoperative day

			Outcomes				
**Intervention**	**Author, year**	**Type of feeding at POD1**	**TF Passage of Stool**	**TF Flatus**	**LOS**	**Complications**	**Vomiting**
		**Liquid diet**					
**Early feeding**	Hartsell et al., 1997	Full liquid diet			🔴	🔴	🔴
	Zhou et al., 2006	Water 12-24 h after the removal of nasogastric tube	🟢	🟢	🟢	🟢	
	El Nakeeb et al., 2009	Fluids	🟢	🟢	🟢	🔴	🔴
	Wu et al., 2019	Water					🟢
	Reissman et al., 1995	Clear liquid diet			🟢	🔴	🔴
	Pragatheeswarane et al., 2014	Clear liquid diet of 30 cm3 /h at the 24th hour	🟢	🟢	🟢	🔴	🔴
	Da Fonseca et al., 2010	Oral liquid diet (approximately 500 cm3)			🟢		🔴
**Multimodal interventions**	Feo et al., 2004	Liquids	🔴		🔴		🔴
	Wang et al., 2012	Fluid diet		🟢	🟢	🔴	🔴
	Wendler et al., 2022	Liquid diet + ringer lactose and glucose solution		🔴		🔴	
**ERAS**	Abdikarim et al., 2015	Fluids	🟢		🟢	🔴	
	Mingjie et al., 2017	Oral fluids 0,5 L; I/V fluids 1ml/Kg/h	🟢		🟢	🔴	
	Kang et al., 2018	Sips of water if tolerable		🟢	🟢	🔴	
	Wang et al., 2019	Oral fluid intake 500 mL, caloric intake 25–30 kcal/kg	🟢	🟢	🔴	🔴	🔴
	He et al., 2015	Liquid diet restored 12 h after surgery		🟢	🟢	🔴	
	Cao et al., 2021	Clear liquid diet at dinner		🟢	🟢	🟢	
		**Liquids + nutrients**					
**Multimodal interventions**	Li et al., 2014	Water or tea at 12 h, EN emulsion (Fresubin®; 25-30 kcal/kg·d)	🟢	🟢	🟢	🟢	
	Feng et al., 2016	Water or tea at 12 h, EN emulsion (Fresubin®; 25-30 kcal/kg·d)	🟢	🟢	🟢	🟢	
	Shichinohe eta al., 2017	Water and ED 900 mL/day; parenteral nutrition 500 mL;	🟢	🔴	🔴	🔴	🔴
	Sun et al., 2017	Enteral nutrition suspension (300 ml; Peptisorb liquid, Nutricia)	🟢	🟢	🟢		
**ERAS**	Ren et al., 2012	1,000 ml water + 500 ml nutritional supplements		🟢	🟢	🔴	
		**Semifluid diet**					
**Multimodal interventions**	Liu et al., 2010	Semiliquid diet 50–100 mL + GS 10% 1000 mL and GN 500 mL		🟢	🟢	🔴	🔴
	Lee et al., 2011	Semifluid diet >1 L	🔴	🔴	🔴		
	Lee et al., 2013	Semi-fluid diet	🟢	🟢	🔴		
**ERAS**	Liang et al., 2018	Liquid oral nutritional supplements or semi-liquid diet		🟢	🟢	🟢	🔴
		**Solid diet or restrictions not specified**					
**Early feeding**	Dag et al., 2011	Fluid diet 12 hours; a solid diet as tolerated	🟢		🟢	🔴	
	Consoli et al., 2010	500 ml fluid and if no nausea and vomits free diet		🟢	🟢	🔴	
	Ortiz et al., 1996	Regular diet as desired				🔴	🟢
	Stewart et al., 1998	Solid diet at their own discretion		🟢	🔴	🔴	🔴
**Multimodal interventions**	Ionescu et al., 2009	Fluids, Solid food (yogurt, cheese)			🟢	🔴	🔴
	Khoo et al., 2007	Diet was allowed immediately after the operation	🟢		🟢		
**ERAS**	Mari et al., 2016	Oral feeding		🟢	🟢	🔴	
	Geubbels et al., 2019	(Time not specified) Early oral feeding			🔴	🔴	🟢
	Hwang et al., 2019	(Time not specified) Early oral intake			🔴	🔴	
	Li et al., 2019	(Time not specified) Avoided excessive fluids	🟢			🔴	

Note: TF, Time to the first; Green circle: results in favour of intervention; Red circle: No statistically significant differences between intervention and control groups
